# Supplementary material for: Metabolic engineering and late-stage functionalization expand the chemical space of the antimalarial premarineosin A
Source: Commun Chem. 2025 Dec 5;8:391. doi: 10.1038/s42004-025-01779-6 (PMC12680612; doi:10.1038/s42004-025-01779-6)
Supplement: Supplementary file 1 — Supplementary Information [file 42004_2025_1779_MOESM1_ESM.pdf]

Supplementary Information

Metabolic engineering and late-stage functionalization expand the chemical space of the antimalarial premarineosin A

Christina M. McBride[a,b,†], Morgan McCauley[a,†], Natalia R. Harris[a,c], Sahar Amin[a,d], Brian J. Curtis[a], Linnea Verhey-Henke[a], Awet A. Teklemichael[e], Erin N. Oliphant[f], Patricia Dranchak[f], Katherine L. Lev[a,b], Fengrui Qu[g], Harrison M. Snodgrass[h], Jared C. Lewis[h,*], James Inglese[f,i], Xin-zhuan Su[e], Filipa Pereira[a,c,#,*], David H. Sherman[a,d,g,j,#,*]

[a] *Life Sciences Institute, University of Michigan, Ann Arbor, Michigan, USA*
[b] *Program in Chemical Biology, University of Michigan, Ann Arbor, Michigan, USA*
[c] *Department of Biological Chemistry, University of Michigan Medical School, Ann Arbor, Michigan, USA*
[d] *Department of Medicinal Chemistry, University of Michigan, Ann Arbor, Michigan, USA*
[e] *Laboratory of Malaria and Vector Research, National Institute of Allergy and Infectious Diseases, NIH, Rockville, Maryland, USA*
[f] *National Center for Advancing Translational Sciences (NCATS), NIH, Rockville, Maryland, USA*
[g] *Department of Chemistry, University of Michigan, Ann Arbor, Michigan, USA*
[h] *Department of Chemistry, Indiana University, Bloomington, Indiana, USA*
[i] *Metabolic Medicine Branch, National Human Genome Research Institute, NIH, Bethesda, Maryland, USA*
[j] *Department of Microbiology and Immunology, University of Michigan, Ann Arbor, Michigan, USA*

[†] *These authors contributed equally.*
[#] *These authors jointly supervised this work*
[*] *Corresponding author. E-mail address: davidhs@umich.edu, fpere@umich.edu, jcl3@iu.edu*

Table of Contents

Supplementary Methods 3
Supplementary Methods 1. Untargeted Metabolomics Methods **3**
Sample Preparation for Untargeted Metabolomics 3
UHPLC-QTOF-MS/MS profiling of crude extracts 3
Metabolomic data processing 3
Supplementary Methods 2. Characterization of Isolated Natural Products **4-5**
Marineosin A (**1**) 4
23-Hydroxyundecylprodiginine (**2**) 4
(−)-Premarineosin A (**3**) 4
Crystallization method for (−)-premarineosin A (**3**) 5
Supplementary Methods 3. Synthetic Chemistry Methods **6-10**
(−)-Premarineosin A derivatization general procedure 6
Gem-dimethyl-bridged premarineosin A (**3**) 6
Crystallization method for gem-dimethyl-bridged premarineosin A (**4**) 6
Methylene-bridged premarineosin A (**5**) 7
Trifluoromethyl-bridged premarineosin A (**6**) 7-8
12-trifluoropropanol premarineosin A (**7**) 7-8
12-formyl premarineosin A (**8**) 8
12-hydroxymethyl premarineosin A (**10**) 9
12-acetyl premarineosin A (**9**) 9
1-bromo premarineosin A (**11**) 10
12-bromo premarineosin A (**12**) 10
Supplementary Tables **11-12**
Supplementary Table 1. (−)-Premarineosin A biosynthetic genes in *S. eitanensis* and their homology to the *Streptomyces* sp. CNQ-617 *mar* BGC genes 11
Supplementary Table 2. Strains used in this work. 12
Supplementary Table 3. Plasmids used in this work 12
Supplementary Table 4. Primers used in this work 12
Supplementary Table 5. Gene sequences used in this work 13
Supplementary Figures **14-18**
Supplementary Figure 1. Untargeted metabolic analysis of *S. eitanensis* 14
Supplementary Figure 2. Integration of plasmids containing *pmaD* and *pmaG* in the *Streptomyces eitanensis* genome 14
Supplementary Figure 3. Effect of crude complex protein source on yield of (−)-premarineosin A (**3**) in 1mL extracts from 50 mL cultures of FP10018 15
Supplementary Figure 4. (−)-Premarineosin A (**3**) and 23-HUP (**2**) production (mg/L) increase over time 15
Supplementary Figure 5. Extracting with acetone improves (−)-premarineosin A (**3**) titers 15
Supplementary Figure 6. Purity analysis of replicate isolated yields of (−)-premarineosin A (**3**) 16
Supplementary Figure 7. Formation of the 12-trifluoropropanol premarineosin A (**7**) monomer 16
Supplementary Figure 8. LC-MS/MS characterization of 1-bromo (**11**) and 12-bromo premarineosin A (**12**) 17
Supplementary Figure 9. Mammalian cell cytotoxicity in HEK293 embryonic kidney and MOLT4 T-cell leukemia cell lines 18
Supplementary Figure 10. Activity of artemisinin, chloroquine, (−)-premarineosin A (**3**), and derivatives **4-12** against *P. falciparum* 3D7 (chloroquine-sensitive) and Dd2 (chloroquine-resistant) parasite lines .. 18
Supplementary Notes: X-Ray Crystallography Data **19-31**
Supplementary Note 1. X-Ray Crystal Structure Determination for (−)-premarineosin A (**3**) 19-24
Supplementary Note 2. X-Ray Crystal Structure Determination for Gem-dimethyl-bridged premarineosin A (**4**) 25-31
Supplementary References **32-33**

Supplementary Methods.

Supplementary Methods 1. Untargeted Metabolomics Methods.

Sample preparation for untargeted metabolomics. Intracellular metabolite extracts were used for untargeted metabolomics. The culture medium was extracted as a control and run alongside experimental samples to account for sampling and laboratory contamination. Reagent blanks (MeOH) were prepared and analyzed to account for extraneous signals occurring from chemicals and analytical systems. Three biological replicates were prepared for each strain to demonstrate reproducibility.

UHPLC-QTOF-MS/MS profiling of crude extracts. Metabolite extracts were analyzed using ultra-high-performance liquid chromatography coupled with quadrupole time-of-flight mass spectrometry (UHPLC-LCMS) performed with an Agilent 1290 Infinity II UHPLC coupled to an Agilent 6545 ESI-Q-TOF-MS. The samples were injected, and data was collected using auto-MS/MS in positive mode. The chromatographic separation was carried out on a Phenomenex Kinetex Phenyl-Hexyl column (1.7 µm, 2.1 × 100 mm). Isocratic elution was performed with 90% solvent A (water + 0.1% formic acid) for 1 min, followed by a 9 min linear gradient to 100% solvent B (95% acetonitrile + 5% water + 0.1% formic acid). Electrospray ionization was performed at a capillary temperature of 320 °C, with a source voltage of 3.5 kV, and a sheath gas flow rate of 11 L/min. Ion fragmentation utilized ramped collision energy (5 x m/z/100 + 10 eV) for up to nine selected precursor ions per cycle. Purine $C_5H_4N_4$ $[M + H]^+$ (*m/z* 121.0508) and hexakis(1H,1H,3H-tetrafluoropropoxy)-phosphazene $C_{18}H_{18}F_{24}N_3O_6P_3$ $[M + H]^+$ (*m/z* 922.0098) served as internal lock standards, and these ions were added to the static exclusion list. This exclusion list was refined by identifying high-intensity contaminant ions (>1000 counts) present in blank solvent samples.

Metabolomic data processing. The raw mass spectrometry data was converted from vendor-specific instrument files to mzML format using the msConvert software from ProteoWizard[1] with standard peak detection settings. Subsequent data processing was conducted in MZmine3[2] using an untargeted LC-MS/MS preprocessing workflow from the Processing Wizard. HPLC retention time was selected between 0.4 and 9 min, allowing for a maximum of 15 peaks in the chromatogram and requiring at least four consecutive scans. The full width at half maximum (FWHM) for features was set at approximately 0.045 min. Retention time (RT) tolerance was defined as 0.08 min for intra-sample variations and 0.4 min for sample-to-sample differences. The noise levels were set at $5.0x10^2$ for MS1 and $2.0x10^2$ for MS2. The minimum feature height was established at $1.0x10^3$, with a scan-to-scan m/z tolerance of 0.005 m/z (20 ppm), an intra-sample m/z tolerance of 0.0015 m/z (3 ppm), and a sample-to-sample m/z tolerance of 0.004 m/z (8 ppm). The output, including the feature quantification table, peak list, and metadata table, was processed through feature-based molecular networking on the Global Natural Products Social Molecular Networking (GNPS)[3] platform. MS/MS spectra of molecular features were tentatively matched to the GNPS MS/MS Spectral Libraries if they had a cosine similarity score greater than 0.7 and shared at least 4 peaks. The resulting molecular network was visualized using Cytoscape 3.9.1.[4]

Supplementary Methods 2. Characterization of Isolated Natural Products.

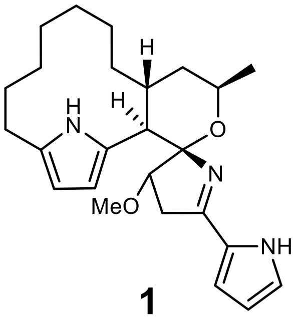

1

Marineosin (1). ^{1}H NMR (600 MHz, acetone-D_6) δ 10.98 (br s, 1H), 8.25 (br s, 1H), 6.99 (s, 1H), 6.39 (s, 1H), 6.13 (s, 1H), 5.69 (s, 1H), 5.45 (s, 1H), 4.27 – 4.20 (m, 1H), 3.86 (t, *J* = 8.5 Hz, 1H), 3.42 (s, 3H), 2.91 (d, *J* = 12.1 Hz, 2H), 2.38 – 2.29 (m, 1H), 2.28 – 2.18 (m, 2H), 1.89 (dd, *J* = 16.2, 8.5 Hz, 1H), 1.78 (d, *J* = 13.2 Hz, 1H), 1.70 – 1.58 (m, 4H), 1.52 (*J* = 6.8 Hz, 3H), 1.43 – 1.24 (m, 4H), 1.26 – 1.16 (m, 1H), 1.07 – 1.00 (m, 1H), 0.93 – 0.83 (m, 2H), 0.74 – 0.66 (m, 1H), 0.56 – 0.47 (m, 1H). **^{13}C NMR (151 MHz, acetone-D_6)** δ 164.41, 131.71, 129.88, 129.12, 122.63, 113.55, 110.24, 110.03, 106.04, 104.75, 89.93, 70.43, 58.51, 45.90, 39.60, 39.03, 31.95, 28.64, 28.05, 26.03 – 25.02 (m), 22.82. **LC-MS:** calculated for $C_{25}H_{35}N_3O_2$ [M+H$^+$] 410.2763 m/z; found 410.2823 m/z. The NMR spectrum is nearly identical to those previously reported[5,6], but some differences (e.g. signal broadening of pyrrole protons) was observed.

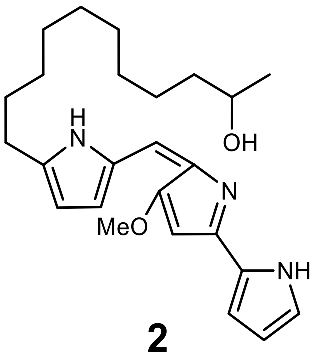

2

23-Hydroxyundecylprodiginine (2). ^{1}H NMR (600 MHz, acetone-D_6) δ 7.36 – 7.32 (m, 1H), 7.21 – 7.18 (m, 1H), 7.14 – 7.10 (m, 1H), 7.02 (br s, 1H), 6.60 (s, 1H), 6.40 (dd, *J* = 3.4, 2.5 Hz, 1H), 6.28 (d, *J* = 3.8 Hz, 1H), 4.09 (s, 3H), 3.68 (m, 2H), 2.89 (t, *J* = 7.7 Hz, 2H), 1.78 (quintet, *J* = 7.4 Hz, 2H), 1.43 – 1.27 (m, 16H), 1.09 (d, *J* = 6.1 Hz, 3H). **LC-MS:** calculated for $C_{25}H_{35}N_3O_2$ [M+H$^+$] 410.2763 m/z; found 410.2812 m/z. The discrepancies in the alkene signals with respect to reported data[6] are due to purification under acidic conditions. The alkene signals are near identical to those reported for the HCl salt of undecylprodiginine, though this analyzed sample contains the corresponding -OH group.

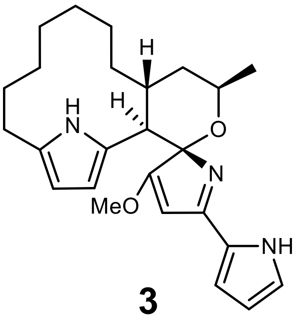

3

(−)-Premarineosin A* (3). **^{1}H NMR (600 MHz, acetone-D$_6$)** δ 13.67 (br s, 1H), 12.06 (br s, 1H), 9.82 (br s, 1H), 7.67 – 7.65 (m, 1H), 7.49 – 7.44 (m, 1H), 6.54 – 6.50 (m, 1H), 6.29 – 6.26 (m, 1H), 5.72 (t, *J* = 2.9 Hz, 3H), 5.52 (t, *J* = 2.9 Hz, 1H), 4.46 – 4.38 (m, 1H), 4.15 (s, 3H), 3.03 (d, *J* = 12.5 Hz, 1H), 2.69 (dp, *J* = 13.4, 4.9 Hz, 1H), 2.39 (dt, *J* = 14.8, 4.4 Hz, 1H), 2.28 (ddd, *J* = 14.7, 11.7, 3.3 Hz, 1H), 1.95 (dt, *J* = 13.8, 4.2 Hz, 1H), 1.92 – 1.83 (m, 2H), 1.72 (ddd, *J* = 14.5, 10.1, 5.1 Hz, 1H), 1.45 (d, *J* = 6.8 Hz, 3H), 1.43 – 1.36 (m, 4H), 1.36 – 1.30 (m, 1H), 1.25 – 1.17 (m, 1H), 1.11 – 1.01 (m, 2H), 0.89 – 0.77 (m, 2H), 0.60 – 0.51 (m, 1H). **^{13}C NMR (151 MHz, acetone-D$_6$)** δ 181.77, 165.95, 163.25 (q, *J* = 35.36 Hz), 135.05, 134.18, 127.55, 125.09, 121.64, 117.74 (q, *J* = 292.0 Hz), 114.62, 110.64, 105.99, 97.24, 93.84, 71.23, 60.92, 46.32, 38.27, 33.56, 28.70, 28.19, 27.26, 25.87, 25.23, 25.18, 24.82, 20.92. **LC-MS:** calculated for $C_{25}H_{33}N_3O_2$ [M+H$^+$] 408.2651 m/z; found 408.2673 m/z. *characterized as the trifluoroacetic acid salt, explaining discrepancies with respect to previously reported[6] NMR data.

Crystallization method for (−)-premarineosin A. A (−)-premarineosin A sample that was purified via chromatography (see Materials and Methods) was dissolved in a minimal amount of ethyl acetate. Vapor diffusion with hexanes was performed over the course of one day to give colorless, prism-shaped crystals.

Supplementary Methods 3. Synthetic Chemistry Methods.

(−)-Premarineosin A derivatization general procedure. (−)-Premarineosin A (**3**) was dissolved in MeCN before TFA and a carbonyl was added at RT. Reaction was run until TLC (50% ethyl acetate in hexanes with 1% acetic acid) showed consumption of starting material (15 min - 6 h). After completion, reaction was diluted with ethyl acetate and washed with 1M NaOH to quench TFA. The aqueous layer was further extracted with ethyl acetate (x3) and organics were combined, dried over sodium sulfate, filtered, and concentrated. The material then was purified through a combination of acid and/or base conditions. Acidic conditions: 50% ethyl acetate (with 1% acetic acid) and hexanes (with 1% acetic acid). Basic conditions: 20-50% of a (3% ammonia (7 M in methanol) in DCM solution) in hexanes gradient.

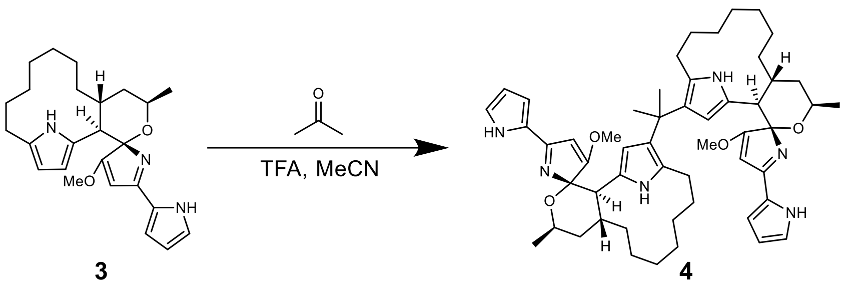

Gem-dimethyl-bridged premarineosin A (4). Amounts: **3** (15.0 mg, 36.8 μmol, 1 equiv), MeCN (1.50 mL), acetone (100 μL, 1.36 mmol, 37 equiv), TFA (113 μL, 1.47 mmol, 40 equiv). Reaction time was 2 h. Starting material was recovered (2.0 mg, 4.9 μmol, 13%) and product (8.0 mg, 9.7 μmol, 26%). Reaction is also successful when ran neat in acetone with same equivalence of TFA and equivalent product yields. **^{1}H NMR (600 MHz, acetone-D$_6$)** δ 7.97 (br s, 2H), 6.99 – 6.89 (m, 2H), 6.51 – 6.39 (m, 2H), 6.22 – 6.04 (m, 2H), 5.53 (s, 2H), 5.34 (s, 2H), 4.31 – 4.22 (m, 2H), 3.76 (s, 6H), 2.63 (d, *J* = 11.9 Hz, 2H), 2.55 – 2.43 (m, 2H), 1.80 (d, *J* = 13.1 Hz, 2H), 1.68 – 1.57 (m, 4H), 1.56 – 1.51 (m, 2H), 1.50 (d, *J* = 6.7 Hz, 6H), 1.43 – 1.36 (m, 4H), 1.29 (s, 6H), 1.27 – 1.08 (m, 10H), 1.04 – 0.98 (m, 2H), 0.97 – 0.90 (m, 2H), 0.88 – 0.80 (m, 2H), 0.78 – 0.68 (m, 2H), 0.65 – 0.56 (m, 2H). **13C NMR (151 MHz, acetone-D$_6$)** δ 181.70, 164.97, 129.10, 128.55, 127.53, 124.92, 122.99, 113.43, 109.94, 106.58, 102.41, 94.80, 70.64, 58.82, 48.28, 39.89, 33.32, 33.11, 30.17, 27.83, 25.86, 25.72, 25.51 24.98, 22.98. **LC-MS:** calculated for $C_{53}H_{70}N_6O_4$ [M+H$^+$] 855.5537 m/z; found 855.5551 m/z.

Crystallization method for gem-dimethyl-bridged (−)-premarineosin A (4). Compound was dissolved in minimal amounts of ethyl acetate. Vapor diffusion with hexanes was performed over the course of two weeks to give orange, irregular crystals.

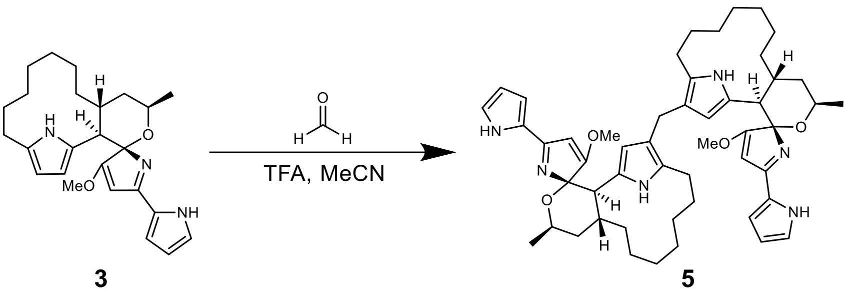

Methylene-bridged premarineosin A (5). Amounts: **3** (15.0 mg, 36.8 µmol, 1 equiv), MeCN (1.50 mL), formaldehyde (151 µL, 2.02 mmol, 55 equiv, 37% in water), TFA (113 µL, 1.47 mmol, 40 equiv). Reaction time was 10-20 minutes. Product yield: 5 mg (36.8 µmol, 20%). **^{1}H NMR (600 MHz, acetone-D$_6$)** δ 8.17 (br s, 2H) 7.05 – 6.98 (m, 2H), 6.60 – 6.53 (m, 2H), 6.20 – 6.12 (m, 2H), 5.46 (s, 2H), 5.43 (s, 2H), 4.31 – 4.23 (m, 2H), 3.76 (s, 6H), 3.11 (s, 2H), 2.64 – 2.60 (m, 2H), 2.46 (d, *J* = 11.9 Hz, 2H), 2.49 – 2.41 (m, 2H), 2.37 (ddd, *J* = 14.7, 11.0, 3.5 Hz, 2H), 2.12 – 2.06 (m, 2H), 1.86 – 1.78 (m, 2H), 1.67 – 1.60 (m, 2H), 1.64 (td, *J* = 12.0, 5.6 Hz, 2H), 1.60 – 1.53 (m, 1H), 1.49 (d, *J* = 6.9 Hz, 6H), 1.42 – 1.32 (m, 6H), 1.27 – 1.21 (m, 2H), 1.20 – 1.13 (m, 2H), 1.09 – 1.01 (m, 2H), 0.90 – 0.79 (m, 4H), 0.76 – 0.66 (m, 2H), 0.61 – 0.53 (m, 2H). **^{13}C NMR (151 MHz, acetone-D$_6$)** δ 182.18, 165.37, 128.94, 127.16, 126.77, 122.99, 119.28, 113.93, 110.97, 110.24, 102.29, 94.86, 70.69, 59.06, 47.93, 39.77, 32.32, 30.44, 28.25, 26.36, 25.91, 25.53, 23.02, 22.94. **LC-MS:** calculated for $C_{51}H_{66}N_6O_4$ [M+H$^+$] 827.5224 m/z; found 827.5216 m/z.

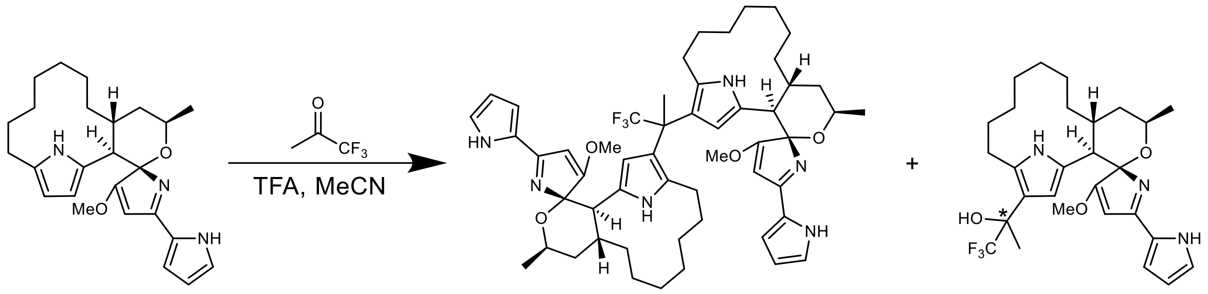

Trifluoromethyl-bridged premarineosin A (6). Amounts: **3** (15.0 mg, 36.8 µmol, 1 equiv), MeCN (1.50 mL), trifluoroacetone (329 µL, 3.68 mmol, 100 equiv), TFA (170 µL, 2.21 mmol, 60 equiv). Reaction time was 4 h. Starting material was recovered (3.0 mg, 7.4 µmol, 20%) as well as two products: dimer **6** (2.0 mg, 2.2 µmol, 6%) and monomer **7** (10 mg, 19 µmol, 52%, dr: 1.0:0.65). If the reaction is run longer (6-8 h), a higher conversion rate to the dimer can be achieved.

The following NMR data corresponds to dimer **6**. Many ^{1}H and ^{13}C signals from distinguishable premarineosin units of the dimer were found to overlap as reported below:

^{1}H NMR (600 MHz, acetone-D$_6$) δ 9.81 (br s, 2H), 8.63 (s, 2H), 7.54 (s, 1H), 7.50 (s, 1H), 7.32 – 7.23 (m, 2H), 6.45 (br t, *J* = 3.0 Hz, 1H), 6.42 (br t, *J* = 3.0 Hz, 1H), 6.13 (s, 1H), 6.09 (s, 1H), 5.71 (br d, *J* = 2.1 Hz, 1H), 5.69 – 5.65 (m, 1H), 4.41 – 4.34 (m, 2H), 4.05 (s, 3H), 4.03 (s, 3H), 2.88 – 2.77 (m, 4H), 2.03 – 1.92 (m, 2H), 1.92 – 1.85 (m, 2H), 1.69 – 1.62 (m, 2H), 1.61 – 1.54 (m, 2H), 1.53 (s, 3H), 1.49 (d, *J* = 7.3 Hz, 3H), 1.48 (d, *J* = 7.3 Hz, 3H), 1.46 – 1.24 (m, 8H), 1.27 – 1.13 (m, 4H), 1.07 – 0.95 (m, 4H), 0.94 – 0.74 (m, 6H), 0.62 – 0.51 (m, 2H). **^{13}C NMR (151 MHz, acetone-D$_6$)** δ 180.95, 168.81, 165.63, 165.58, 134.19, 132.83, 130.72, 129.5 (q, *J* = 320 Hz), 125.84, 123.08, 122.84, 122.28, 121.23, 119.56, 113.65, 113.52, 109.57, 108.09, 97.71, 93.97, 93.92, 71.46, 71.42, 60.48, 60.35, 46.91, 46.84, 44.79 (q, *J* = 25.0 Hz), 38.66, 38.60, 33.56, 27.65, 27.48, 25.90, 25.73, 25.71, 25.50, 25.42, 24.96, 24.75, 24.60, 24.33, 21.23, 21.10. **^{19}F NMR (563 MHz, chloroform-D)** δ -72.5 (s, 3F). **LC-MS:** calculated for $C_{53}H_{67}F_3N_6O_4$ [M+H$^+$] 909.5254 m/z; found 909.5257 m/z.

The following NMR data correspond to both diastereomers of **7**. Many ^{1}H and ^{13}C signals were found to overlap as reported. The CF_3 signal was observed from ^{1}H-^{13}C HMBC analysis and we report as follows: ^{13}C NMR (151 MHz, chloroform-D) 126.1 (q, J ~ 300 Hz). This signal was observed via HMBC analysis and the signal was too broad to distinguish chemical shifts between minor and major diastereomers.

12-trifluoropropanol premarineosin A (7, major diastereomer). ^{1}H NMR (600 MHz, chloroform-D) δ 10.25 (br s, 1H), 7.49 – 7.45 (m, 1H), 7.02 (d, J = 4.0, 1H), 6.40 – 6.37 (m, 1H), 5.70 (s, 1H), 5.54 (s, 1H), 4.45 – 4.39 (m, 1H), 4.01 (s, 3H), 2.93 (d, J = 12.6, 1H), 2.88 – 2.76 (m, 2H), 2.47 – 2.36 (m, 1H), 2.01 – 1.87 (m, 3H), 1.74 – 1.65 (m, 1H), 1.59 (s, 3H), 1.61 – 1.49 (m, 1H), 1.52 (d, J = 6.9 Hz, 3H), 1.45 – 1.38 (m, 2H), 1.35 – 1.22 (m, 3H), 1.17 – 1.09 (m, 1H), 1.02 – 0.82 (m, 2H), 0.60 – 0.49 (m, 1H). **^{13}C NMR (151 MHz, chloroform-D)** δ 179.44, 164.67, 134.32, 132.48, 125.11, 123.58, 121.68, 115.57, 113.16, 109.58, 96.59, 92.83, 73.89 (q, J = 30.0 Hz), 71.60, 59.89, 45.66, 37.92, 31.42, 27.87, 27.32, 26.97, 25.03 – 24.37 (m), 24.05, 20.75. **^{19}F NMR (563 MHz, chloroform-D)** δ -81.3 (s, 3F). **LC-MS:** calculated for $C_{28}H_{36}F_3N_3O_3$ [M+H$^+$] 450.2757 m/z; found 450.2798 m/z.

12-trifluoropropanol premarineosin A (7, minor diastereomer).^{1}H NMR (600 MHz, chloroform-D) δ 10.25 (br s, 1H), 7.49 – 7.45 (m, 1H), 7.01 (d, J = 4.0, 1H), 6.40 – 6.37 (m, 1H), 5.72 (s, 1H), 5.53 (s, 1H), 4.45 – 4.39 (m, 1H), 4.00 (s, 3H), 2.92 (d, J = 12.6, 1H), 2.88 – 2.76 (m, 2H), 2.47 – 2.36 (m, 1H), 2.01 – 1.87 (m, 3H), 1.74 – 1.65 (m, 1H), 1.62 (s, 3H), 1.61 – 1.49 (m, 1H), 1.52 (d, J = 6.9 Hz, 3H), 1.45 – 1.38 (m, 2H), 1.35 – 1.22 (m, 3H), 1.17 – 1.09 (m, 1H), 1.00 – 0.92 (m, 2H), 0.60 – 0.49 (m, 1H). **^{13}C NMR (151 MHz, chloroform-D)** δ 179.43, 164.69, 134.32, 132.13, 125.11, 123.59, 121.65, 116.20, 113.16, 108.88, 96.59, 92.88, 73.92 (q, J = 29.3 Hz), 71.5, 59.9, 45.46, 37.85, 32.11, 27.87, 27.19, 27.09, 25.03 – 24.37 (m), 24.14, 20.79. **^{19}F NMR (563 MHz, acetone-D$_6$)** δ -81.2 (s, 3F). **LC-MS:** calculated for $C_{28}H_{36}F_3N_3O_3$ [M+H$^+$] 450.2757 m/z; found 450.2798 m/z.

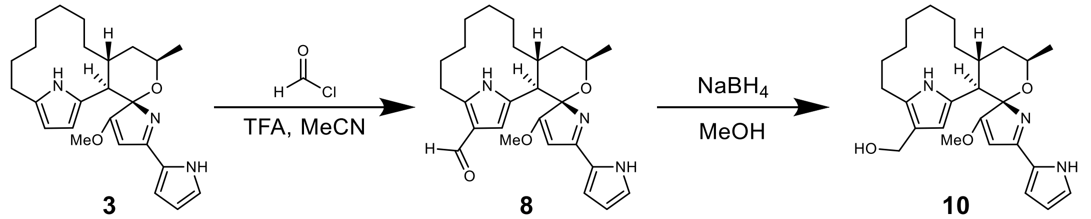

3 8 10

12-formyl premarineosin A (8). Amounts: **3** (31.0 mg, 76.1 µmol), MeCN (3.10 mL), formyl chloride* (327 µL, 6.08 mmol, 80 equiv), TFA (352 µL, 4.56 mmol, 60 equiv). Reaction time was 4 h. Product yield: 32.0 mg (73.5 µmol, 97%). *Formyl chloride was made fresh by using the following procedure: To a flame dried flask and under N_2 thionyl chloride was added (19.0 g, 11.6 mL, 22.8 Eq, 160 mmol). Formic acid (322 mg, 264 µL, 1 Eq, 7.00 mmol) and DMF (132 mg, 140 µL, 0.258 Eq, 1.81 mmol) were added sequentially before the reaction was heated to 70 °C for 1.5 h. There should be a color change from clear to yellow to indicate the reaction is/has progressed. The excess oxalyl chloride was removed by rotoevaporation with KOH solution in the trap. Product obtained should be yellow and was used immediately in next set of reactions. **^{1}H NMR (600 MHz, acetone-D$_6$)** δ 9.67 (s, 1H), 9.32 (br s, 1H), 7.11 - 6.99 (m, 1H), 6.68 – 6.55 (m, 1H), 6.24 – 6.14 (m, 1H), 6.10 (s, 1H), 5.55 (s, 1H), 4.35 – 4.25 (m, 1H), 3.82 (s, 3H), 3.01 (ddd, J = 14.6, 10.5, 3.6 Hz, 1H), 2.77 (d, J = 12.0 Hz, 1H), 2.57 – 2.45 (m, 1H), 2.37 – 2.31 (m, 1H), 1.93 – 1.83 (m, 2H), 1.69 (td, J = 12.5, 5.8 Hz, 1H), 1.62 – 1.57 (m, 1H), 1.57 – 1.50 (m, 1H), 1.51 (d, J = 6.8, 3H), 1.42 – 1.24 (m, 4H), 1.16 – 1.08 (m, 1H), 1.02 – 0.90 (m, 2H), 0.84 – 0.76 (m, 1H), 0.42 – 0.32 (m, 1H). **^{13}C NMR (151 MHz, acetone-D$_6$)** δ 184.48, 181.93, 165.69, 142.84, 131.18, 128.63, 123.48, 122.43, 114.58, 110.60, 108.89, 101.62, 95.00, 70.83, 59.40, 47.48, 39.15, 31.95, 30.02, 27.88, 26.05, 26.01, 25.76, 25.67, 25.42, 25.30, 22.71. **LC-MS:** calculated for $C_{26}H_{33}N_3O_3$ [M+H$^+$] 436.2600 m/z; found 436.2636 m/z.

12-hydroxymethyl premarineosin A (10). To a solution of **8** (19.0 mg, 1 Eq, 43.6 μmol) in MeOH (690 mg, 872 μL, 494 Eq, 21.5 mmol) at 0 °C was added $NaBH_4$ (3.30 mg, 2.00 Eq, 87.2 μmol). The reaction was run until TLC showed consumption of starting material [50% (3% ammonia (7 M in methanol) in DCM solution) in hexanes]. The reaction was quenched by addition of 1M HCl at 0 °C until acidified and extracted with ethyl acetate (x3). The organics combined, dried over sodium sulfate, filtered and concentrated. The crude material was purified by pTLC using 50% (3% ammonia (7 M in methanol) in DCM solution) in hexanes to yield the product **10** (13.0 mg, 29.7 μmol, 68.1 %) as a pale yellow solid. **^{1}H NMR (600 MHz, acetone-D$_6$)** δ 8.48 (br s, 1H), 7.06 – 7.00 (m, 1H), 6.65 – 6.57 (m, 1H), 6.21 – 6.15 (m, 1H), 5.68 (s, 1H), 5.49 (s, 1H), 4.30 – 4.24 (m, 1H), 4.26 (d, *J* = 12.1 Hz, 1H), 4.23 (d, *J* = 12.1 Hz, 1H), (s, 3H), 2.69 (d, *J* = 11.8 Hz, 1H), 2.50 – 2.44 (ddd, *J* = 13.9, 10.6, 3.0 Hz, 1H), 2.16 (dt, *J* = 15.1, 4.6 Hz, 1H), 1.86 – 1.80 (m, 1H), 1.76 – 1.68 (m, 1H), 1.65 (td, *J* = 12.5, 5.9 Hz, 1H), 1.61 – 1.54 (m, 1H), 1.49 (d, *J* = 6.8 Hz, 3H), 1.46 – 1.34 (m, 3H), 1.32 – 1.24 (m, 1H), 1.22 – 1.14 (m, 1H), 1.10 – 1.02 (m, 1H), 0.93 – 0.86 (m, 1H), 0.86 – 0.78 (m, 1H), 0.77 – 0.70 (m, 1H), 0.56 – 0.48 (m, 1H). **^{13}C NMR (151 MHz, acetone-D$_6$)** δ 182.13, 165.50, 129.56, 128.90, 127.67, 123.07, 119.52, 114.05, 110.25, 102.07, 94.84, 70.58, 59.07, 57.33, 47.64, 39.62, 32.22, 30.36, 30.34, 27.94, 26.19, 25.86 – 25.47 (m), 25.39, 22.91. **LC-MS:** calculated for $C_{26}H_{35}N_3O_3$ [M+H$^+$] 438.2757 m/z; found 438.2754 m/z.

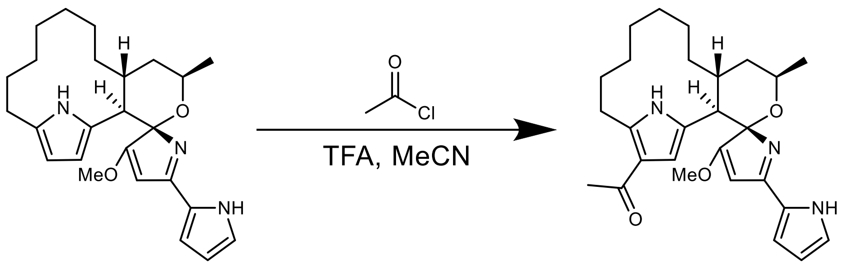

3 **9**

12-acetyl premarineosin A (9). Amounts: **3** (18.0 mg, 44.2 μmol, 1 equiv), MeCN (1.80 mL), acetyl chloride (314 μL, 4.42 mmol, 100 equiv), TFA (2.04 μL, 2.65 mmol, 60 equiv). Reaction time was 4 h. Product yield: 7 mg (16 μmol, 35%). **^{1}H NMR (600 MHz, acetone-D$_6$)** δ 9.42 (br s, 1H), 7.20 – 7.01 (m, 1H), 6.77 – 6.60 (m, 1H), 6.24 – 6.18 (m, 1H), 6.18 (s, 1H), 5.60 (s, 1H), 4.36 – 4.29 (m, 1H), 3.86 (s, 3H), 2.78 (d, *J* = 12.0 Hz, 1H), 2.60 – 2.50 (m, 1H), 2.18 (s, 3H), 1.91 – 1.85 (m, 1H), 1.81 – 1.74 (m, 1H), 1.69 (td, *J* = 12.4, 5.8 Hz, 1H), 1.55 – 1.51 (m, 1H), 1.52 (d, *J* = 6.9 Hz, 3H), 1.52 – 1.44 (m, 1H), 1.44 – 1.39 (m, 1H), 1.37 – 1.33 (m, 1H), 1.31 – 1.24 (m, 2H), 1.18 – 1.10 (m, 1H), 1.03 – 0.93 (m, 2H), 0.80 – 0.70 (m, 1H), 0.40 – 0.30 (m, 1H). **^{13}C NMR (151 MHz, acetone-D$_6$)** 181.79, 165.63, 139.17, 128.03, 124.36, 120.48, 115.5, 111.85, 110.75, 101.30, 94.86, 70.77, 59.38, 47.27, 38.99, 31.74, 30.59, 30.07, 28.49, 28.28, 27.69, 26.06, 25.67, 25.43, 25.03, 22.54. **LC-MS:** calculated for $C_{27}H_{35}N_3O_3$ [M+H$^+$] 450.2757 m/z; found 450.2798 m/z.

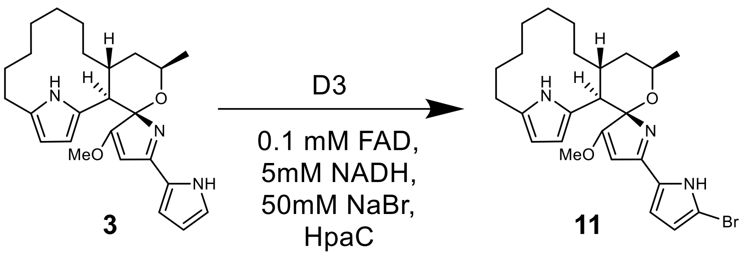

1-bromo premarineosin A (11). Preparatory reactions were performed using 40 μM D3, 5 μM HPAC flavin reductase, 100 μM FAD, 50 mM sodium bromide, and 500 μM of **3** dissolved in DMSO at a final volume of 5mL in reaction buffer (10 mM HEPES, pH 7.4 containing 10% glycerol). The reaction was initiated upon the addition of 5mM NADH. The reaction was quenched via the addition of 20 mL of HPLC-grade methanol, vortexed on the highest setting for 20 s, then water bath sonicated for 30 s. The resulting mixture from all combined 5mL reactions was then passed through a pad of Celite© and the filter cake was washed with an additional 20 mL of methanol. To purify the biocatalytic byproduct, the aqueous solution was extracted with DCM. The organics were combined and concentrated. The resulting material was further purified by preparative HPLC with a phenyl hexyl column (5 μm, 100 Å, 250 x 10 mm) using a gradient of 10-55% of acetonitrile and water, both modified with 0.1% formic acid, over 50 minutes at a flow rate of 5 mL/min. **^{1}H NMR (600 MHz, acetone-D$_6$)** δ 9.49 (br s, 1H), 6.89 (br d, *J* = 2.0 Hz, 1H), 6.17 (br d, *J* = 2.5 Hz, 1H), 5.74 (br s, 1H), 5.68 – 5.65 (m, 1H), 5.52 – 5.48 (m, 1H), 4.33 (br p, *J* = 6.4 Hz, 1H), 3.91 (s, 3H), 2.93 (d, *J* = 12.3 Hz, 1H), 2.66 – 2.58 (m, 1H), 2.46 (dt, *J* = 15.1, 4.6 Hz, 1H), 2.31 (t, *J* = 14.0, 2.6 Hz, 1H), 1.91 (dt, *J* = 13.6, 4.0 Hz, 1H), 1.80 (br q, *J* = 11 Hz, 1H), 1.70 – 1.63 (m, 2H), 1.50 (d, *J* = 6.7 Hz, 1H), 1.48 – 1.43 (m, 1H), 1.43 – 1.30 (m, 3H), 1.21 – 1.13 (m, 1H), 1.09 – 1.00 (m, 2H), 0.84 – 0.75 (m, 2H), 0.61 – 0.50 (m, 1H).**^{13}C NMR (151 MHz, acetone-D$_6$)** δ 177.65, 161.77, 133.17, 130.58, 127.36, 124.88, 117.10, 109.87, 105.89, 97.07, 93.38, 70.46, 59.42, 46.91, 38.94, 33.30, 29.01, 28.80, 27.38, 25.84, 25.66, 25.28, 22.06. **LC-MS:** calculated for $C_{25}H_{32}BrN_3O_2$ [M+H$^+$] 486.1756 m/z; found 486.1771 m/z.

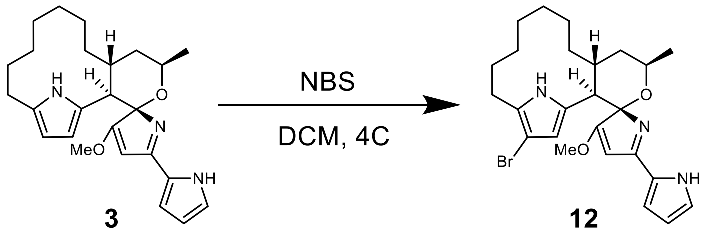

12-bromo premarineosin A (12). To a solution of **3** (13.8mg, 5mmol) in dichloromethane at 4 °C was added *N*-Bromo succinimide (10 mg, 10mmol, DCM) drop wise. The mixture was stirred at 4 °C. After 2 h the reaction was quenched with sodium bicarbonate. Following a liquid-liquid extraction with DCM, the organic layer was extracted and dried. The resulting material was further purified by preparative HPLC with a phenyl hexyl column (5 μm, 100 Å, 250 x 10 mm) using a gradient of 10-55% of acetonitrile and water, both modified with 0.1% formic acid, over 50 min at a flow rate of 5 mL/min. **^{1}H NMR (600 MHz, chloroform-D, formate adduct)** δ 14.68 (br s, 1H), 10.56 (br s, 1H), 13.41 (br s, 1H), 8.84 (s, 1H), 7.48 (br s, 1H), 7.07 (br d, *J* = 2.90 Hz, 1H), 5.77 (br s, 1H), 5.58 (br s, 1H), 4.41 (br p, *J* = 6.8 Hz, 1H), 4.05 (s, 3H), 2.93 (d, *J* = 12.6 Hz, 1H), 2.80 – 2.70 (m, 1H), 2.49 (ddd, *J* = 14.7, 10.9, 3.6 Hz, 1H), 2.32 (dt, *J* = 14.7, 4.6 Hz, 1H), 1.92 (dt, *J* = 13.8, 4.0 Hz, 1H), 1.72 – 1.64 (m, 1H), 1.52 (d, *J* = 6.9 Hz, 3H), 1.42 – 1.32 (m, 3H), 1.30 – 1.22 (m, 1H), 1.17 – 1.09 (m, 1H), 1.05 – 0.97 (m, 1H), 0.96 – 0.83 (m, 2H), 0.53 – 0.44 (m, 1H). **^{13}C NMR (151 MHz, chloroform-D)** δ 179.34, 170.28, 164.40, 134.62, 132.06, 125.88, 124.67, 121.12, 113.53, 111.85, 96.27, 92.75, 71.37, 60.03, 45.48, 37.74, 32.37, 27.36, 27.18, 26.31, 25.17, 24.47, 24.33, 20.87. **LC-MS:** calculated for $C_{25}H_{32}BrN_3O_2$ [M+H$^+$] 486.1756 m/z; found 486.1811 m/z.

Supplementary Tables.

Supplementary Table 1. (−)-Premarineosin A (**3**) biosynthetic genes in *S. eitanensis* and their homology to the *Streptomyces* sp. CNQ-617 *mar* BGC genes.

S. eitanensis Protein	Description	*S.* sp. CNQ-617 NCBI Accession No.	Similarity (%)	Identity (%)
PmaD	transcriptional regulator, SARP family	AHF22840.1	77%	65%
PmaX	beta-ketoacyl synthase	AHF22841.1	68%	60%
PmaW	acyl-CoA dehydrogenase	AHF22842.1	83%	74%
PmaY	unknown	AHF22843.1	68%	57%
PmaZ	LuxR family DNA binding response regulator	AHF22844.1	74%	55%
PmaV	unknown	AHF22845.1	64%	56%
PmaU	4’-phosphopantetheinyl transferase	AHF22846.1	69%	58%
PmaT	unknown	AHF22847.1	76%	63%
PmaR	Beta-ketoacyl synthase	AHF22848.1	86%	79%
PmaQ	PP-binding	AHF22849.1	82%	67%
PmaP	3-oxoacyl-(acyl carrier protein) synthase III	AHF22850.1	84%	76%
PmaO	Putative acyl carrier protein	AHF22851.1	85%	69%
PmaN	PP-binding, aminotransferase I/II, Amino-7-oxononanoate synthase	AHF22852.1	77%	68%
PmaM	AMP-binding, AMP-dependent synthetase and ligase	AHF22853.1	76%	67%
PmaL	NRPS-PKS hybrid	AHF22854.1	70%	61%
PmaK	RmlD_sub_binding, NAD-dependent epimerase/dehydratase	AHF22855.1	81%	73%
PmaJ	thioesterase	AHF22856.1	64%	56%
PmaI	methyltransferase	AHF22857.1	81%	70%
PmaH	PPDK_N	AHF22858.1	83%	75%
PmaG	NONE	AHF22859.1	85%	77%

Supplementary Table 2. Strains used in this work.

Strain	Genotype	Reference
***Streptomyces* Strains**		
WT	*Streptomyces eitanensis* wild-type strain	Sherman Lab Collection[8]
FP10016	*Streptomyces eitanensis*::*kas*Op*, Kan^R	This Work
FP10017	*Streptomyces eitanensis*::*kas*Op*-*pmaD*, Kan^R	This Work
FP10018	*Streptomyces eitanensis*::*kas*Op*-*pmaD-pmaG,* Kan^R	This Work
	Streptomyces sp. *CNQ-617*	Prof. William Fenical[5]
***E. coli* Strains**		
DH5α	*fhuA2Δ(argF-lacZ)U169 phoA glnV44 Φ80Δ(lacZ)M15 gyrA96 recA1 relA1 endA1 thi-1 hsdR17*	New England Biolabs
S17-1	RP4 derivative integrated in chromosome	Laboratory Stock

Supplementary Table 3. Plasmids used in this work.

Plasmid	Relevant Characteristics	Reference
pSET152	Integrative *E. coli-Streptomyces* shuttle vector, attP (ΦC31 integrase), Apr^R	Laboratory Stock[7]
pSET152k	Derived from pSET152, Kan^R	Laboratory Stock[8]
pSET152k-*kasOp**	Derived from pSET152k, expression driven by *kasO** promoter, Kan^R	Laboratory Stock[9]
pSET152k-*pmaD*	Derived from pSET152k-*kas*Op*, *expressing pmaD*, Kan^R	This Work
pSET152k-*pmaDG*	Derived from pSET152k-*kas*Op*, expressing *pmaD and pmaG*, Kan^R	This Work
pET28b(D3)	Derived from pET28b, expressing flavin-dependent halogenase D3, Amp^R	Laboratory Stock[10]
pET21a-HpaC (phaC)	Derived from pSET21a, expressing the flavin reductase HpaC, Amp^R	Laboratory Stock[11,12]

Supplementary Table 4. Primers used in this work.

Plasmid	Name	Sequence
pSET152k-*pmaD*	pmaD_fwd	TCTAAGTAAGGAGTGTCCATATGGCTATCCATCTTCTC
	pmaD_rev	GACGACAAAACTTTAGAGATTCACACGAGGCCGGCGTGCG
pSET152k-pmaDG	pmaD_fwd	see above
	pmaD-pmaG_rev	ATGGACACTCCTTACTCGAATCACACGAGGCCGGCGTGCG
	pmaD-pmaG_fwd	TTCGAGTAAGGAGTGTCCATATGATCCCCGACCTGTGG
	pmaG_rev	AGACGACAAAACTTTAGATCACCCTGCCGCCGCAC

Supplementary Table 5. Gene sequences used in this work.

Gene	Sequence
pmaD	Atggctatccatcttctcggtccgatatccgtgttcacggacggccgcccgcacggtgtgggatccagcaaagtccgga ccatgctcgccgtcctcgccctggaagcaggccaggcggtttcccatacggatctggcggacgaactctggtcgggcg acaccgtccgcaatccccgcaacgccatgcaggcacatgccacccgggtgcgcagggtgctgaagggcgagggc gggcggtgcggctcggggaccaccgcgctgcgctccgtacccaacggctacgtcctggacgtcccgcgcgagcac atcgacgggcaccggtttctcgacctggcgtcccaggcggcgacaacgctggccgacgcccccgaccgggcacttc gtctgctggactccgcgctgctcctgtggcgtggcccggccctgctggacgcgggcgacgggctgcgctgccgcagc gcggcccggctgttcgaggaaagacgtctctcagccctggcggaccaggtgcgggctcgcatcatgctcggagatca cgctcgggcgatcgccgaactgcaccggctggtggcccagttcccgctgcgggaacatttctgtgatctgctcatgctg gcgttgtaccaggtcgggcgccagagcgacgccctggagcagttccggctggcccggcagcgtctcgacgaggaa ctgggcatcaggcccggggaactgctccagcgccggcacgccgagatcctggcgcaggatcccgcgttggcgcac gccggcctcgtgtga
pmaG	atgatccccgacctgtggtatccgatcgccatatccacggatgtcaacacgaagaagcccacgagcctgcgccgcct cggcatgcggctggtgctgtggcgggacatggacggcaacgtcgtcgtccaggacgcacggtgcccgcacaaggg ggccgacctgggtgacggccgtctgaagggcaacgcggtggagtgcccgtaccacggcttcctgttcaaccccgag ggcacgtgcacgctgaccccggcgatgggcaggaacgcccgtatacccggctcgctcaagatccgttcgtacccggt gcaggagcgtcatggcctgatctggatgtggtggggtgacgaccgcccggccgaggagcgtccggacatcatcgcg ccccgcgagatcaaggagcacgagggcctctactccacgatctcgtgggagaagccggtccactacacgcggtac atcgagagcgtccaggagttctaccacgtcacctatgtgcaccgggatcactggctgaactacctggacttcaacttcct ttacgggacgctgcgcaagttcggcctggacggaaagcagaacttcctcaaggccaccatgatccacaaccatcatg tggagacggacgacgacggaatgacgttccggtactcgttcgaccacggccaggaggacgacccgagcaagagc atccactacgtcatcacgttctcgttcccctgcatggtgcacgtgcagaccgaggcgttcgagacgacgtcctggttcat gccggtggacgaggaacacacccagcacatcttccgcgtctacgagttcccgcagctgaagccgtacctgcgactgc cgtcgctgcggcgcctgatgtccacgctccaggtgtacctggagaagttcgtccaggacccgcaggacttcaagatca tggcgaagcaggagccccggatcagcggcggcggcgtcaacaagttcatcccggtggacgagatgaacgccaag ttcatcaagacccgcgcccggctgctgcgggaggccggggagcgccgcgaggaaacggtccacgacacccacga cggcatggtcggcgggatggacatggccgaggacctggacgtgcgggacgcgccttcccccgccaacggcaacg gccactccgcccccaccgtgcccccgagcgacgaggtcgagacctggtccggtgcggcggcagggtga

Supplementary Figures.

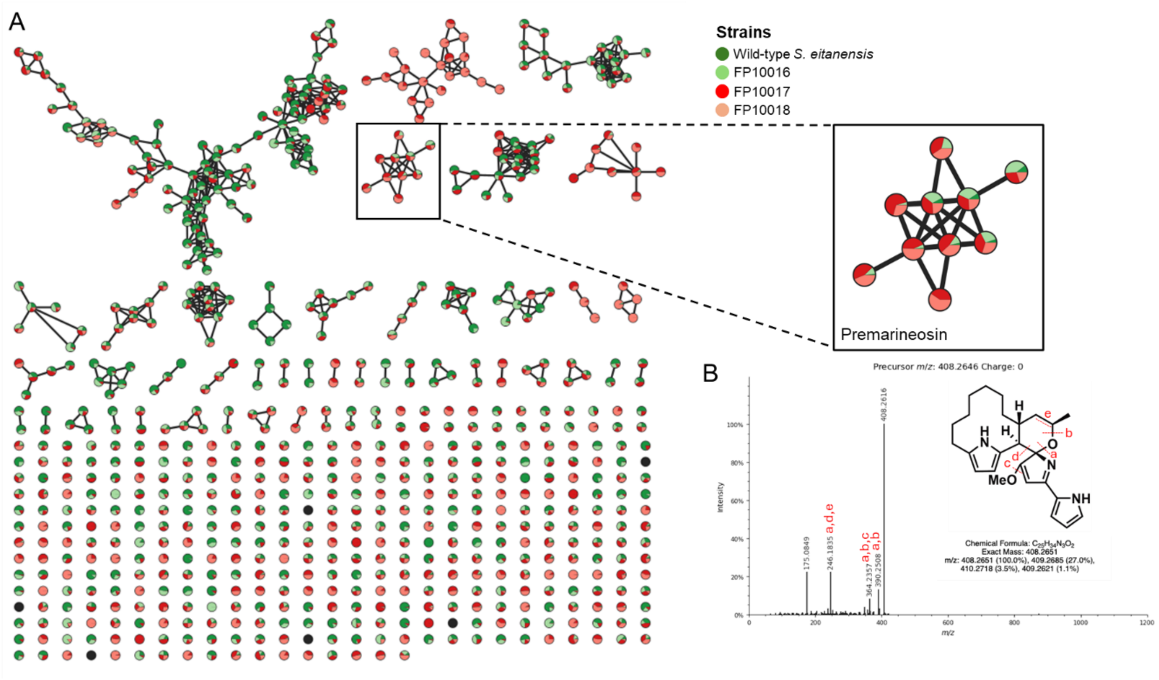

Supplementary Figure 1. Untargeted metabolic analysis of *S. eitanensis* revealed production of a compound with the same molecular weight and fragmentation pattern as premarineosin A (3). (**A**) Feature based analysis of untargeted metabolomics data from wild-type, vector control strain FP10016, and engineered strains FP10017 and FP10018 at day seven of strain cultivation in GICYE. (**B**) MS/MS fragmentation spectra for premarineosin A (**3**) (408.26 m/z) with noted major fragments.

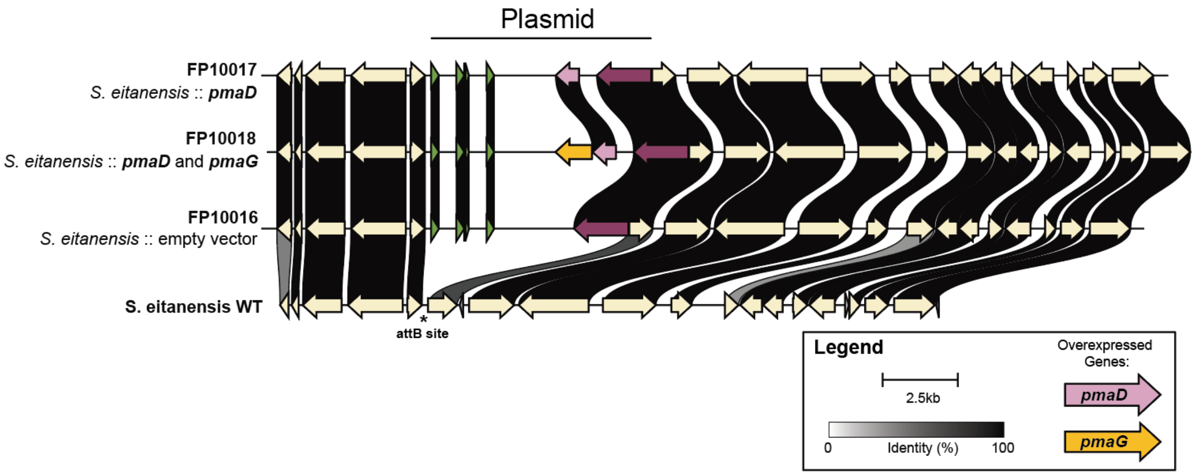

Supplementary Figure 2. Integration of plasmids containing *pmaD* and *pmaG* in the *Streptomyces eitanensis* genome. Comparative analysis of wild-type, FP10016, FP10017, and FP10018 genomes at the *attB* integration site using clinker[13].

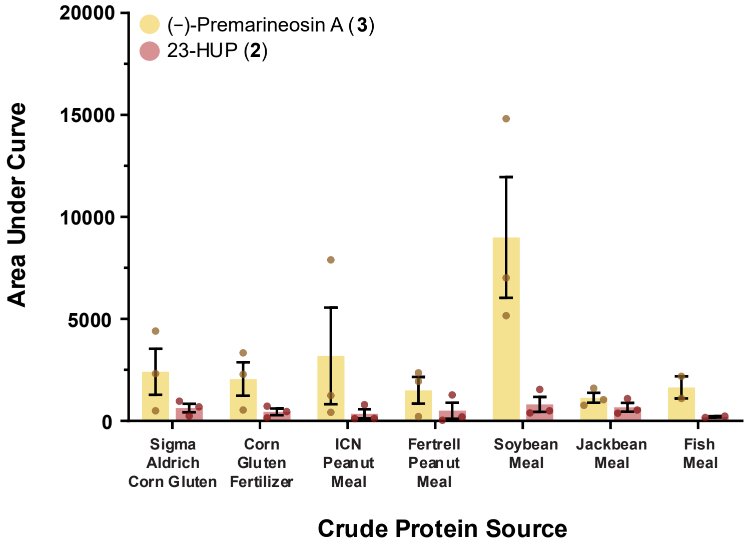

Supplementary Figure 3. Effect of crude complex protein source on yield of (−)-premarineosin A (3) in 1 mL extracts from 50 mL cultures of FP10018. Area Under Curve was calculated in GraphPad Prism version 10.4.2. Dots represent three independent culture replicates. Error bars represent standard error (n=3).

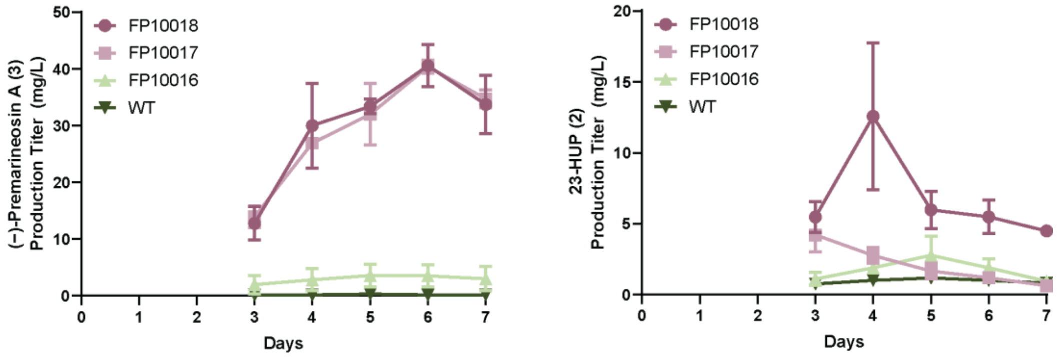

Supplementary Figure 4. (−)-Premarineosin A (3) and 23-HUP (2) production (mg/L) increase over time. Titers quantified by AUC (HPLC) against standard curves of each compound. Error bars indicate standard deviation (n=3). Production titers were below the limit of detection on days 1 and 2.

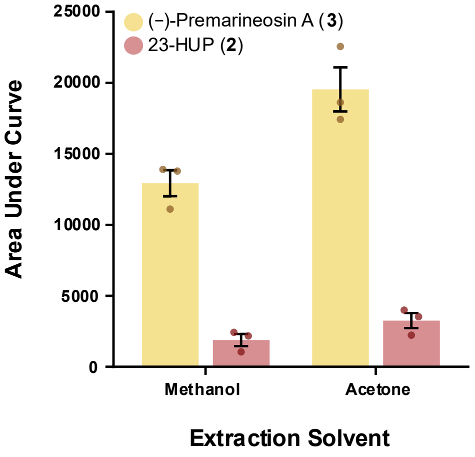

Supplementary Figure 5. Extracting with acetone improves (−)-premarineosin A (3) titers. Production quantified by Area Under Curve (HPLC) from 1 mL extracts of FP10018 cultures grown in soybean meal culture media. Area Under Curve was calculated in GraphPad Prism version 10.4.2. Dots represent three independent culture replicates. Error bars represent standard error (n=3).

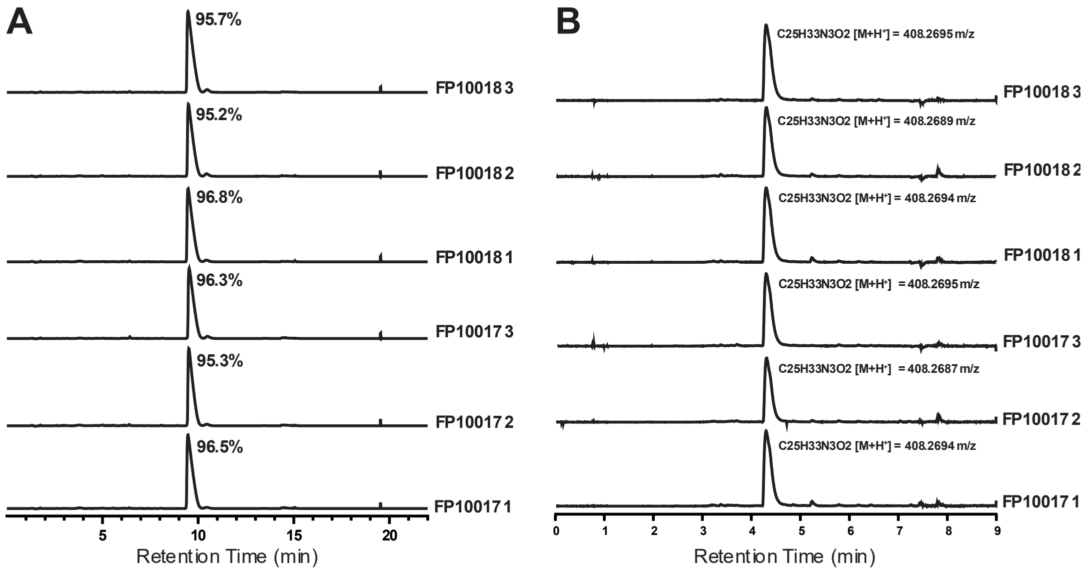

Supplementary Figure 6. Purity analysis of replicate isolated yields of (−)-premarineosin A (3). (A) HPLC traces for each replicate; **3** elutes at 9.5 min. The percentage represents degree of purity based on AUC at 346nm. **(B)** ESI EIC MS traces for each replicate; **3** elutes at 4.4 min.

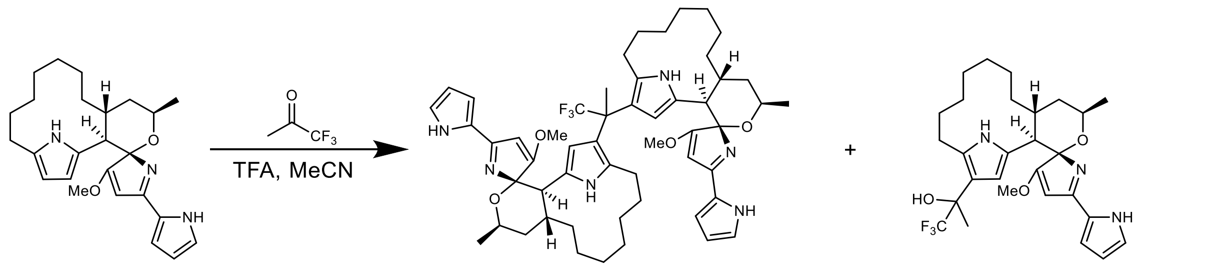

(-)-Premarineosin A (3)

Trifluoromethyl-bridged premarineosin A (6)
(6%)

12-trifluoropropanol premarineosin A (7)
(56%)

Supplementary Figure 7. Formation of the 12-trifluoropropanol premarineosin A (7) monomer.

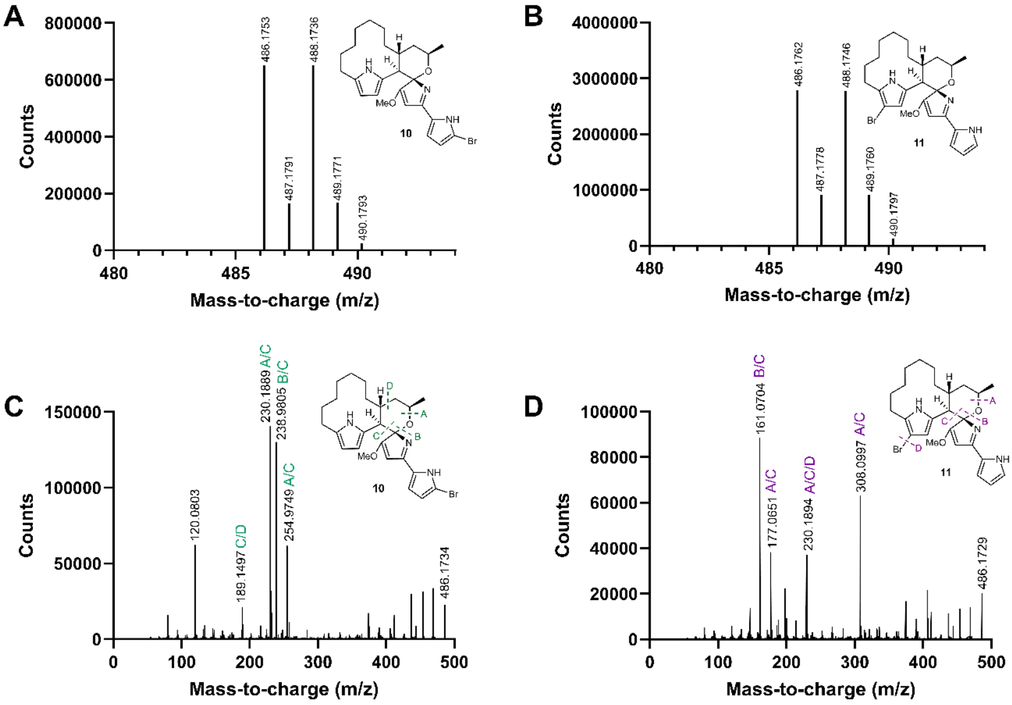

Supplementary Figure 8. LC-MS/MS characterization of 1-bromo (11) and 12-bromo (12) premarineosin A. Isotope pattern of **11** (A) and **12** (B), and MS2 fragmentation patterns for **11** (C) and **12** (D).

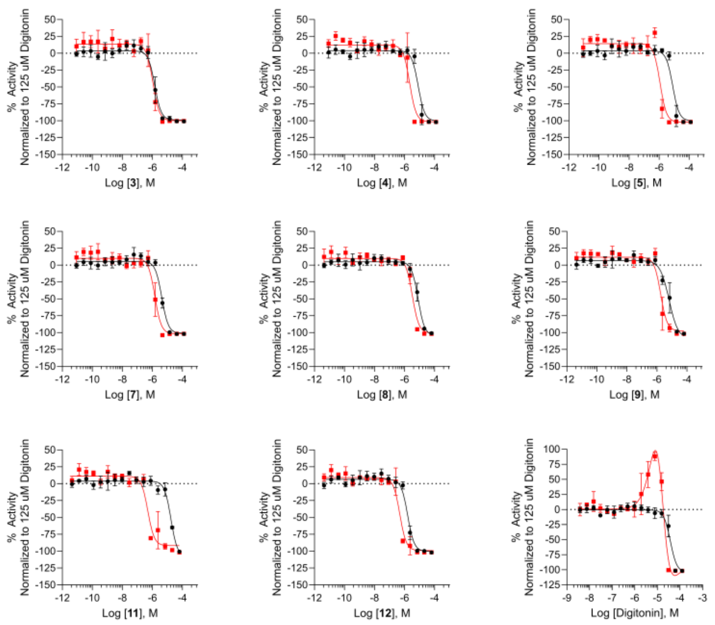

Supplementary Figure 9. Mammalian cell cytotoxicity (72 h treatment) in HEK293 embryonic kidney (black symbols) and MOLT4 T-cell leukemia (red symbols) cell lines for four replicates per cell line (open and closed symbols). Data was normalized to 125 μM Digitonin as -100% inhibition. Error bars represent standard deviation (n=4).

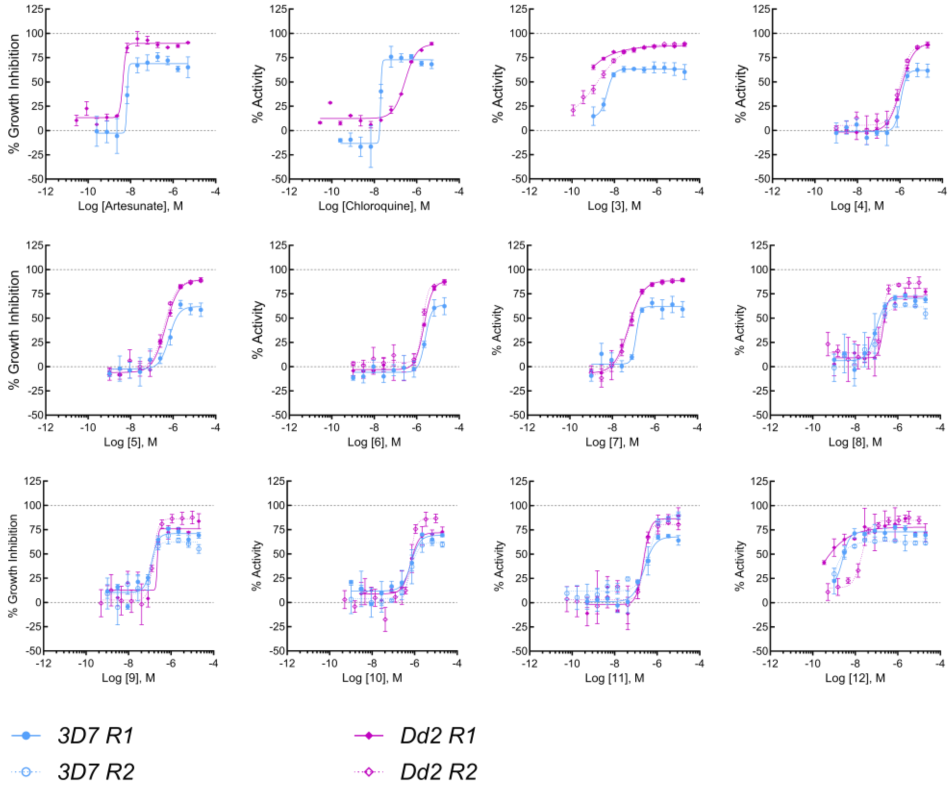

Supplementary Figure 10. Activity of artesunate, chloroquine, (−)-premarineosin A (3), and derivatives 4-12 against *P. falciparum* 3D7 (chloroquine-sensitive) and Dd2 (chloroquine-resistant) parasite lines in 48 h assays. Each *in vitro* experiment was performed in triplicate wells. Chloroquine and artesunate were tested in a single replicate experiment for each strain. Compounds **3-7** were tested in two independent experiments in Dd2 and one experiment in 3D7, and compounds **8-12** were tested in two independent experiments against both Dd2 and 3D7. Error bars represent standard deviation (n=3).

Supplementary Notes.

Supplementary Note 1. X-Ray Crystal Structure Determination for (−)-premarineosin A.

Experimental. Single colourless prism-shaped crystals of **2** were used as supplied. A suitable crystal with dimensions 0.21 × 0.11 × 0.09 mm^3 was selected and mounted on a dtrek-CrysAlisPro-abstract goniometer imported rigaku-d*trek images diffractometer. The crystal was kept at a steady *T* = 85 K during data collection. The structure was solved with the ShelXT 2018/2[14] solution program using dual methods and by using Olex2 1.5-alpha[15] as the graphical interface. The model was refined with ShelXL 2019/3[16] using full matrix least squares minimisation on F^2.

Crystal Data. $C_{25}H_{35}N_3O_3$, M_r = 425.56, orthorhombic, $P2_12_12_1$ (No. 19), a = 8.14850(10) Å, b = 12.6238(2) Å, c = 21.7326(3) Å, *a* = *b* = *g* = 90°, *V* = 2235.53(5) Å^3, *T* = 85 K, *Z* = 4, *Z'* = 1, *m*(Cu K$_a$) = 0.662, 33092 reflections measured, 4146 unique (R_{int} = 0.0795) which were used in all calculations. The final wR_2 was 0.0971 (all data) and R_1 was 0.0373 (I≥2 *s*(I)).

Formula	$C_{25}H_{35}N_3O_3$
$D_{calc.}$/ g cm^{-3}	1.264
m/mm^{-1}	0.662
Formula Weight	425.56
Colour	colourless
Shape	prism-shaped
Size/mm^3	0.21×0.11×0.09
T/K	85
Crystal System	orthorhombic
Flack Parameter	0.09(13)
Hooft Parameter	0.13(10)
Space Group	$P2_12_12_1$
a/Å	8.14850(10)
b/Å	12.6238(2)
c/Å	21.7326(3)
a/°	90
b/°	90
g/°	90
V/Å^3	2235.53(5)
Z	4
Z'	1
Wavelength/Å	1.54184
Radiation type	Cu K$_a$
Q_{min}/°	4.050
Q_{max}/°	69.810
Measured Refl's.	33092
Indep't Refl's	4146
Refl's I≥2 *s*(I)	3937
R_{int}	0.0795
Parameters	318
Restraints	12
Largest Peak	0.204
Deepest Hole	-0.193
GooF	1.088
wR_2 (all data)	0.0971
wR_2	0.0888
R_1 (all data)	0.0418
R_1	0.0373

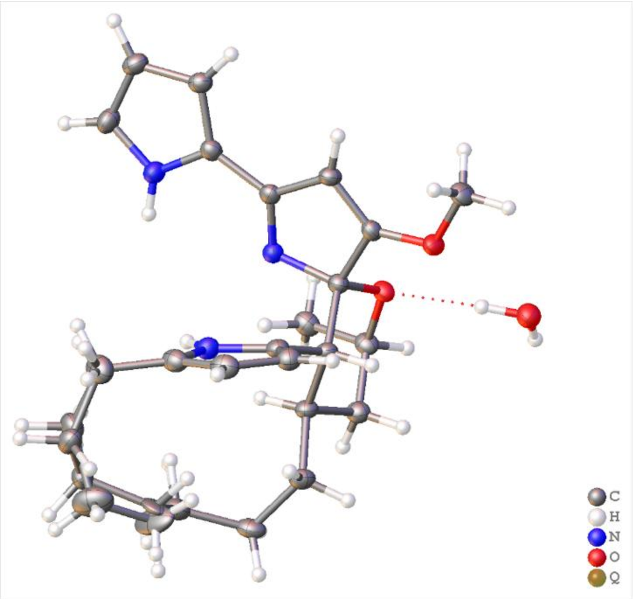

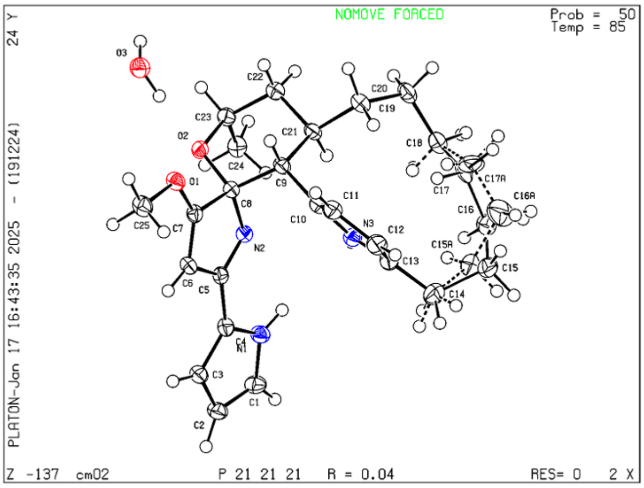

Structure Quality Indicators

**Reflections:**	d min (CuKα) 2Θ=139.6° 0.82	I/σ(I) 27.8	Rint m=8.01 7.95%	Full 135.4° 99% to 139.6° 100	
**Refinement:**	Shift -0.001	Max Peak 0.2	Min Peak -0.2	GooF 1.088	Hooft .13(10)

A colorless prism-shaped-shaped crystal with dimensions 0.21 × 0.11 × 0.09 mm^3 was mounted. Data were collected using a dtrek-CrysAlisPro-abstract goniometer imported rigaku-d*trek images diffractometer operating at T = 85 K.

Data were measured using *w* scans with Cu K_a radiation. The diffraction pattern was indexed and the total number of runs and images was based on the strategy calculation from the program DTREK_VERSION=d*TREK version 9.9.9.4 W9RSSI – Aug 22 2012. The maximum resolution that was achieved was Q = 69.810° (0.82 Å).

The unit cell was refined using CrysAlisPro 1.171.43.130a[17] on 18942 reflections, 57% of the observed reflections.

Data reduction, scaling and absorption corrections were performed using CrysAlisPro 1.171.43.130a[17]. The final completeness is 100.00 % out to 69.810° in Q. A multi-scan absorption correction was performed using CrysAlisPro 1.171.43.130a[17]. Empirical absorption correction using spherical harmonics, implemented in SCALE3 ABSPACK scaling algorithm.. The absorption coefficient *m* of this material is 0.662 mm^{-1} at this wavelength (l = 1.54184Å) and the minimum and maximum transmissions are 0.814 and 1.000.

The structure was solved and the space group $P2_12_12_1$ (# 19) determined by the ShelXT 2018/2[14] structure solution program using dual methods and refined by full matrix least squares minimization on $\boldsymbol{F^2}$ using version 2019/3 of ShelXL 2019/3[16]. All non-hydrogen atoms were refined anisotropically. Hydrogen atom positions were calculated geometrically and refined using the riding model. Most hydrogen atom positions were calculated geometrically and refined using the riding model, but some hydrogen atoms were refined freely.

_exptl_absorpt_process_details: CrysAlisPro 1.171.43.130a[17] using spherical harmonics, implemented in SCALE3 ABSPACK scaling algorithm.

There is a single formula unit in the asymmetric unit, which is represented by the reported sum formula. In other words: Z is 4 and Z' is 1. The moiety formula is C25 H33 N3 O2, H2 O.

The Flack parameter was refined to 0.09(13). Determination of absolute structure using Bayesian statistics on Bijvoet differences using the Olex2 results in 0.13(10). The chiral atoms in this structure are: C8(S), C9(R), C21(R), C23(R). Note: The Flack parameter is used to determine chirality of the crystal studied, the value should be near 0, a value of 1 means that the stereochemistry is wrong and the model should be inverted. A value of 0.5 means that the crystal consists of a racemic mixture of the two enantiomers.

Fractional Atomic Coordinates (×10[4]) and Equivalent Isotropic Displacement Parameters ($Å^2$×10[3]) for **2**. U_{eq} is defined as 1/3 of the trace of the orthogonalised U_{ij}.

Atom	x	y	z	U_{eq}
O1	7086(2)	6707.0(14)	4084.1(8)	23.2(4)
O2	5883(2)	5983.1(13)	2941.7(8)	20.6(4)
O3	9377(3)	5605.3(15)	3034.9(9)	28.6(4)
N1	364(3)	7831.4(17)	3170.5(10)	23.4(5)
N2	3222(3)	6581.2(16)	3272.2(9)	19.2(4)
N3	1647(3)	5099.1(17)	4234.5(10)	22.0(5)
C1	-745(3)	8630(2)	3131.9(12)	26.5(6)
C2	-99(3)	9511(2)	3418.6(12)	25.9(6)
C3	1470(3)	9223(2)	3642.3(12)	22.8(5)
C4	1732(3)	8176(2)	3478.5(11)	20.4(5)
C5	3151(3)	7493.0(19)	3548.7(11)	19.1(5)
C6	4598(3)	7767.2(19)	3920.4(11)	20.3(5)
C7	5599(3)	6930.4(19)	3861.4(11)	19.3(5)
C8	4792(3)	6104.9(19)	3453.3(11)	19.1(5)
C9	4516(3)	5044.9(19)	3791.7(11)	20.0(5)
C10	3327(3)	5099.9(19)	4318.9(12)	21.4(5)
C11	3605(3)	4934(2)	4933.4(12)	24.5(6)
C12	2053(4)	4815(2)	5224.1(12)	28.2(6)
C13	861(4)	4910(2)	4786.5(12)	26.3(6)
C14	-953(4)	4776(2)	4830.7(14)	35.2(7)
C15	-1675(6)	3660(3)	4799(2)	30.3(13)
C15A	-1627(9)	3888(5)	4417(4)	31(2)
C16	-1368(6)	3094(4)	4183(2)	31.1(14)
C16A	-1119(15)	2716(6)	4448(5)	50(3)
C17	380(8)	2633(9)	4142(7)	35(2)
C17A	370(20)	2421(19)	4142(13)	55(5)
C18	898(4)	2604(2)	3475.3(14)	32.1(7)
C19	2687(4)	2303(2)	3372.3(13)	27.5(6)
C20	3924(3)	3071(2)	3661.4(12)	24.9(6)
C21	4030(3)	4155.1(19)	3336.7(12)	20.6(5)
C22	5272(3)	4107(2)	2808.6(12)	23.9(6)
C23	5477(3)	5171.4(19)	2488.9(11)	22.2(5)
C24	4038(3)	5481(2)	2082.0(12)	25.8(6)
C25	7782(3)	7494(2)	4488.7(13)	27.0(6)

Anisotropic Displacement Parameters (×10[4]) for **2**. The anisotropic displacement factor exponent takes the form: $-2p^2[h^2a^{*2} \times U_{11}+ ... +2hka^* \times b^* \times U_{12}]$

Atom	U_{11}	U_{22}	U_{33}	U_{23}	U_{13}	U_{12}
O1	20.0(9)	19.4(8)	30.3(9)	-2.9(7)	-5.9(8)	-0.7(7)
O2	20.6(9)	19.4(9)	21.8(8)	-3.5(7)	2.3(7)	-3.0(7)
O3	25.0(10)	25.7(10)	35.0(10)	-3.3(8)	-1.1(9)	-1.0(8)
N1	21.3(11)	20.5(10)	28.5(11)	-1.3(9)	-2.5(9)	2.2(9)
N2	18.4(10)	17.6(10)	21.6(10)	0.7(8)	0.2(8)	0.0(8)
N3	22.4(11)	21.0(10)	22.6(10)	0.6(9)	0.6(9)	1.0(9)
C1	22.8(13)	26.8(14)	29.8(14)	0.6(11)	-2.4(11)	5.9(11)
C2	28.5(14)	20.1(12)	28.9(13)	1.7(11)	2.3(11)	5.0(11)
C3	25.5(14)	18.1(12)	24.8(12)	1.0(10)	1.1(11)	0.6(10)
C4	21.2(12)	19.0(12)	21.1(12)	2.3(10)	-0.5(10)	-1.9(10)
C5	19.0(12)	17.5(12)	20.8(12)	2.6(9)	1.3(10)	-1.4(10)
C6	22.9(13)	15.2(11)	22.7(12)	-0.6(10)	-1.1(10)	-1.1(10)
C7	18.2(12)	18.5(12)	21.3(11)	0.7(10)	-1.3(10)	-2.6(10)
C8	19.1(12)	16.8(11)	21.5(12)	-1.1(9)	1.6(10)	-0.8(9)
C9	22.0(13)	15.1(11)	23.0(12)	1.1(10)	-0.8(10)	0.7(10)
C10	22.8(13)	14.7(11)	26.6(12)	-1.5(10)	0.8(11)	1.1(10)

Atom	U_{11}	U_{22}	U_{33}	U_{23}	U_{13}	U_{12}
C11	26.6(14)	22.2(13)	24.6(13)	-1.6(11)	-0.1(10)	0.2(11)
C12	35.9(16)	25.8(14)	23.0(12)	-1.5(11)	6.3(11)	-0.8(12)
C13	29.1(14)	21.9(13)	27.8(13)	-2.3(11)	7.4(11)	-1.4(11)
C14	32.4(16)	35.4(17)	37.7(16)	-5.5(13)	11.2(13)	-3.2(13)
C15	25(2)	28(2)	38(3)	7(2)	5(2)	3(2)
C15A	23(4)	31(5)	39(5)	-1(4)	5(4)	-5(3)
C16	21(2)	25(3)	47(3)	-3(3)	-5(2)	-3(2)
C16A	73(8)	37(6)	40(6)	11(4)	15(5)	4(5)
C17	37(4)	15(4)	54(4)	-12(3)	1(3)	-13(3)
C17A	66(8)	33(9)	67(7)	0(7)	47(7)	13(6)
C18	32.7(16)	20.2(13)	43.3(17)	0.6(12)	1.3(13)	-4.1(12)
C19	37.8(16)	17.1(12)	27.7(13)	-2.9(11)	3.1(12)	-4.5(11)
C20	29.5(14)	18.3(12)	27.1(13)	0.9(10)	-1.5(11)	0.7(11)
C21	23.2(13)	15.1(12)	23.5(12)	-1.7(10)	0.0(11)	0.6(10)
C22	25.4(14)	20.3(12)	26.0(13)	-1.4(10)	2.6(11)	0.0(10)
C23	24.7(14)	21.1(12)	20.7(11)	-5.1(10)	2.2(10)	-2.5(11)
C24	28.5(14)	26.6(13)	22.3(12)	-0.5(11)	0.2(11)	-4.3(11)
C25	26.9(15)	24.4(13)	29.6(14)	-5.1(11)	-7.0(11)	-3.9(11)

Bond Lengths in Å for **2.**

Atom	Atom	Length/Å
O1	C7	1.335(3)
O1	C25	1.443(3)
O2	C8	1.432(3)
O2	C23	1.459(3)
N1	C1	1.357(3)
N1	C4	1.372(3)
N2	C5	1.300(3)
N2	C8	1.467(3)
N3	C10	1.382(3)
N3	C13	1.381(3)
C1	C2	1.379(4)
C2	C3	1.416(4)
C3	C4	1.385(4)
C4	C5	1.450(3)
C5	C6	1.471(3)
C6	C7	1.341(4)
C7	C8	1.518(3)
C8	C9	1.543(3)
C9	C10	1.502(3)

Atom	Atom	Length/Å
C9	C21	1.548(3)
C10	C11	1.371(4)
C11	C12	1.421(4)
C12	C13	1.365(4)
C13	C14	1.491(4)
C14	C15	1.528(3)
C14	C15A	1.538(3)
C15	C16	1.537(3)
C15A	C16A	1.538(3)
C16	C17	1.541(3)
C16A	C17A	1.430(17)
C17	C18	1.509(16)
C17A	C18	1.53(3)
C18	C19	1.523(4)
C19	C20	1.533(4)
C20	C21	1.542(3)
C21	C22	1.531(4)
C22	C23	1.522(3)
C23	C24	1.520(4)

Bond Angles in ° for **2**.

Atom	Atom	Atom	Angle/°
C7	O1	C25	115.6(2)
C8	O2	C23	117.28(18)
C1	N1	C4	109.6(2)
C5	N2	C8	106.1(2)
C13	N3	C10	110.1(2)
N1	C1	C2	108.5(2)
C1	C2	C3	107.0(2)
C4	C3	C2	107.2(2)
N1	C4	C3	107.6(2)
N1	C4	C5	120.7(2)
C3	C4	C5	131.5(2)
N2	C5	C4	120.9(2)
N2	C5	C6	115.3(2)
C4	C5	C6	123.8(2)
C7	C6	C5	104.5(2)

Atom	Atom	Atom	Angle/°
N3	C10	C9	122.5(2)
C11	C10	N3	107.0(2)
C11	C10	C9	129.1(2)
C10	C11	C12	107.6(2)
C13	C12	C11	108.3(2)
N3	C13	C14	122.4(3)
C12	C13	N3	106.9(2)
C12	C13	C14	130.6(3)
C13	C14	C15	118.9(3)
C13	C14	C15A	113.5(4)
C14	C15	C16	113.9(4)
C14	C15A	C16A	125.4(7)
C15	C16	C17	112.2(6)
C17A	C16A	C15A	117.2(13)
C18	C17	C16	108.9(8)

Atom	Atom	Atom	Angle/°
O1	C7	C6	133.2(2)
O1	C7	C8	117.4(2)
C6	C7	C8	109.5(2)
O2	C8	N2	112.2(2)
O2	C8	C7	104.97(19)
O2	C8	C9	111.56(19)
N2	C8	C7	104.66(19)
N2	C8	C9	110.9(2)
C7	C8	C9	112.3(2)
C8	C9	C21	111.23(19)
C10	C9	C8	114.7(2)
C10	C9	C21	110.9(2)
C16A	C17A	C18	129.9(19)
C17	C18	C19	114.5(3)
C19	C18	C17A	111.9(8)
C18	C19	C20	114.3(2)
C19	C20	C21	114.2(2)
C20	C21	C9	111.5(2)
C22	C21	C9	109.8(2)
C22	C21	C20	110.2(2)
C23	C22	C21	112.3(2)
O2	C23	C22	109.70(19)
O2	C23	C24	112.8(2)
C24	C23	C22	114.1(2)

Torsion Angles in ° for **2**.

Atom	Atom	Atom	Atom	Angle/°
O1	C7	C8	O2	-60.4(3)
O1	C7	C8	N2	-178.7(2)
O1	C7	C8	C9	61.0(3)
O2	C8	C9	C10	-177.6(2)
O2	C8	C9	C21	-50.8(3)
N1	C1	C2	C3	-0.3(3)
N1	C4	C5	N2	6.7(4)
N1	C4	C5	C6	-174.1(2)
N2	C5	C6	C7	-0.8(3)
N2	C8	C9	C10	-51.8(3)
N2	C8	C9	C21	75.0(2)
N3	C10	C11	C12	0.9(3)
N3	C13	C14	C15	93.1(4)
N3	C13	C14	C15A	56.3(5)
C1	N1	C4	C3	0.4(3)
C1	N1	C4	C5	-176.1(2)
C1	C2	C3	C4	0.5(3)
C2	C3	C4	N1	-0.6(3)
C2	C3	C4	C5	175.4(3)
C3	C4	C5	N2	-168.8(3)
C3	C4	C5	C6	10.4(4)
C4	N1	C1	C2	-0.1(3)
C4	C5	C6	C7	179.9(2)
C5	N2	C8	O2	-115.1(2)
C5	N2	C8	C7	-1.8(2)
C5	N2	C8	C9	119.5(2)
C13	N3	C10	C11	-1.4(3)
C13	C14	C15	C16	-63.3(5)
C13	C14	C15A	C16A	58.9(9)
C14	C15	C16	C17	79.3(8)
C14	C15A	C16A	C17A	-81.7(18)
C15	C16	C17	C18	-151.5(5)
C15A	C16A	C17A	C18	-54(3)
C16	C17	C18	C19	172.2(5)
C16A	C17A	C18	C19	171.4(18)
C17	C18	C19	C20	-61.5(6)
C17A	C18	C19	C20	-72.1(10)
C18	C19	C20	C21	-71.1(3)
C5	C6	C7	O1	179.6(3)
C5	C6	C7	C8	-0.4(3)
C6	C7	C8	O2	119.6(2)
C6	C7	C8	N2	1.4(3)
C6	C7	C8	C9	-119.0(2)
C7	C8	C9	C10	64.9(3)
C7	C8	C9	C21	-168.3(2)
C8	O2	C23	C22	-54.8(3)
C8	O2	C23	C24	73.5(3)
C8	N2	C5	C4	-179.0(2)
C8	N2	C5	C6	1.7(3)
C8	C9	C10	N3	79.3(3)
C8	C9	C10	C11	-116.0(3)
C8	C9	C21	C20	174.2(2)
C8	C9	C21	C22	51.9(3)
C9	C10	C11	C12	-165.7(2)
C9	C21	C22	C23	-54.5(3)
C10	N3	C13	C12	1.4(3)
C10	N3	C13	C14	-174.9(2)
C10	C9	C21	C20	-56.9(3)
C10	C9	C21	C22	-179.3(2)
C10	C11	C12	C13	-0.1(3)
C11	C12	C13	N3	-0.8(3)
C11	C12	C13	C14	175.0(3)
C12	C13	C14	C15	-82.2(4)
C13	N3	C10	C9	166.2(2)
C19	C20	C21	C9	150.5(2)
C19	C20	C21	C22	-87.4(3)
C20	C21	C22	C23	-177.6(2)
C21	C9	C10	N3	-47.7(3)
C21	C9	C10	C11	117.0(3)
C21	C22	C23	O2	54.0(3)
C21	C22	C23	C24	-73.6(3)
C23	O2	C8	N2	-71.3(2)
C23	O2	C8	C7	175.62(19)
C23	O2	C8	C9	53.7(3)
C25	O1	C7	C6	2.2(4)
C25	O1	C7	C8	-177.7(2)

Hydrogen Fractional Atomic Coordinates (×10^4) and Equivalent Isotropic Displacement Parameters (Å^2×10^3) for **2**. U_{eq} is defined as 1/3 of the trace of the orthogonalised U_{ij}.

Atom	x	y	z	*U~eq~*
H3B	8250(60)	5750(40)	3040(20)	88(16)
H3C	9540(50)	5240(30)	2658(11)	71(13)
H1	225.6	7189.8	3020.87	28
H3	1149.39	5203.97	3880.29	26
H1A	-1790.08	8589.96	2939.7	32
H2	-609.92	10183.97	3458.49	31
H3A	2206.62	9664.69	3863.52	27
H6	4781.98	8395.89	4150.61	24
H9	5599.3	4837.18	3970.87	24
H11	4645.47	4903.83	5129.56	29
H12	1876.44	4690.05	5650.11	34
H14C	-1489.26	5451.2	4716.13	42
H14D	-1246.72	4618.97	5263.24	42
H14A	-1456.16	5196.23	4495.61	42
H14B	-1312.37	5096.63	5223.86	42
H15A	-2873.56	3702.66	4870.31	36
H15B	-1197.05	3228.57	5134.89	36
H15C	-1414.76	4116.44	3988.54	37
H15D	-2832.93	3898.66	4470.78	37
H16A	-1536.43	3601.77	3842.23	37
H16B	-2175.48	2513.66	4134.78	37
H16C	-1011.73	2517.94	4887.43	60
H16D	-2022.99	2286.58	4274.06	60
H17A	1149.66	3077.99	4380.94	42
H17B	398.06	1908.17	4315.84	42
H17C	452.64	1645.37	4202.58	66
H17D	1253.21	2735.9	4393.55	66
H18A	702.78	3310.58	3291.93	38
H18B	194.7	2089.99	3254.2	38
H18C	194.56	2177.83	3198.33	38
H18D	741.63	3359.85	3369.34	38
H19A	2895.03	2265.99	2924.06	33
H19B	2875.16	1587.33	3544.66	33
H20A	5023.23	2737.87	3655.45	30
H20B	3618.7	3187.48	4097.15	30
H21	2927.33	4326.7	3161.01	25
H22A	6347.9	3874.94	2972.18	29
H22B	4904.84	3573.9	2504.17	29
H23	6452.18	5103.27	2212.36	27
H24A	3021.92	5457.81	2322.92	39
H24B	3956.8	4984.29	1736.64	39
H24C	4207.02	6199.6	1923.99	39
H25A	7823.58	8178.2	4276.23	40
H25B	8895.13	7280.42	4606.19	40
H25C	7100.64	7557.74	4858.37	40

Atomic Occupancies for all atoms that are not fully occupied in **2**.

Atom	Occupancy	Atom	Occupancy	Atom	Occupancy
H14C	0.379(8)	H15D	0.379(8)	H17B	0.621(8)
H14D	0.379(8)	C16	0.621(8)	C17A	0.379(8)
H14A	0.621(8)	H16A	0.621(8)	H17C	0.379(8)
H14B	0.621(8)	H16B	0.621(8)	H17D	0.379(8)
C15	0.621(8)	C16A	0.379(8)	H18A	0.621(8)
H15A	0.621(8)	H16C	0.379(8)	H18B	0.621(8)
H15B	0.621(8)	H16D	0.379(8)	H18C	0.379(8)
C15A	0.379(8)	C17	0.621(8)	H18D	0.379(8)
H15C	0.379(8)	H17A	0.621(8)		

Supplementary Note 2. X-Ray Crystal Structure Determination for Gem-dimethyl-bridged premarineosin A.

Experimental. Single orange irregular-shaped crystals of **3** were used as supplied. A suitable crystal with dimensions 0.20 × 0.18 × 0.12 mm^3 was selected and mounted on a dtrek-CrysAlisPro-abstract goniometer imported rigaku-d*trek images diffractometer. The crystal was kept at a steady T = 85 K during data collection. The structure was solved with the ShelXT 2018/2[14] solution program using dual methods and by using Olex2 1.5-alpha[15] as the graphical interface. The model was refined with ShelXL 2019/3[16] using full matrix least squares minimisation on $\boldsymbol{F^2}$.

Crystal Data. $C_{57}H_{72}F_6N_6O_8$, M_r = 1083.20, orthorhombic, $C222_1$ (No. 20), a = 15.27900(10) Å, b = 20.9910(2) Å, c = 17.29930(10) Å, $a = b = g = 90^\circ$, V = 5548.26(7) Å^3, T = 85 K, Z = 4, Z' = 0.5, m(Cu K$_a$) = 0.838, 42424 reflections measured, 5150 unique (R_{int} = 0.0425) which were used in all calculations. The final wR_2 was 0.1663 (all data) and R_1 was 0.0614 (I≥2 s(I)).

Formula	$C_{57}H_{72}F_6N_6O_8$
$D_{calc.}$/ g cm^{-3}	1.297
m/mm^{-1}	0.838
Formula Weight	1083.20
Colour	orange
Shape	irregular-shaped
Size/mm^3	0.20×0.18×0.12
T/K	85
Crystal System	orthorhombic
Flack Parameter	0.05(6)
Hooft Parameter	0.04(5)
Space Group	$C222_1$
a/Å	15.27900(10)
b/Å	20.9910(2)
c/Å	17.29930(10)
$a/^\circ$	90
$b/^\circ$	90
$g/^\circ$	90
V/Å^3	5548.26(7)
Z	4
Z'	0.5
Wavelength/Å	1.54184
Radiation type	Cu K$_a$
$Q_{min}/^\circ$	3.578
$Q_{max}/^\circ$	69.305
Measured Refl's.	42424
Indep't Refl's	5150
Refl's I≥2 s(I)	4984
R_{int}	0.0425
Parameters	402
Restraints	157
Largest Peak	0.529
Deepest Hole	-0.386
GooF	1.069
wR_2 (all data)	0.1663
wR_2	0.1631
R_1 (all data)	0.0630
R_1	0.0614

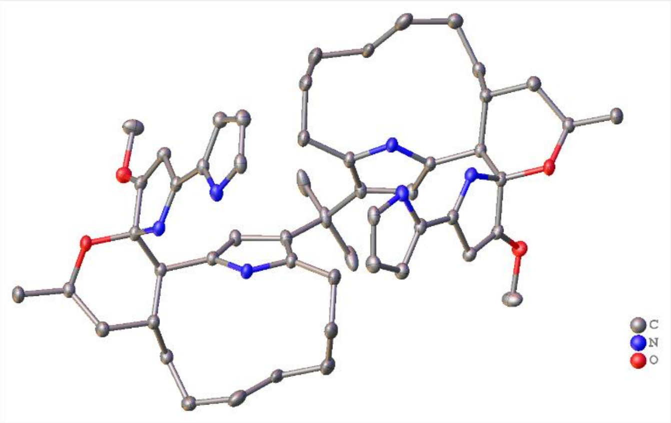

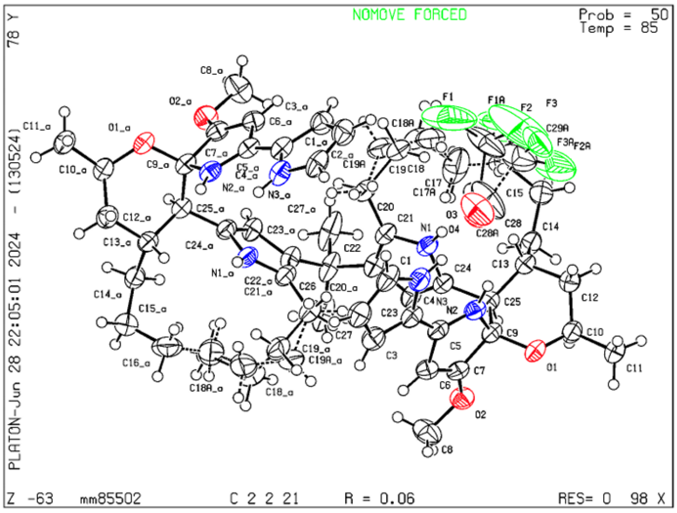

Structure Quality Indicators

**Reflections:**	d min (CuKα) 2Θ=138.6° 0.82	I/σ(I) 48.7	Rint m=8.25 4.25%	Full 135.4° 100	
**Refinement:**	Shift -0.001	Max Peak 0.5	Min Peak -0.4	GooF 1.069	Hooft .04(5)

An orange irregular-shaped-shaped crystal with dimensions 0.20 × 0.18 × 0.12 mm^3 was mounted. Data were collected using a dtrek-CrysAlisPro-abstract goniometer imported rigaku-d*trek images diffractometer operating at T = 85 K.

Data were measured using *w* scans with Cu K_a radiation. The diffraction pattern was indexed and the total number of runs and images was based on the strategy calculation from the program DTREK_VERSION=d*TREK version 9.9.9.4 W9RSSI – Aug 22 2012. The maximum resolution that was achieved was Q = 69.305° (0.82 Å).

The unit cell was refined using CrysAlisPro 1.171.43.127a[17] on 26227 reflections, 62% of the observed reflections.

Data reduction, scaling and absorption corrections were performed using CrysAlisPro 1.171.43.127a[17]. The final completeness is 100.00 % out to 69.305° in Q. A multi-scan absorption correction was performed using CrysAlisPro 1.171.43.127a[17]. Empirical absorption correction using spherical harmonics, implemented in SCALE3 ABSPACK scaling algorithm.. The absorption coefficient m of this material is 0.838 mm^{-1} at this wavelength (l = 1.54184Å) and the minimum and maximum transmissions are 0.972 and 1.000.

The structure was solved and the space group $C222_1$ (# 20) determined by the ShelXT 2018/2[14] structure solution program using using dual methods and refined by full matrix least squares minimization on $\boldsymbol{F}^2$ using version 2019/3 of ShelXL 2019/3[16]. All non-hydrogen atoms were refined anisotropically. Hydrogen atom positions were calculated geometrically and refined using the riding model. Hydrogen atom positions were calculated geometrically and refined using the riding model.

_refine_special_details: A trifluoroacetate was found disordered at two positions. It was modelled as such with the implementation of some restraints.

_exptl_absorpt_process_details: CrysAlisPro 1.171.43.127a[17] using spherical harmonics, implemented in SCALE3 ABSPACK scaling algorithm.

The value of Z' is 0.5. This means that only half of the formula unit is present in the asymmetric unit, with the other half consisting of symmetry equivalent atoms. The moiety formula is C53 H72 N6 O4, C4 F6 O4.

The Flack parameter was refined to 0.05(6). Determination of absolute structure using Bayesian statistics on Bijvoet differences using the Olex2 results in 0.04(5). The chiral atoms in this structure are: C9(S), C10(R), C13(R), C25(R). Note: The Flack parameter is used to determine chirality of the crystal studied, the value should be near 0, a value of 1 means that the stereochemistry is wrong and the model should be inverted. A value of 0.5 means that the crystal consists of a racemic mixture of the two enantiomers.

Fractional Atomic Coordinates (×104) and Equivalent Isotropic Displacement Parameters (Å2×103) for 3. Ueq is defined as 1/3 of the trace of the orthogonalised Uij.

Atom	x	y	z	U_{eq}
O1	2633.1(16)	5361.9(12)	1239.3(13)	42.8(6)
O2	1469.0(17)	4381.1(13)	1833.2(16)	47.4(6)
N1	2151(2)	5798.2(15)	3788.8(16)	41.9(7)
N2	3311.9(18)	5256.4(14)	2466.1(16)	38.4(6)
N3	4852.7(19)	4936.6(16)	3502.1(17)	45.4(7)
C1	5425(2)	4610(2)	3944(2)	51.0(9)
C2	5162(3)	3986(2)	3998(2)	53.2(9)
C3	4391(3)	3932.2(19)	3585(2)	47.9(8)
C4	4194(2)	4530.8(19)	3265.6(19)	41.5(8)
C5	3483(2)	4692.0(17)	2781.6(18)	38.4(7)
C6	2814(2)	4235.9(17)	2569(2)	40.4(7)
C7	2227(2)	4561.0(17)	2142.6(19)	38.6(7)
C8	1213(3)	3733(2)	2002(3)	63.8(12)
C9	2483(2)	5246.5(17)	2028.5(18)	37.4(7)
C10	2253(3)	5943.1(18)	917(2)	43.7(8)
C11	2652(3)	6003(2)	117(2)	52.6(9)
C12	2432(3)	6503.0(18)	1440(2)	47.7(8)
C13	2020(2)	6407.0(17)	2242(2)	40.9(7)
C14	1202(3)	6814.8(19)	2387(2)	46.7(8)
C15	1389(4)	7469(2)	2728(3)	59.6(11)
C16	1801(4)	7463(2)	3532(3)	72.1(14)
C17	1226(14)	7146(7)	4159(7)	63(5)
C17A	1360(20)	7100(17)	4191(14)	106(15)
C18	1562(13)	7072(6)	4998(7)	74(4)
C18A	2090(30)	7106(9)	4796(16)	99(10)
C19	2386(10)	6663(4)	5124(6)	57(3)
C19A	1890(20)	6618(7)	5401(11)	73(7)
C20	2218(3)	5939(2)	5203(2)	51.4(9)
C21	1805(2)	5653.8(18)	4497.7(19)	42.5(8)
C22	1060(2)	5299.7(19)	4383.1(19)	43.0(8)
C23	941(2)	5252.5(18)	3562.3(19)	40.9(7)
C24	1616(2)	5561.9(16)	3215.3(18)	38.4(7)
C25	1795(2)	5698.4(16)	2381.9(18)	37.1(7)
C26	486(4)	5000	5000	54.6(15)
C27	-102(3)	4482(3)	4642(2)	78.9(18)
C29	4742(3)	7121.6(14)	3908(3)	106(4)
F1	4977(4)	7077(3)	4659(3)	250(9)
F2	4023(3)	7496.0(16)	3898(4)	140(4)
F3	5384(3)	7467.0(15)	3560(5)	245(8)
O3	3840.0(14)	6333.3(13)	3347.6(19)	61.1(8)
O4	5308.8(15)	6175.0(16)	3364(3)	83.2(12)
C28	4613.8(15)	6458.1(10)	3494.7(17)	74(3)
C29A	4736(5)	7216(2)	3306(4)	94(4)
F1A	4486(7)	7505(5)	3969(5)	121(5)
F2A	4232(7)	7477(4)	2741(6)	148(6)
F3A	5559(5)	7423(4)	3169(6)	107(4)
C28A	4641(6)	6468(2)	3319(8)	97(6)

Anisotropic Displacement Parameters (×10^4) for **3**. The anisotropic displacement factor exponent takes the form: $-2p^2[h^2a^{*2} \times U_{11} + ... +2hka^* \times b^* \times U_{12}]$.

Atom	U_{11}	U_{22}	U_{33}	U_{23}	U_{13}	U_{12}
O1	47.3(13)	53.4(13)	27.7(11)	-2.9(9)	1.3(10)	0.2(11)
O2	41.9(13)	50.8(14)	49.6(14)	-2.6(11)	-12.5(11)	-5.7(11)
N1	49.9(16)	46.7(15)	29.3(13)	-5.4(11)	-1.2(12)	-4.6(13)
N2	35.3(13)	47.0(15)	32.7(12)	-5.5(12)	0.4(11)	-6.2(12)

Atom	U_{11}	U_{22}	U_{33}	U_{23}	U_{13}	U_{12}
N3	42.7(16)	58.8(18)	34.8(14)	1.6(13)	-1.6(12)	-10.1(14)
C1	38.2(18)	82(3)	33.1(16)	0.7(17)	-4.8(14)	-0.4(18)
C2	48(2)	66(2)	46.3(19)	3.3(18)	-9.5(17)	3.5(18)
C3	45.1(19)	55(2)	44.0(19)	-1.9(16)	-4.9(15)	2.5(16)
C4	34.8(16)	57(2)	32.3(15)	-4.2(14)	1.1(13)	-4.1(14)
C5	37.3(16)	48.4(18)	29.5(14)	-5.9(13)	2.4(12)	-4.1(14)
C6	38.2(17)	42.6(16)	40.2(17)	-5.1(14)	-3.6(14)	-3.0(14)
C7	35.4(16)	46.9(17)	33.4(15)	-8.5(13)	-4.3(13)	-2.6(14)
C8	52(2)	51(2)	88(3)	4(2)	-22(2)	-14.9(18)
C9	36.3(16)	47.5(17)	28.3(15)	-3.9(13)	0.5(12)	-4.4(14)
C10	48.5(19)	50.2(18)	32.5(16)	1.0(14)	0.4(14)	-1.9(15)
C11	67(2)	57(2)	33.5(17)	0.6(16)	6.6(17)	-4.2(19)
C12	55(2)	49.2(19)	39.3(18)	0.2(15)	5.8(16)	-5.8(16)
C13	46.7(18)	43.1(17)	33.0(16)	-2.1(13)	2.4(14)	-0.8(14)
C14	53(2)	53(2)	34.1(16)	2.0(15)	-0.1(15)	8.4(17)
C15	82(3)	47(2)	50(2)	-0.8(17)	6(2)	8(2)
C16	93(4)	55(2)	68(3)	-26(2)	-12(3)	11(2)
C17	117(10)	37(5)	35(7)	-10(4)	-20(7)	12(6)
C17A	170(30)	91(18)	58(16)	8(11)	29(15)	76(17)
C18	118(10)	62(5)	42(5)	-16(4)	-11(6)	2(6)
C18A	190(30)	47(7)	62(12)	-14(8)	-43(16)	4(13)
C19	79(7)	56(4)	36(4)	-7(3)	-11(4)	-10(4)
C19A	119(17)	52(7)	47(8)	-17(6)	-12(10)	15(8)
C20	66(2)	57(2)	31.3(17)	-5.3(15)	-5.9(16)	0.2(19)
C21	47.0(19)	51.3(19)	29.2(14)	-3.6(14)	-0.1(14)	3.5(16)
C22	38.6(16)	60(2)	30.4(16)	3.7(14)	-0.7(13)	2.1(16)
C23	36.1(15)	55.7(19)	30.8(16)	1.6(14)	-2.6(13)	-1.0(14)
C24	41.7(17)	42.9(16)	30.5(15)	-3.3(13)	-2.1(13)	1.8(13)
C25	38.1(16)	44.2(17)	29.1(15)	-2.4(13)	0.9(12)	-1.3(14)
C26	40(3)	96(4)	28(2)	10(3)	0	0
C27	57(2)	142(5)	38(2)	25(3)	-9.1(18)	-42(3)
C29	92(6)	42(4)	183(10)	23(5)	-95(7)	-17(4)
F1	440(20)	78(4)	238(11)	-51(6)	-256(13)	43(8)
F2	169(8)	63(4)	187(9)	-23(5)	-94(7)	-3(5)
F3	142(9)	114(8)	480(20)	9(12)	-69(13)	-89(7)
O3	48.0(15)	59.4(17)	76(2)	2.4(15)	-18.2(15)	-7.6(12)
O4	53.7(18)	66(2)	130(4)	16(2)	-24(2)	-4.4(14)
C28	61(5)	59(6)	102(7)	2(5)	-41(5)	-16(4)
C29A	93(8)	59(6)	129(10)	-6(7)	-33(8)	15(6)
F1A	178(13)	55(5)	130(8)	-36(5)	-59(8)	-22(7)
F2A	196(12)	62(5)	184(11)	-7(6)	-105(11)	35(6)
F3A	143(8)	53(4)	126(7)	34(4)	-77(6)	-29(5)
C28A	60(6)	53(8)	178(16)	42(8)	-19(9)	6(6)

Bond Lengths in Å for **3**.

Atom	Atom	Length/Å
O1	C9	1.405(4)
O1	C10	1.462(5)
O2	C7	1.330(4)
O2	C8	1.445(5)
N1	C21	1.369(5)
N1	C24	1.378(4)
N2	C5	1.330(5)
N2	C9	1.475(4)
N3	C1	1.349(5)
N3	C4	1.380(5)
C1	C2	1.372(7)
C2	C3	1.383(6)
C3	C4	1.405(6)

Atom	Atom	Length/Å
C17A	C18A	1.53(2)
C18	C19	1.54(2)
C18A	C19A	1.49(5)
C19	C20	1.547(9)
C19A	C20	1.548(14)
C20	C21	1.499(5)
C21	C22	1.373(5)
C22	C23	1.435(4)
C22	C26	1.518(5)
C23	C24	1.359(5)
C24	C25	1.495(4)
C26	C27[1]	1.542(6)
C26	C27	1.542(6)

Atom	Atom	Length/Å
C4	C5	1.413(5)
C5	C6	1.447(5)
C6	C7	1.348(5)
C7	C9	1.504(5)
C9	C25	1.542(5)
C10	C11	1.517(5)
C10	C12	1.508(5)
C12	C13	1.537(5)
C13	C14	1.536(5)
C13	C25	1.546(5)
C14	C15	1.522(6)
C15	C16	1.526(7)
C16	C17	1.547(15)
C16	C17A	1.53(2)
C17	C18	1.548(15)

Atom	Atom	Length/Å
C29	F1	1.3500
C29	F2	1.3506
C29	F3	1.3607
C29	C28	1.5779
O3	C28	1.2374
O3	C28A	1.257(10)
O4	C28	1.2376
O4	C28A	1.193(10)
C29A	F1A	1.3508
C29A	F2A	1.3604
C29A	F3A	1.3508
C29A	C28A	1.5785
F2A	F3A^{2}	1.61(2)

^{1}+x,1-y,1-z; ^{2}1-x,+y,1/2-z

Bond Angles in ° for **3**.

Atom	Atom	Atom	Angle/°
C9	O1	C10	116.7(3)
C7	O2	C8	115.0(3)
C21	N1	C24	109.7(3)
C5	N2	C9	111.5(3)
C1	N3	C4	109.1(3)
N3	C1	C2	109.5(3)
C1	C2	C3	107.0(4)
C2	C3	C4	108.2(4)
N3	C4	C3	106.2(3)
N3	C4	C5	126.1(3)
C3	C4	C5	127.7(3)
N2	C5	C4	127.4(3)
N2	C5	C6	110.3(3)
C4	C5	C6	122.3(3)
C7	C6	C5	105.9(3)
O2	C7	C6	131.0(3)
O2	C7	C9	116.5(3)
C6	C7	C9	112.5(3)
O1	C9	N2	110.9(3)
O1	C9	C7	109.6(3)
O1	C9	C25	113.0(3)
N2	C9	C7	99.8(3)
N2	C9	C25	111.9(3)
C7	C9	C25	111.0(3)
O1	C10	C11	105.0(3)
O1	C10	C12	110.5(3)
C12	C10	C11	114.2(3)
C10	C12	C13	111.5(3)
C12	C13	C25	111.0(3)
C14	C13	C12	114.0(3)
C14	C13	C25	109.3(3)
C15	C14	C13	114.4(3)
C14	C15	C16	115.0(4)
C15	C16	C17	114.1(7)
C15	C16	C17A	120.0(12)
C16	C17	C18	120.8(13)
C16	C17A	C18A	100.4(19)
C19	C18	C17	117.3(14)
C19A	C18A	C17A	109(3)
C18	C19	C20	115.2(12)
C18A	C19A	C20	114(3)
C21	C20	C19	113.0(4)

Atom	Atom	Atom	Angle/°
C21	C20	C19A	114.4(6)
N1	C21	C20	118.6(3)
N1	C21	C22	108.1(3)
C22	C21	C20	133.1(3)
C21	C22	C23	106.6(3)
C21	C22	C26	127.0(3)
C23	C22	C26	126.4(3)
C24	C23	C22	107.9(3)
N1	C24	C25	121.1(3)
C23	C24	N1	107.7(3)
C23	C24	C25	131.1(3)
C9	C25	C13	112.2(3)
C24	C25	C9	112.9(3)
C24	C25	C13	112.1(3)
C22	C26	C22^{1}	109.4(4)
C22	C26	C27	110.3(2)
C22^{1}	C26	C27	109.1(3)
C22^{1}	C26	C27^{1}	110.3(2)
C22	C26	C27^{1}	109.1(3)
C27^{1}	C26	C27	108.6(6)
F1	C29	F2	105.6
F1	C29	F3	105.7
F1	C29	C28	114.1
F2	C29	F3	105.7
F2	C29	C28	114.0
F3	C29	C28	111.0
O3	C28	C29	113.5
O3	C28	O4	132.9
O4	C28	C29	113.6
F1A	C29A	F2A	105.6
F1A	C29A	C28A	114.1
F2A	C29A	C28A	111.0
F3A	C29A	F1A	105.6
F3A	C29A	F2A	105.7
F3A	C29A	C28A	114.1
C29A	F2A	F3A^{2}	124.2(10)
O3	C28A	C29A	108.3(5)
O4	C28A	O3	135.6(5)
O4	C28A	C29A	115.8(5)

^{1}+x,1-y,1-z; ^{2}1-x,+y,1/2-z

Torsion angles in ° for **3**.

Atom	Atom	Atom	Atom	Angle/°
O1	C9	C25	C13	-54.0(4)
O1	C9	C25	C24	178.2(3)
O1	C10	C12	C13	-61.3(4)
O2	C7	C9	O1	-65.5(4)
O2	C7	C9	N2	178.1(3)
O2	C7	C9	C25	59.9(4)
N1	C21	C22	C23	-2.3(4)
N1	C21	C22	C26	176.2(3)
N1	C24	C25	C9	82.8(4)
N1	C24	C25	C13	-45.1(4)
N2	C5	C6	C7	-2.4(4)
N2	C9	C25	C13	72.0(3)
N2	C9	C25	C24	-55.8(4)
N3	C1	C2	C3	-1.1(5)
N3	C4	C5	N2	-0.1(6)
N3	C4	C5	C6	-179.3(3)
C1	N3	C4	C3	-0.1(4)
C1	N3	C4	C5	-178.6(3)
C1	C2	C3	C4	1.0(5)
C2	C3	C4	N3	-0.6(4)
C2	C3	C4	C5	177.9(4)
C3	C4	C5	N2	-178.2(3)
C3	C4	C5	C6	2.5(6)
C4	N3	C1	C2	0.7(4)
C4	C5	C6	C7	176.9(3)
C5	N2	C9	O1	-116.6(3)
C5	N2	C9	C7	-1.2(3)
C5	N2	C9	C25	116.3(3)
C5	C6	C7	O2	-176.5(3)
C5	C6	C7	C9	1.6(4)
C6	C7	C9	O1	116.1(3)
C6	C7	C9	N2	-0.4(3)
C6	C7	C9	C25	-118.5(3)
C7	C9	C25	C13	-177.5(3)
C7	C9	C25	C24	54.7(4)
C8	O2	C7	C6	2.4(6)
C8	O2	C7	C9	-175.7(4)
C9	O1	C10	C11	170.0(3)
C9	O1	C10	C12	46.5(4)
C9	N2	C5	C4	-177.0(3)
C9	N2	C5	C6	2.3(4)
C10	O1	C9	N2	-115.9(3)
C10	O1	C9	C7	134.9(3)
C10	O1	C9	C25	10.6(4)
C10	C12	C13	C14	-106.1(4)
C10	C12	C13	C25	17.8(4)
C11	C10	C12	C13	-179.3(3)
C12	C13	C14	C15	-89.1(4)
C12	C13	C25	C9	36.8(4)
C12	C13	C25	C24	165.1(3)
C13	C14	C15	C16	-63.6(5)
C14	C13	C25	C9	163.4(3)
C14	C13	C25	C24	-68.3(4)
C14	C15	C16	C17	-61.4(9)
C14	C15	C16	C17A	-54.5(18)
C15	C16	C17	C18	177.2(11)
C15	C16	C17A	C18A	168.0(14)
C16	C17	C18	C19	-61.8(19)

Atom	Atom	Atom	Atom	Angle/°
C16	C17A	C18A	C19A	-164(3)
C17	C18	C19	C20	-85.1(18)
C17A	C18A	C19A	C20	86(4)
C18	C19	C20	C21	61.2(13)
C18A	C19A	C20	C21	-63(3)
C19	C20	C21	N1	47.4(8)
C19	C20	C21	C22	-125.4(8)
C19A	C20	C21	N1	84.3(14)
C19A	C20	C21	C22	-88.5(15)
C20	C21	C22	C23	171.1(4)
C20	C21	C22	C26	-10.4(7)
C21	N1	C24	C23	-1.6(4)
C21	N1	C24	C25	174.7(3)
C21	C22	C23	C24	1.3(4)
C21	C22	C26	C22^{1}	-42.5(3)
C21	C22	C26	C27	-162.5(4)
C21	C22	C26	C27^{1}	78.3(5)
C22	C23	C24	N1	0.1(4)
C22	C23	C24	C25	-175.7(4)
C23	C22	C26	C22^{1}	135.7(4)
C23	C22	C26	C27^{1}	-103.5(4)
C23	C22	C26	C27	15.7(6)
C23	C24	C25	C9	-101.9(4)
C23	C24	C25	C13	130.2(4)
C24	N1	C21	C20	-172.1(3)
C24	N1	C21	C22	2.4(4)
C25	C13	C14	C15	146.1(3)
C26	C22	C23	C24	-177.2(3)
F1	C29	C28	O3	109.1
F1	C29	C28	O4	-72.7
F2	C29	C28	O3	-12.3
F2	C29	C28	O4	165.9
F3	C29	C28	O3	-131.6
F3	C29	C28	O4	46.6
F1A	C29A	F2A	F3A^{2}	163.9(8)
F1A	C29A	F3A	F2A^{2}	-147.5(3)
F1A	C29A	C28A	O3	67.2(8)
F1A	C29A	C28A	O4	-106.9(10)
F2A	C29A	F3A	F2A^{2}	-35.9(3)
F2A	C29A	C28A	O3	-52.0(8)
F2A	C29A	C28A	O4	133.9(10)
F3A	C29A	F2A	F3A^{2}	52.3(8)
F3A	C29A	C28A	O3	-171.3(8)
F3A	C29A	C28A	O4	14.5(10)
C28A	C29A	F2A	F3A^{2}	-71.9(8)
C28A	C29A	F3A	F2A^{2}	86.4(3)

^{1}+x,1-y,1-z; ^{2}1-x,+y,1/2-z

Hydrogen Fractional Atomic Coordinates (×10[4]) and Equivalent Isotropic Displacement Parameters (Å^2×10^3) for **3**. U_{eq} is defined as 1/3 of the trace of the orthogonalised U_{ij}.

Atom	x	y	z	U_{eq}
H1	2640.15	6009.73	3711.32	50
H2	3650.53	5593.34	2512.48	46
H3	4892.76	5343.22	3383.65	55
H1A	5931.94	4784.27	4181.38	61
H2A	5454.23	3654.88	4268.57	64
H3A	4052.56	3555.43	3526.63	57
H6	2794.26	3797.17	2702.8	48
H8A	641.61	3645.84	1766.61	96
H8B	1172.98	3675.29	2563.01	96
H8C	1649.29	3438.81	1790.69	96
H10	1606.13	5885.76	865.34	52
H11A	3288.12	6051.09	162.52	79
H11B	2407.57	6377.79	-143.73	79
H11C	2518.44	5620.44	-184.75	79
H12A	2191.05	6894.73	1202.8	57
H12B	3072.06	6559.44	1496.33	57
H13	2468.22	6532.45	2635.43	49
H14A	887.49	6870.72	1891.38	56
H14B	808.49	6581.47	2742.83	56
H15A	833.21	7710.64	2753.48	72
H15B	1785.34	7700.97	2372.88	72
H16C	1861.89	7912.08	3699.42	86
H16D	2400.31	7289.87	3477.34	86
H16A	1924.2	7907.82	3689.1	86
H16B	2367.09	7235.05	3504.63	86
H17A	1075.92	6714.07	3970.45	76
H17B	671.21	7389.18	4183.98	76
H17C	1200.04	6660.33	4035.41	127
H17D	826.53	7323.39	4377.46	127
H18A	1681.8	7503.65	5204.66	89
H18B	1083.51	6887.95	5312.51	89
H18C	2656.05	7009.35	4546.91	119
H18D	2128.57	7533.19	5036.19	119
H19A	2686.43	6813.66	5596.79	68
H19B	2788.89	6733.04	4683.43	68
H19C	1252.52	6603.45	5483.91	87
H19D	2166.98	6754.32	5892.92	87
H20C	2860.14	5953.36	5126.12	62
H20D	2100.96	5657.12	5649.79	62
H20A	2781.47	5721.31	5304.19	62
H20B	1832.06	5863.98	5653.34	62
H23	472.13	5042.14	3306.88	49
H25	1235.8	5615.36	2099.17	45
H27A	-526.62	4680.76	4292.13	118
H27B	-414.49	4255.7	5053.68	118
H27C	259.66	4179.49	4351.74	118

Atomic Occupancies for all atoms that are not fully occupied in **3**.

Atom	Occupancy	Atom	Occupancy	Atom	Occupancy	Atom	Occupancy
H16C	0.40(3)	C18	0.60(3)	H19C	0.40(3)	C28	0.587(9)
H16D	0.40(3)	H18A	0.60(3)	H19D	0.40(3)	C29A	0.413(9)
H16A	0.60(3)	H18B	0.60(3)	H20C	0.40(3)	F1A	0.413(9)
H16B	0.60(3)	C18A	0.40(3)	H20D	0.40(3)	F2A	0.413(9)
C17	0.60(3)	H18C	0.40(3)	H20A	0.60(3)	F3A	0.413(9)
H17A	0.60(3)	H18D	0.40(3)	H20B	0.60(3)	C28A	0.413(9)
H17B	0.60(3)	C19	0.60(3)	C29	0.587(9)		
C17A	0.40(3)	H19A	0.60(3)	F1	0.587(9)		
H17C	0.40(3)	H19B	0.60(3)	F2	0.587(9)		
H17D	0.40(3)	C19A	0.40(3)	F3	0.587(9)		

Supplementary References

1. Adusumilli, R. & Mallick, P. Data conversion with ProteoWizard msConvert. in *Proteomics: Methods and Protocols* (eds. Comai, L., Katz, J. E. & Mallick, P.) 339–368 (Springer, New York, NY, 2017). doi:10.1007/978-1-4939-6747-6_23

2. Schmid, R. *et al.* Integrative analysis of multimodal mass spectrometry data in MZmine 3. *Nat. Biotechnol.* **41,** 447–449 (2023).

3. Wang, M. *et al.* Sharing and community curation of mass spectrometry data with Global Natural Products Social Molecular Networking. *Nat. Biotechnol.* **34,** 828–837 (2016).

4. Shannon, P. *et al.* Cytoscape: a software environment for integrated models of biomolecular interaction networks. *Genome Res.* **13,** 2498–2504 (2003).

5. Boonlarppradab, C., Kauffman, C. A., Jensen, P. R. & Fenical, W. Marineosins A and B, cytotoxic spiroaminals from a marine-derived actinomycete. *Org. Lett.* **10,** 5505–5508 (2008).

6. Salem, S. M. *et al.* Elucidation of final steps of the marineosins biosynthetic pathway through identification and characterization of the corresponding gene cluster. *J. Am. Chem. Soc.* **136,** 4565–4574 (2014).

7. Bierman, M. *et al.* Plasmid cloning vectors for the conjugal transfer of DNA from Escherichia coli to Streptomyces spp. *Gene* **116,** 43–49 (1992).

8. Lian, W. *et al.* Genome-wide transcriptome analysis reveals that a pleiotropic antibiotic regulator, AfsS, modulates nutritional stress response in Streptomyces coelicolor A3(2). *BMC Genomics* **9,** 56 (2008).

9. Pereira, F. *et al.* Optimized production of concanamycins using a rational metabolic engineering strategy. *Metab. Eng.* **88,** 63–76 (2025).

10. Snodgrass, H. M. Methods and applications for the identification and directed evolution of biocatalysts for chemical synthesis. (The University of Chicago, United States -- Illinois, 2023).

11. Fraley, A. E. et al. Function and Structure of MalA/MalA′, Iterative halogenases for late-stage C–H functionalization of indole alkaloids. *J. Am. Chem. Soc.* **139**, 12060–12068 (2017).

12. Chakraborty, S., Ortiz-Maldonado, M., Entsch, B. & Ballou, D. P. Studies on the mechanism of p-hydroxyphenylacetate 3-hydroxylase from *Pseudomonas aeruginosa*: a system composed of a small flavin reductase and a large flavin-dependent oxygenase. *Biochemistry* **49**, 372–385 (2010).

13. Gilchrist, C. L. M. & Chooi, Y.-H. clinker & clustermap.js: automatic generation of gene cluster comparison figures. *Bioinformatics* **37,** 2473–2475 (2021).

14. Usón, I. & Sheldrick, G. M. An introduction to experimental phasing of macromolecules illustrated by SHELX; new autotracing features. *Acta Crystallogr. Sect. Struct. Biol.* **74,** 106–116 (2018).

15. Dolomanov, O. V., Bourhis, L. J., Gildea, R. J., Howard, J. A. K. & Puschmann, H. OLEX2: a complete structure solution, refinement and analysis program. *J. Appl. Crystallogr.* **42,** 339–341 (2009).

16. Sheldrick, G. M. Crystal structure refinement with SHELXL. *Acta Crystallogr. Sect. C Struct. Chem.* **71,** 3–8 (2015).

17. PX018 - CrysAlis^{Pro}: an all-in-one software package for single crystal X-ray diffraction. https://rigaku.com/products/crystallography/x-ray-diffraction/application-notes/px018-crysalispro-software-package-single-crystal.
