# Supplementary material for: Metabolic engineering and late-stage functionalization expand the chemical space of the antimalarial premarineosin A
Source: Commun Chem. 2025 Dec 5;8:391. doi: 10.1038/s42004-025-01779-6 (PMC12680612; doi:10.1038/s42004-025-01779-6)
Supplement: Supplementary file 2 — Description of Additional Supplementary Files [file 42004_2025_1779_MOESM2_ESM.pdf]

Description of Additional Supplementary Files:

File: Supplementary Data 1

Description: NMR spectral data.

File: Supplementary Data 2

Description: CIF crystal structure file for (-)-premarineosin A (3).

File: Supplementary Data 3

Description: CIF crystal structure file for gem-dimethyl-bridged premarineosin A (4).

File: Supplementary Data 4

Description: Raw data underlying main text figures.
