# Supplementary material for: Metabolic engineering and late-stage functionalization expand the chemical space of the antimalarial premarineosin A
Source: Commun Chem. 2025 Dec 5;8:391. doi: 10.1038/s42004-025-01779-6 (PMC12680612; doi:10.1038/s42004-025-01779-6)
Supplement: Supplementary file 3 — Supplementary Data 1 [file 42004_2025_1779_MOESM3_ESM.pdf]

Supplementary Data: NMR Spectra

Metabolic engineering and late-stage functionalization expand the chemical space of the antimalarial premarineosin A

Christina M. McBride[a,b,†], Morgan McCauley[a,†], Natalia R. Harris[a,c], Sahar Amin[a,d], Brian J. Curtis[a], Linnea Verhey-Henke[a], Awet A. Teklemichael[e], Erin N. Oliphant[f], Patricia Dranchak[f], Katherine L. Lev[a,b], Fengrui Qu[g], Harrison M. Snodgrass[h], Jared C. Lewis[h,*], James Inglese[f,i], Xin-zhuan Su[e], Filipa Pereira[a,c,#,*], David H. Sherman[a,d,g,j,#,*]

[a] *Life Sciences Institute, University of Michigan, Ann Arbor, Michigan, USA*
[b] *Program in Chemical Biology, University of Michigan, Ann Arbor, Michigan, USA*
[c] *Department of Biological Chemistry, University of Michigan Medical School, Ann Arbor, Michigan, USA*
[d] *Department of Medicinal Chemistry, University of Michigan, Ann Arbor, Michigan, USA*
[e] *Laboratory of Malaria and Vector Research, National Institute of Allergy and Infectious Diseases, NIH, Rockville, Maryland, USA*
[f] *National Center for Advancing Translational Sciences (NCATS), NIH, Rockville, Maryland, USA*
[g] *Department of Chemistry, University of Michigan, Ann Arbor, Michigan, USA*
[h] *Department of Chemistry, Indiana University, Bloomington, Indiana, USA*
[i] *Metabolic Medicine Branch, National Human Genome Research Institute, NIH, Bethesda, Maryland, USA*
[j] *Department of Microbiology and Immunology, University of Michigan, Ann Arbor, Michigan, USA*

[†] *These authors contributed equally.*
[#] *These authors jointly supervised this work*
[*] *Corresponding author. E-mail address: davidhs@umich.edu, fpere@umich.edu, jcl3@iu.edu*

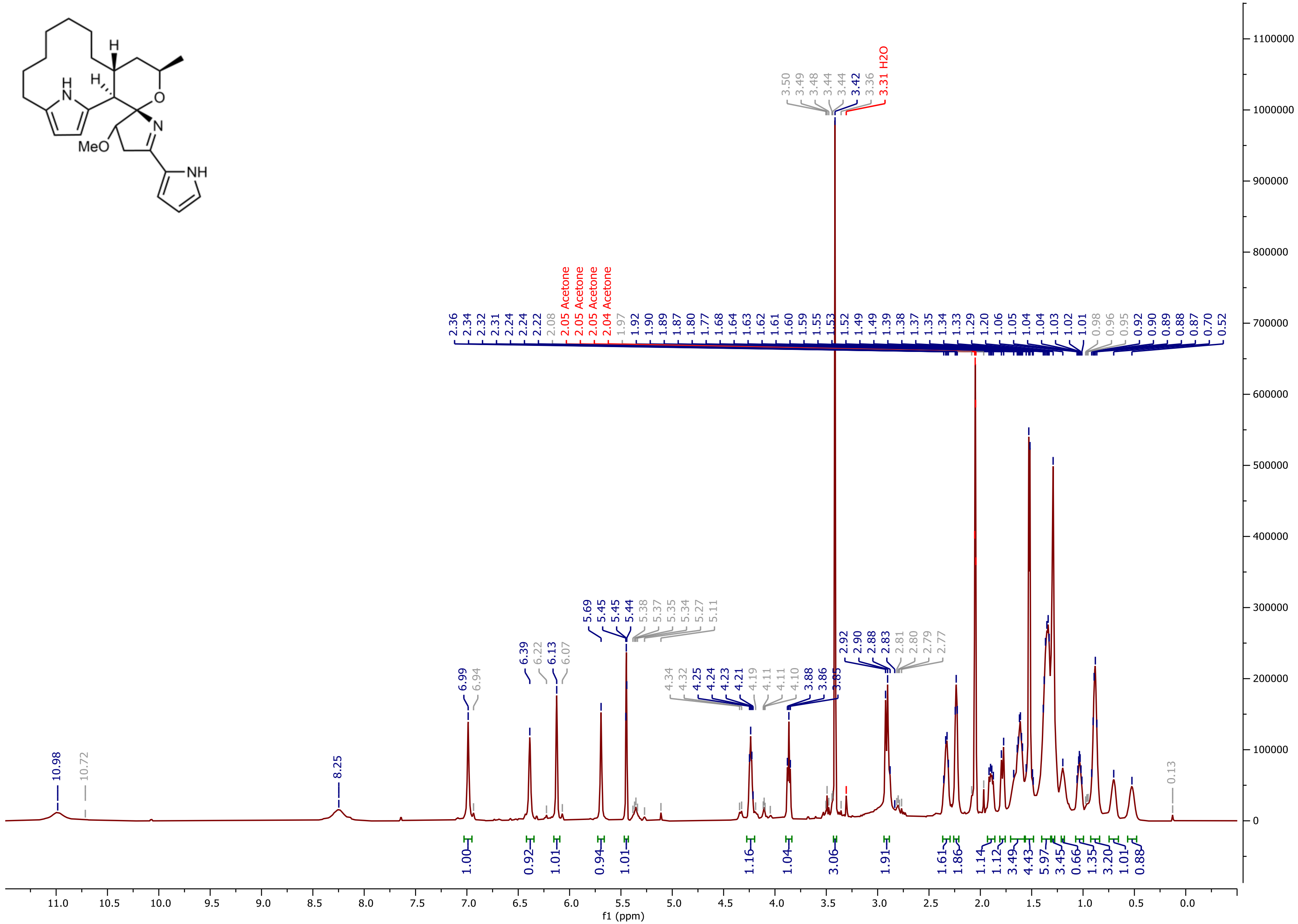

NMR Spectrum 1. ^{1}H NMR Spectrum (600 MHz) of marineosin A (**1**) in acetone-D_6.

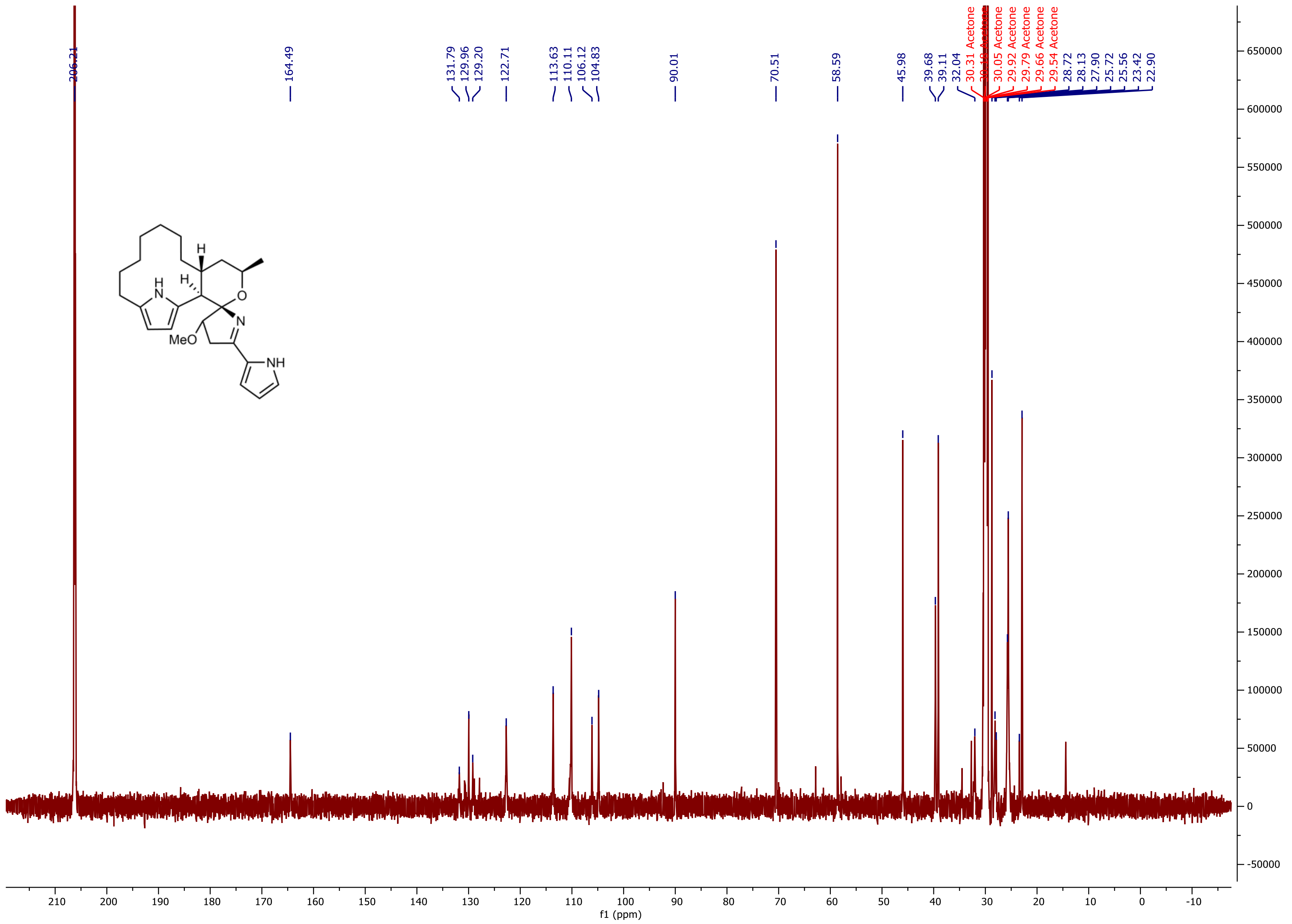

NMR Spectrum 2. ^{13}C NMR Spectrum (151 MHz) of marineosin A (**1**) in acetone-D$_6$.

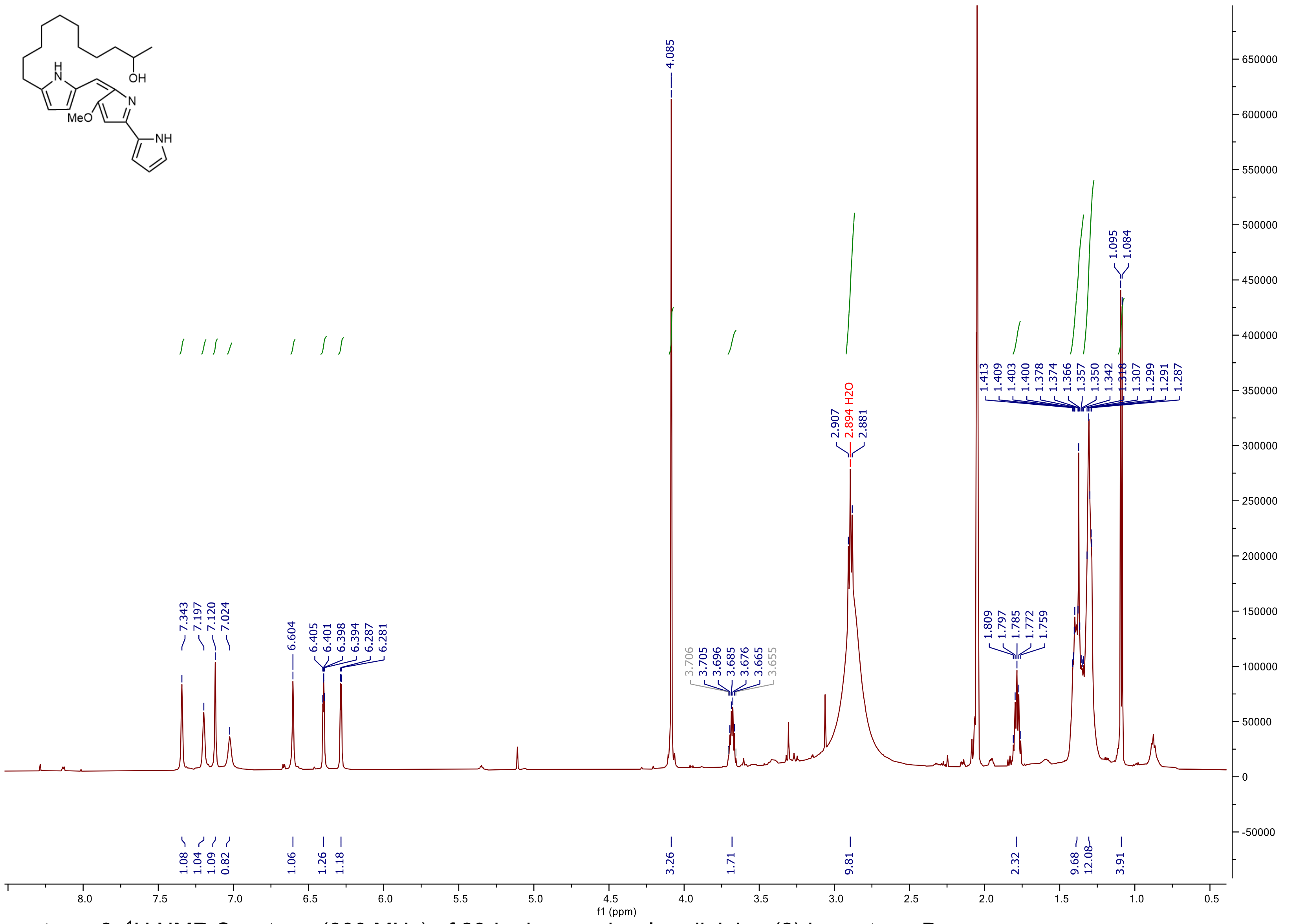

NMR Spectrum 3. ^{1}H NMR Spectrum (600 MHz) of 23-hydroxyundecylprodiginine (**2**) in acetone-D$_6$.

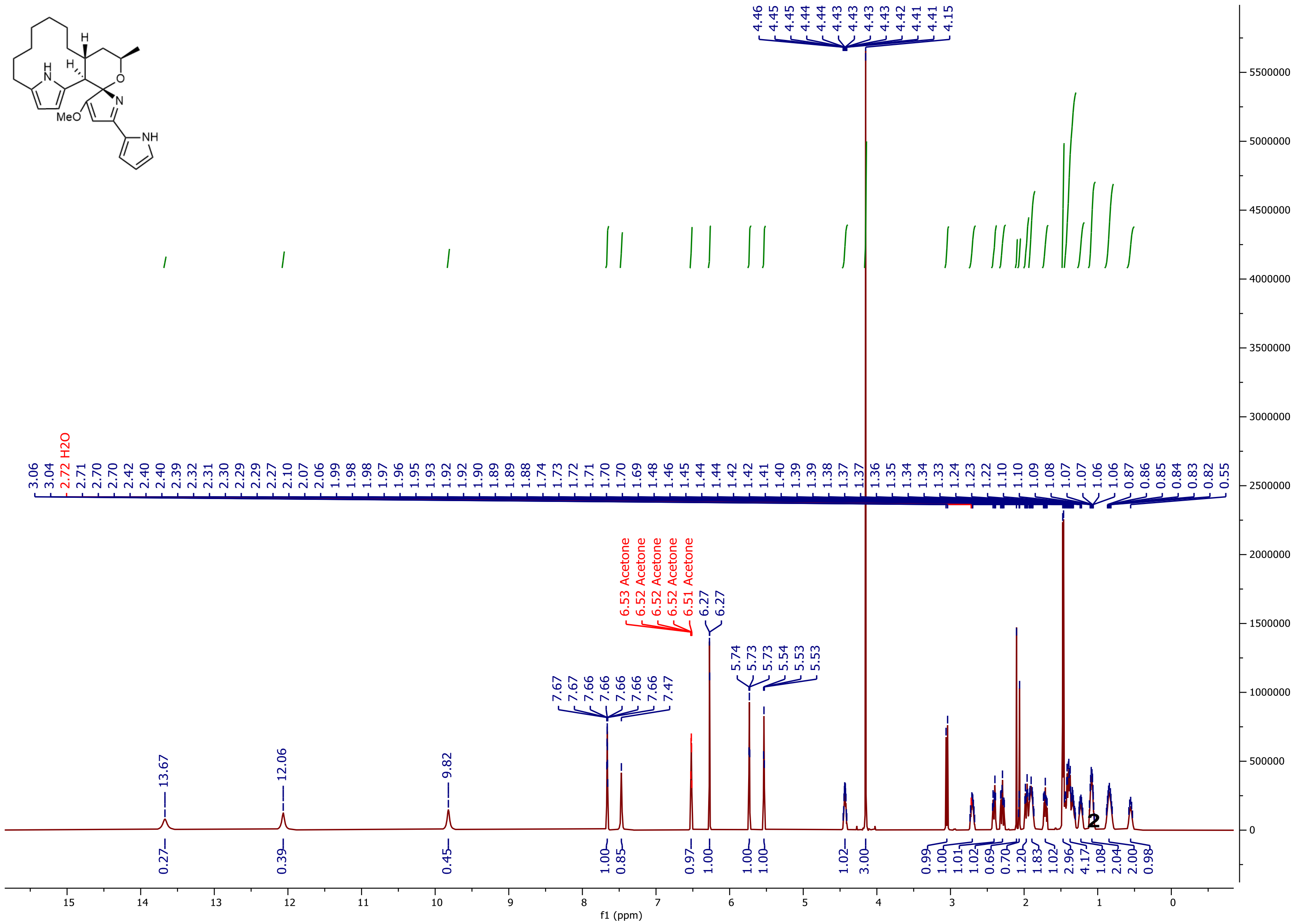

NMR Spectrum 4. ^{1}H NMR Spectrum (600 MHz) of (−)-premarineosin A (**3**) in acetone-D$_6$.

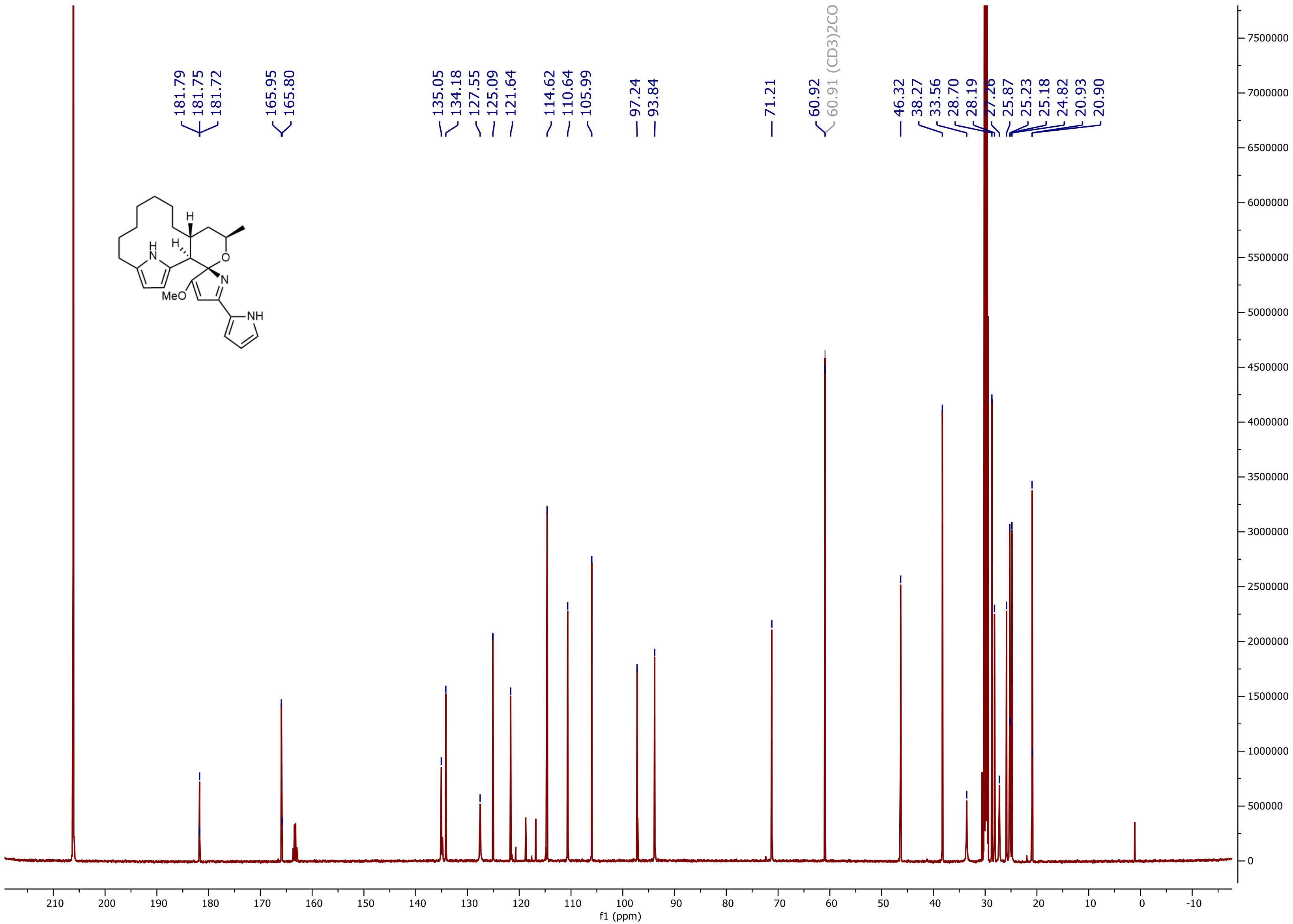

NMR Spectrum 5. ^{13}C NMR Spectrum (151 MHz) of (−)-premarineosin A (**3**) in acetone-D_6.

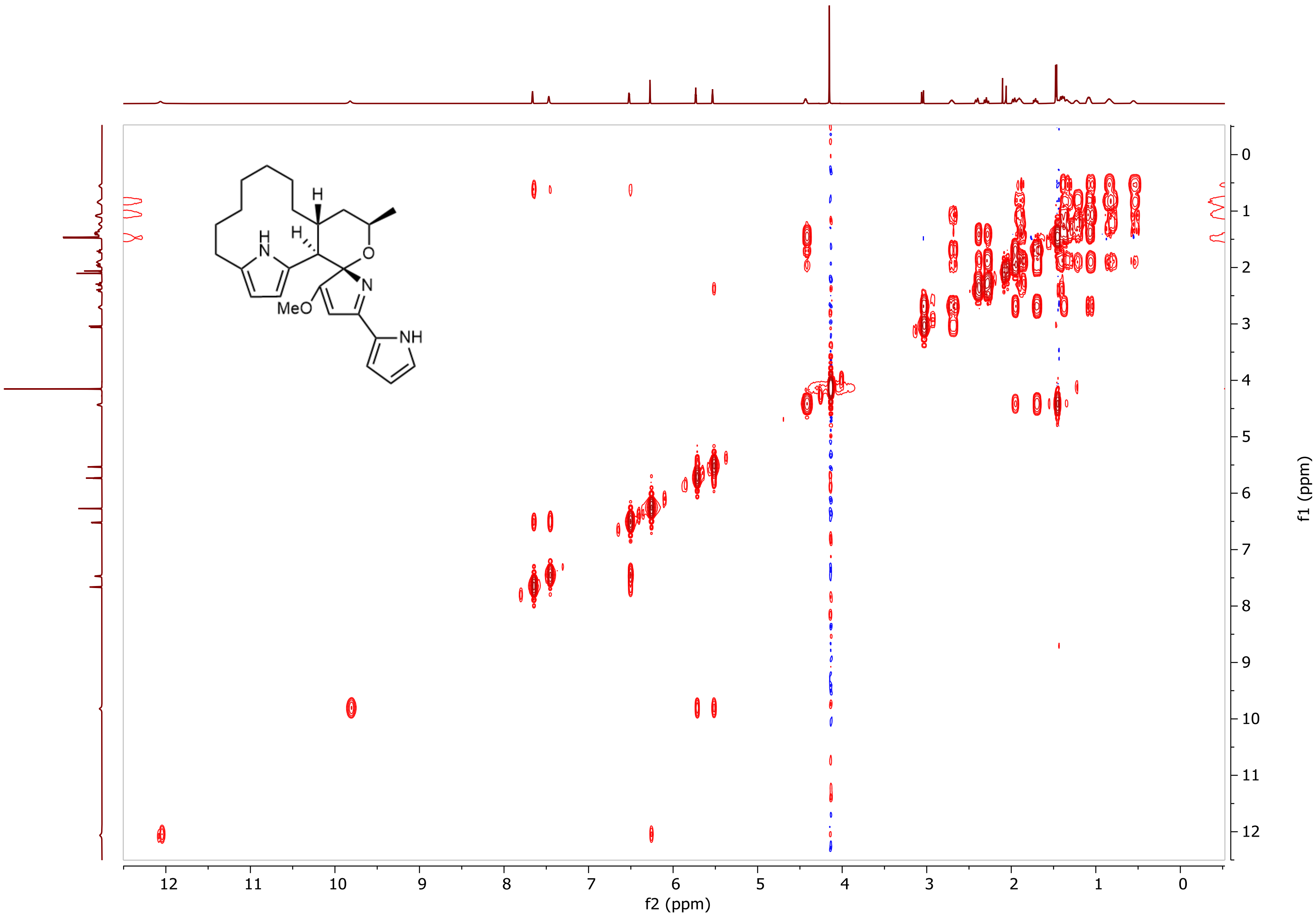

NMR Spectrum 6. ^{1}H-^{1}H COSY NMR Spectrum of (−)-premarineosin A (**3**) in acetone-D_6.

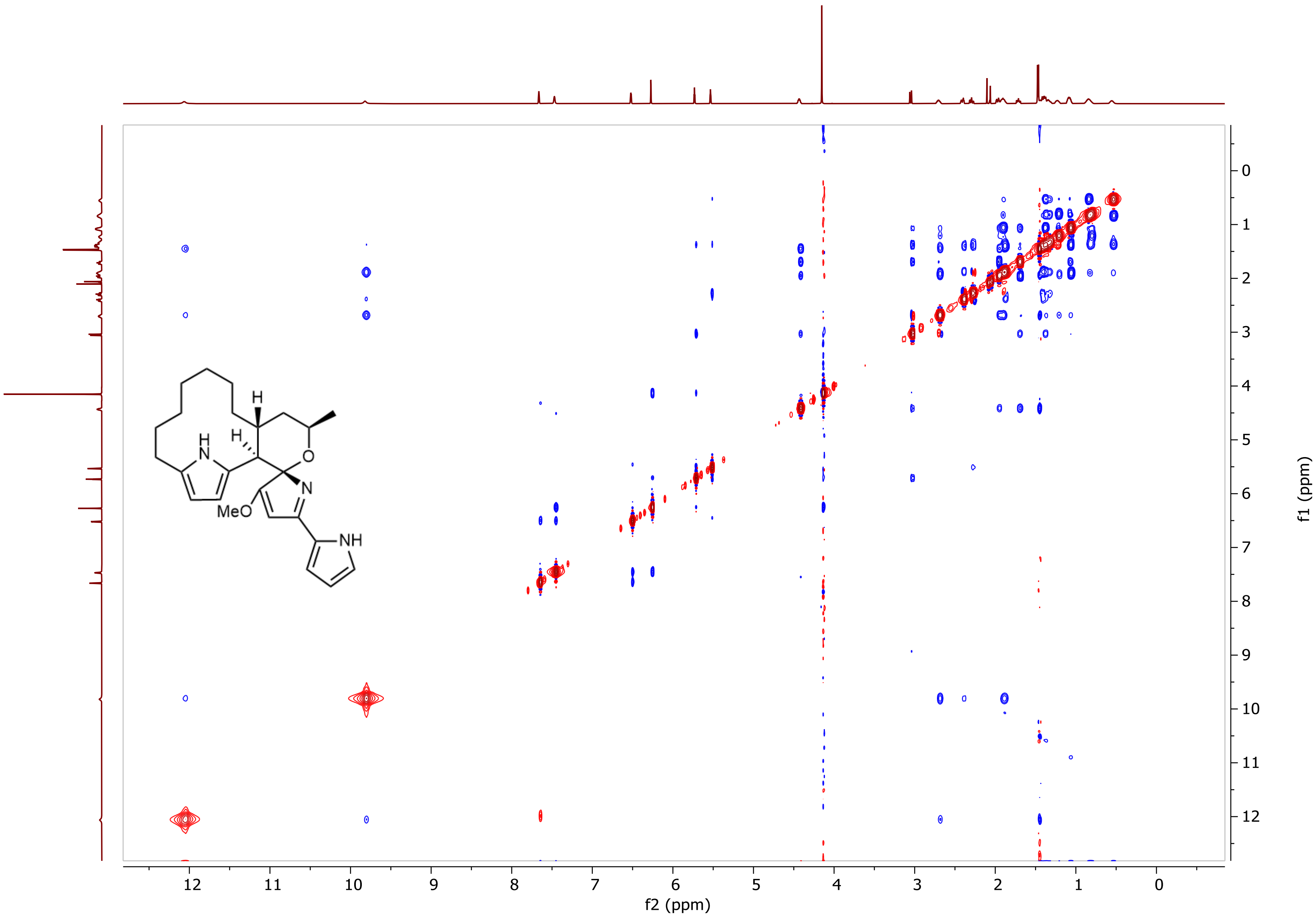

NMR Spectrum 7. ^{1}H-^{1}H NOESY NMR Spectrum of **(−)**-premarineosin A (**3**) in acetone-D_6.

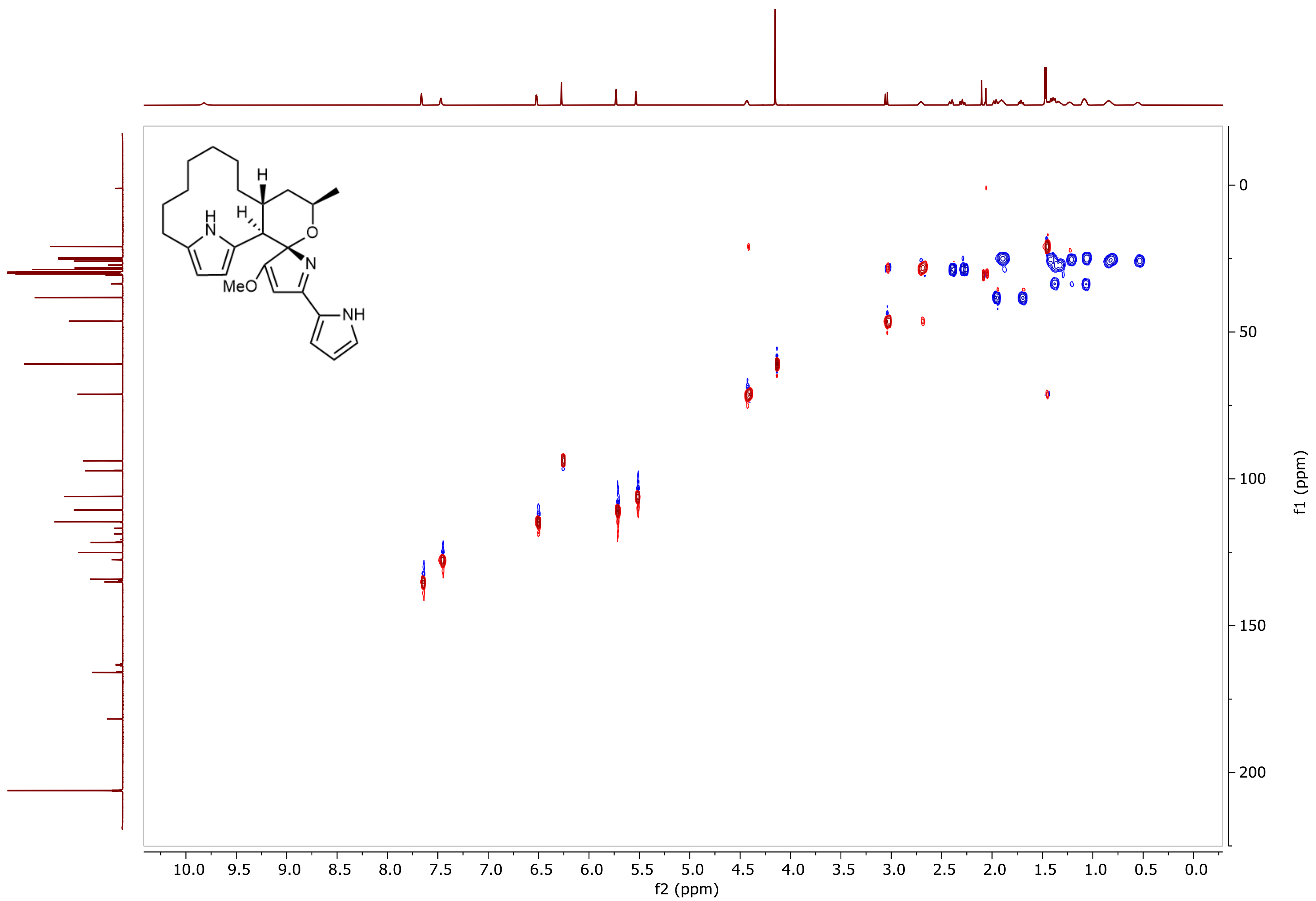

NMR Spectrum 8. ^{1}H-^{13}C HSQC NMR Spectrum of (−)-premarineosin A (**3**) in acetone-D_6.

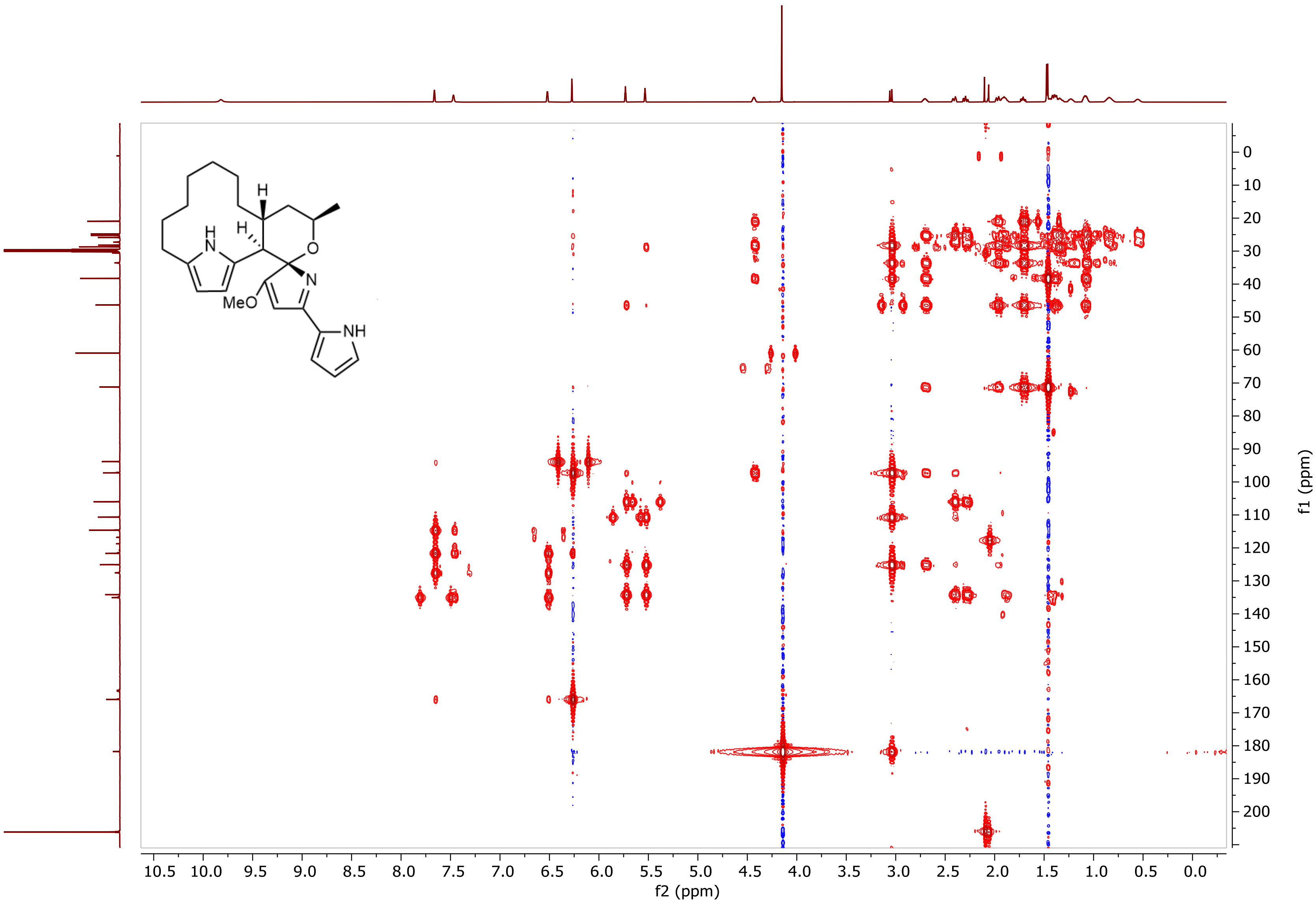

NMR Spectrum 9. ^{1}H-^{13}C HMBC NMR Spectrum of (−)-premarineosin A (**3**) in acetone-D_6.

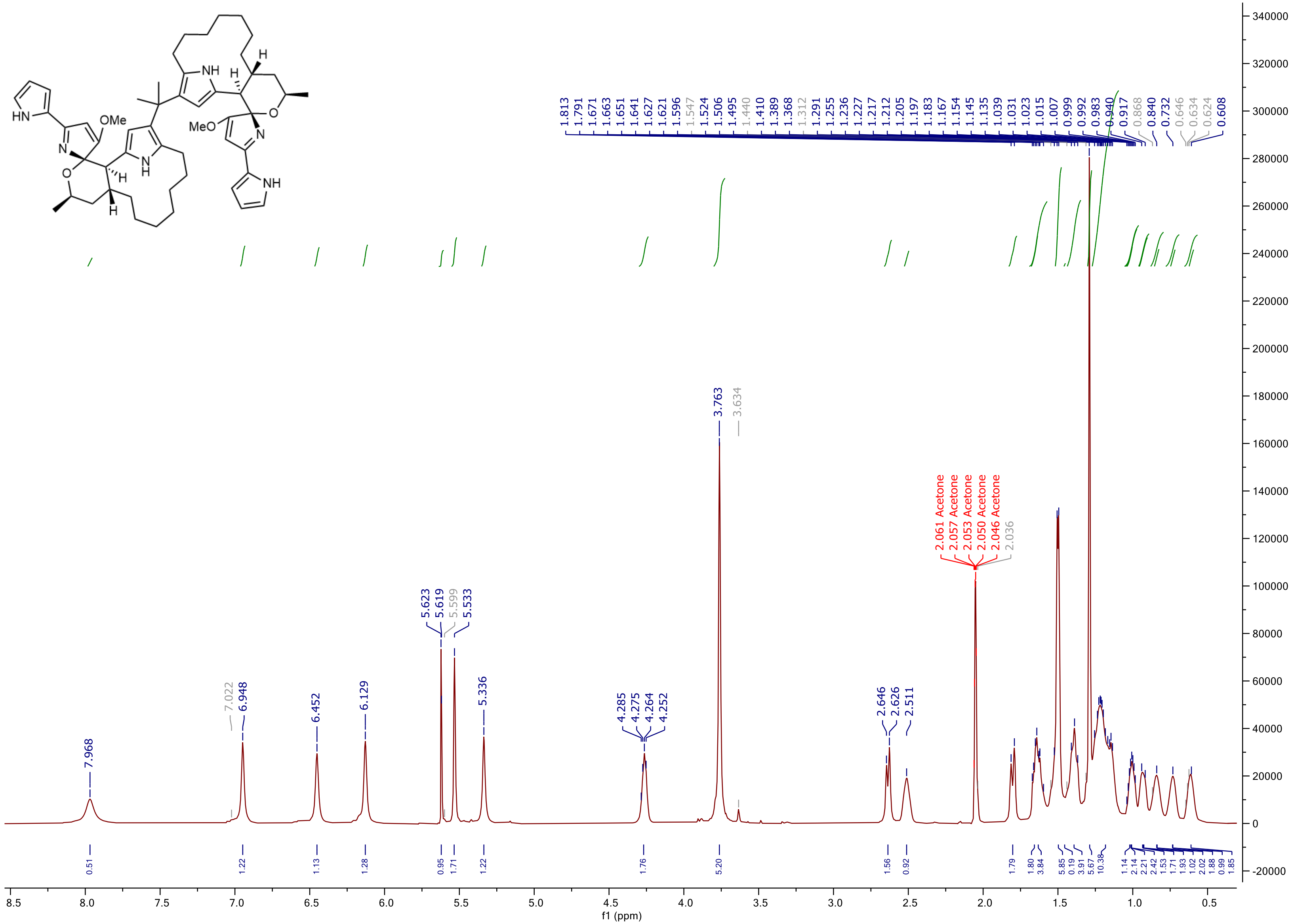

NMR Spectrum 10. ^{1}H NMR Spectrum (600 MHz) of gem-dimethyl-bridged premarineosin A (**4**) in acetone-D$_6$.

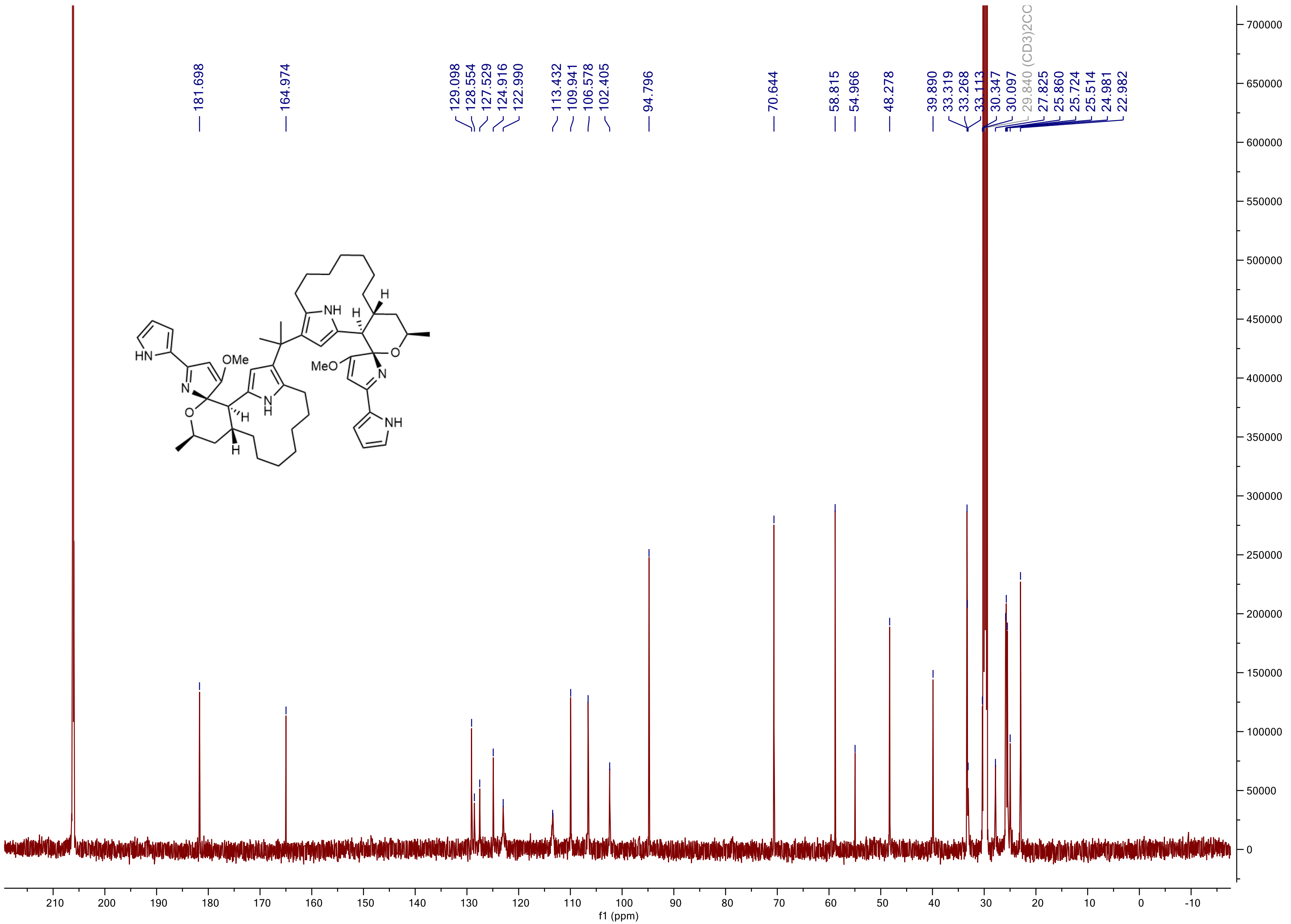

NMR Spectrum 11. ^{13}C NMR Spectrum (151 MHz) of gem-dimethyl-bridged premarineosin A (**4**) in acetone-D_6.

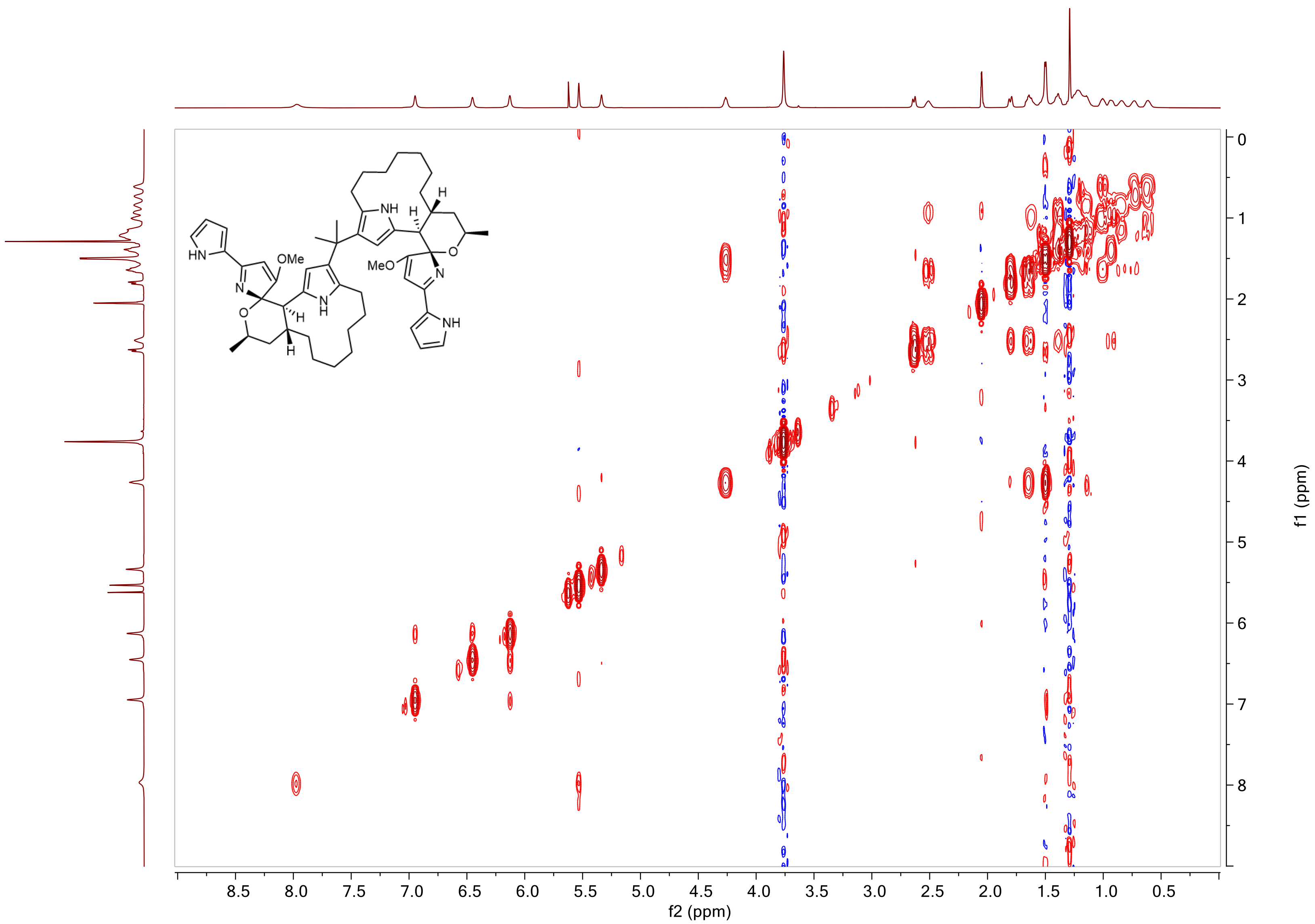

NMR Spectrum 12. ^{1}H-^{1}H COSY NMR Spectrum of gem-dimethyl-bridged premarineosin A (**4**) in acetone-D_6.

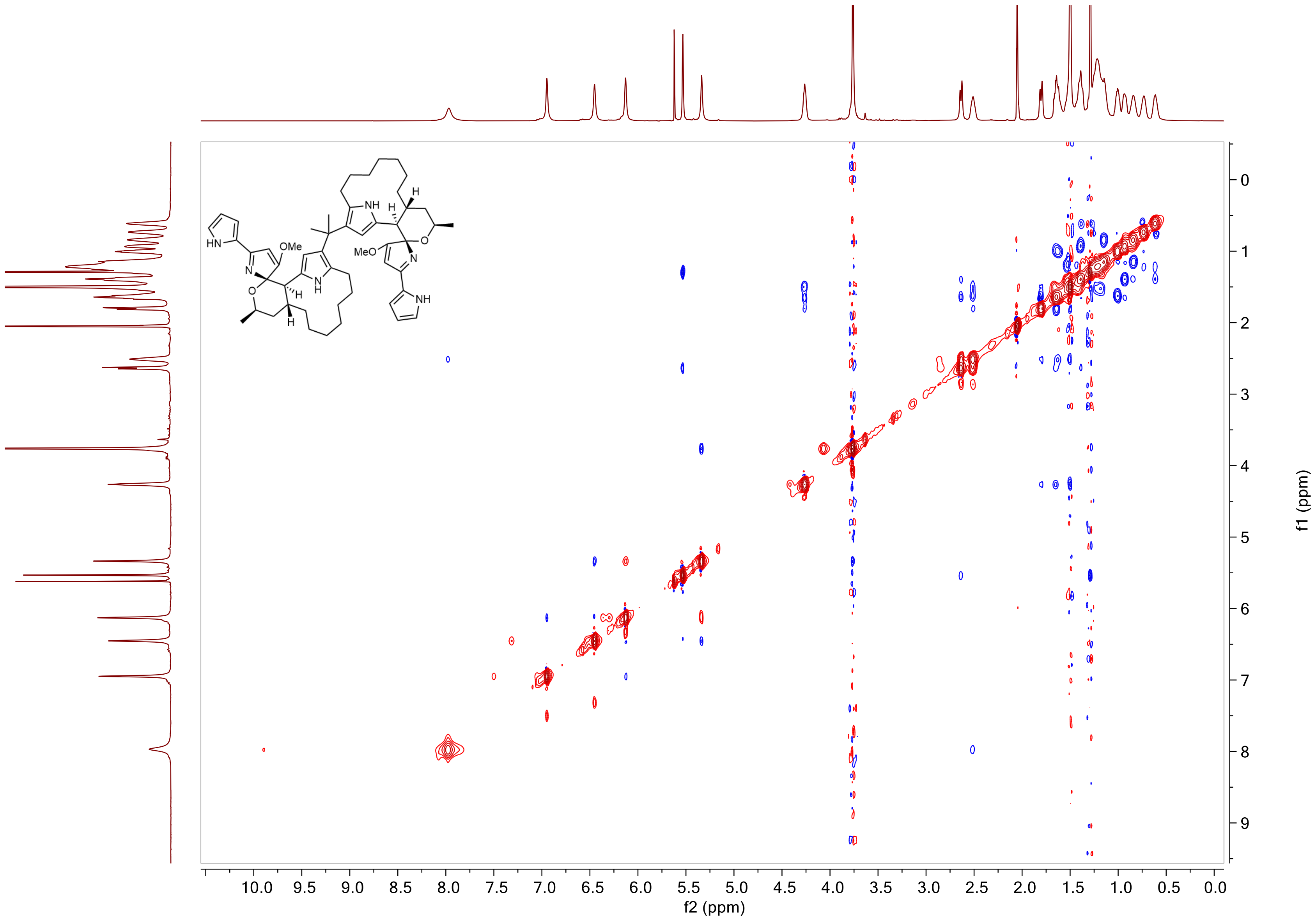

NMR Spectrum 13. ^{1}H-^{1}H NOESY NMR Spectrum of gem-dimethyl-bridged premarineosin A (**4**) in acetone-D$_6$.

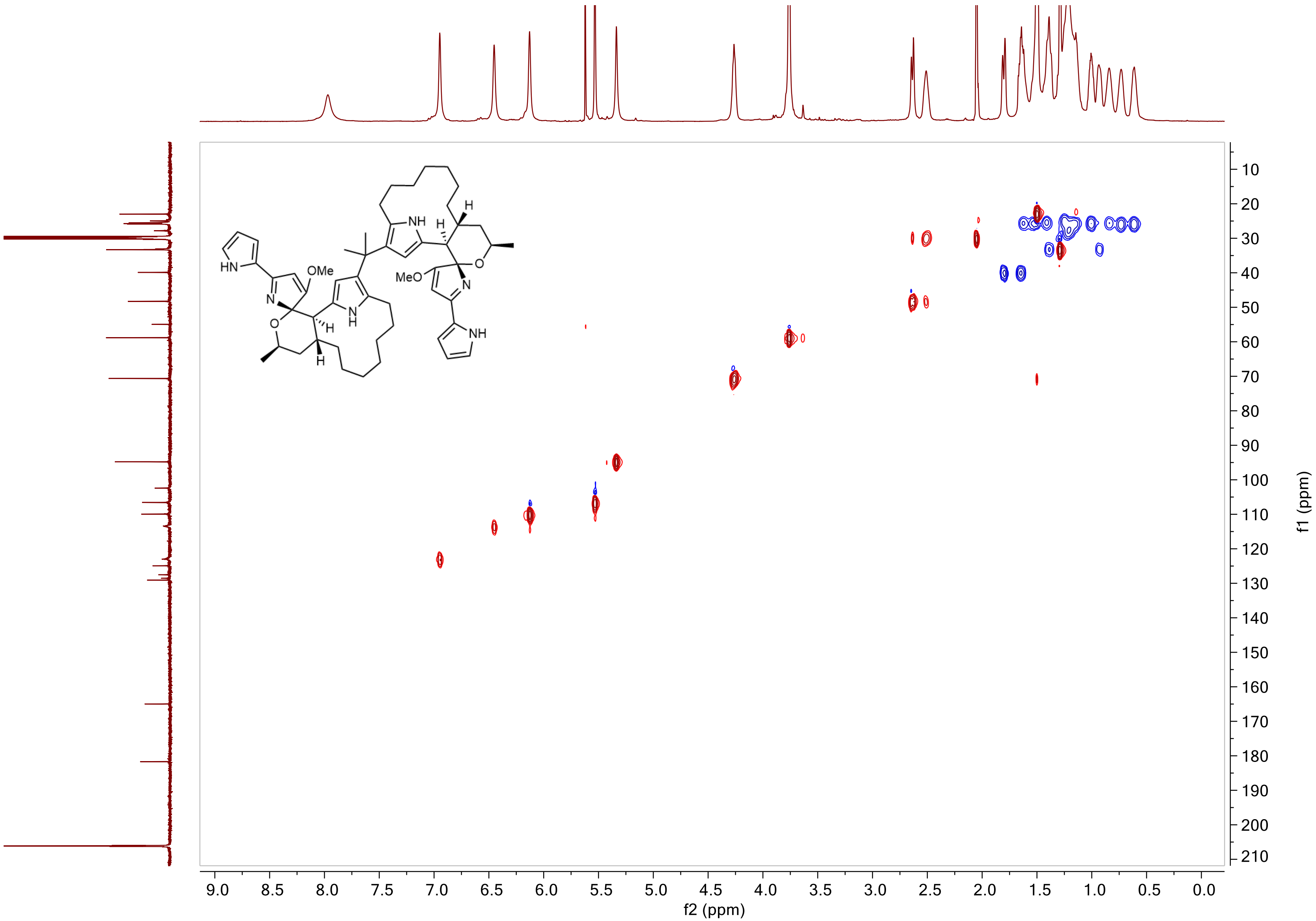

NMR Spectrum 14. ^{1}H-^{13}C HSQC NMR Spectrum of gem-dimethyl-bridged premarineosin A (**4**) in acetone-D_6.

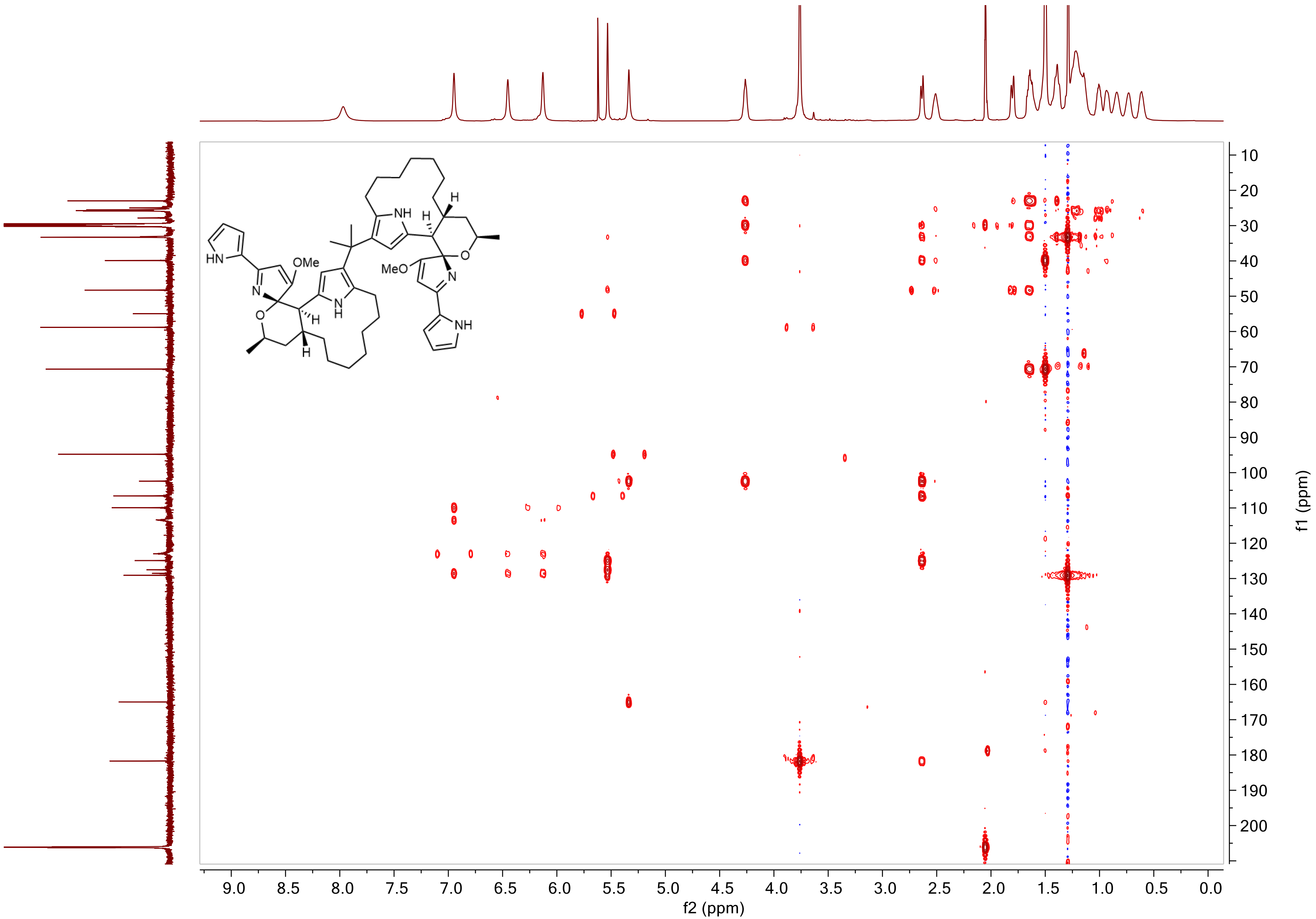

NMR Spectrum 15. ^{1}H-^{13}C HMBC NMR Spectrum of gem-dimethyl-bridged premarineosin A (**4**) in acetone-D_6.

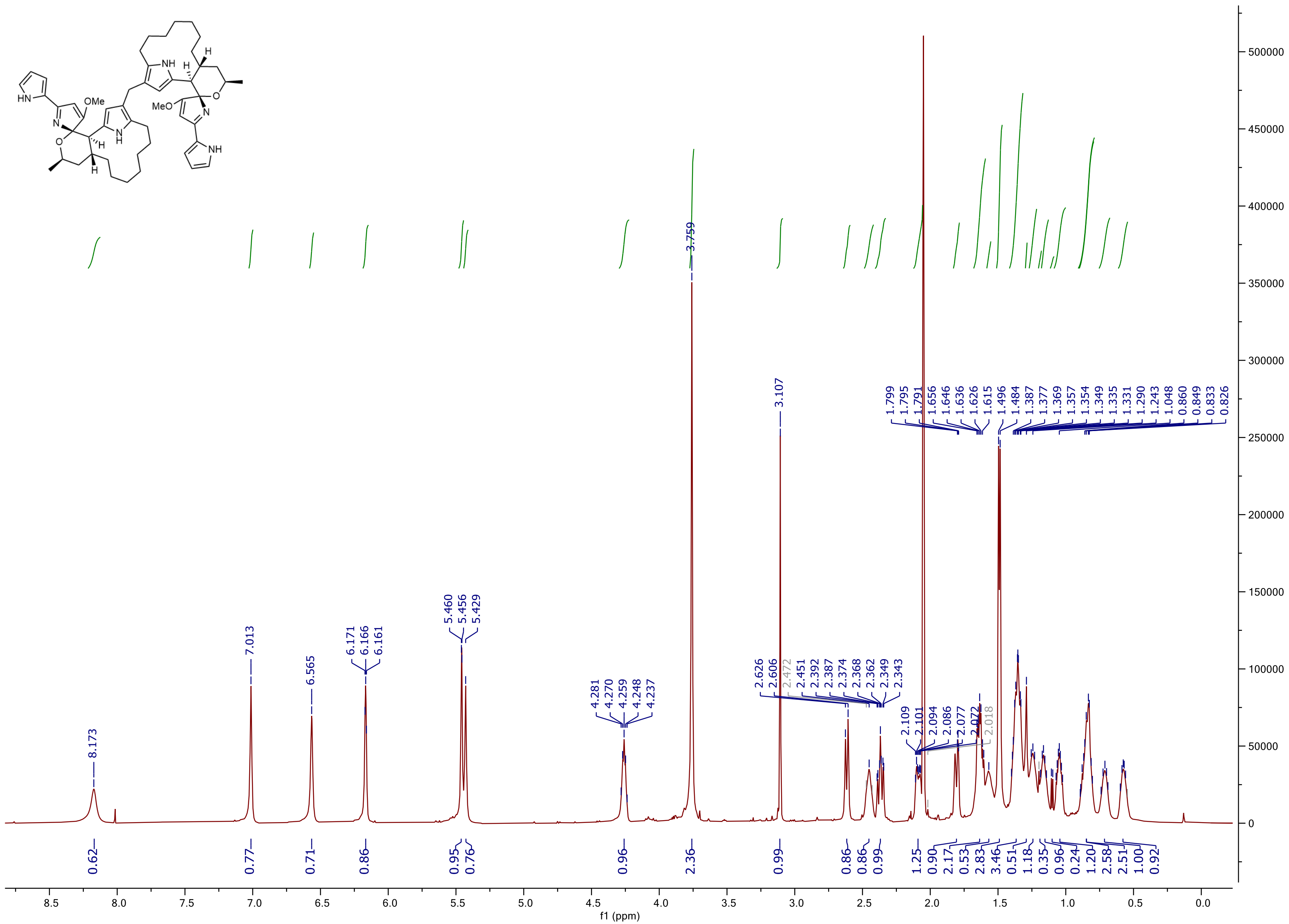

NMR Spectrum 16. ^{1}H NMR Spectrum (600 MHz) of methylene-bridged premarineosin A (**5**) in acetone-D_6.

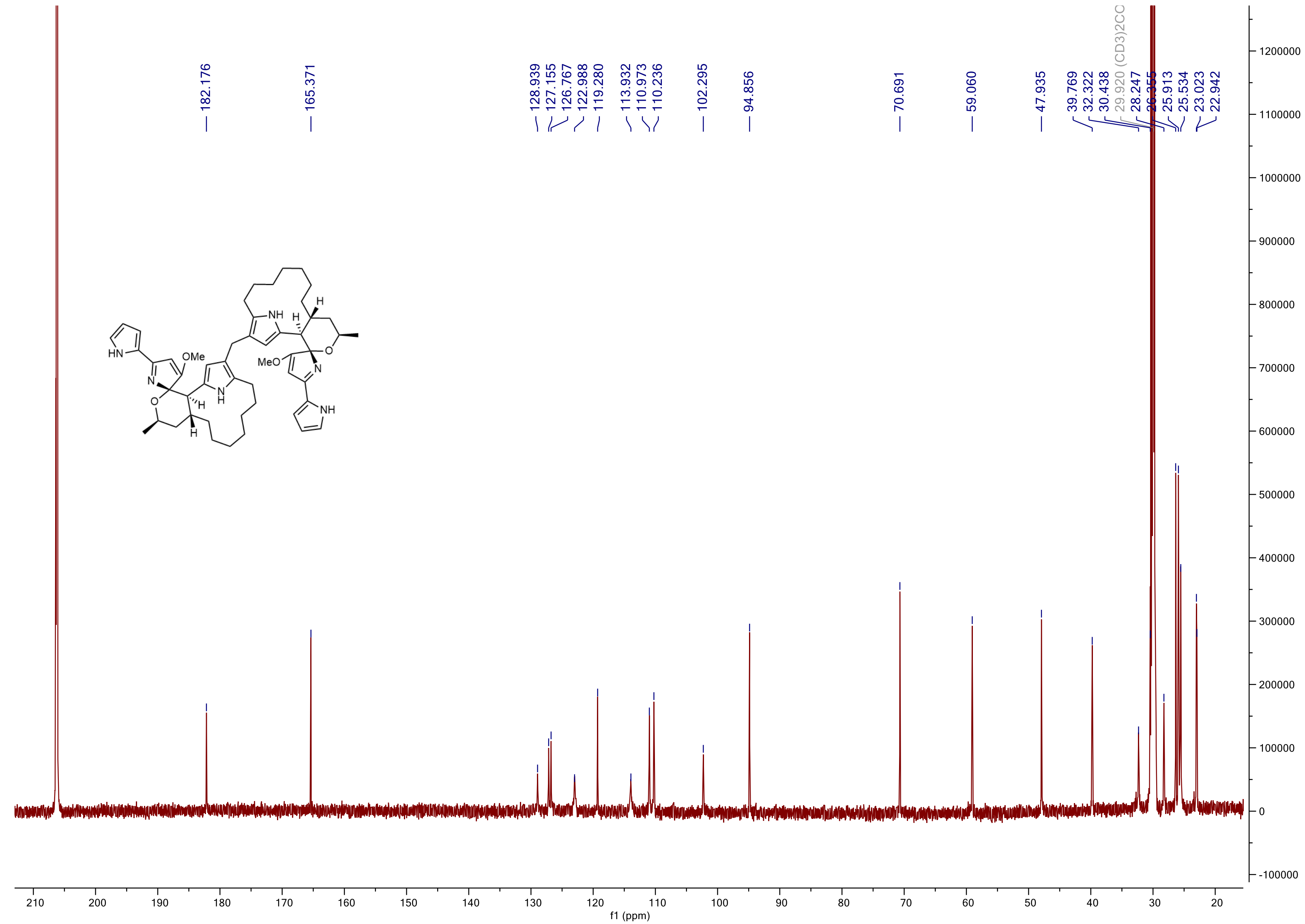

NMR Spectrum 17. ^{13}C NMR Spectrum (151 MHz) of methylene-bridged premarineosin A (**5**) in acetone-D_6.

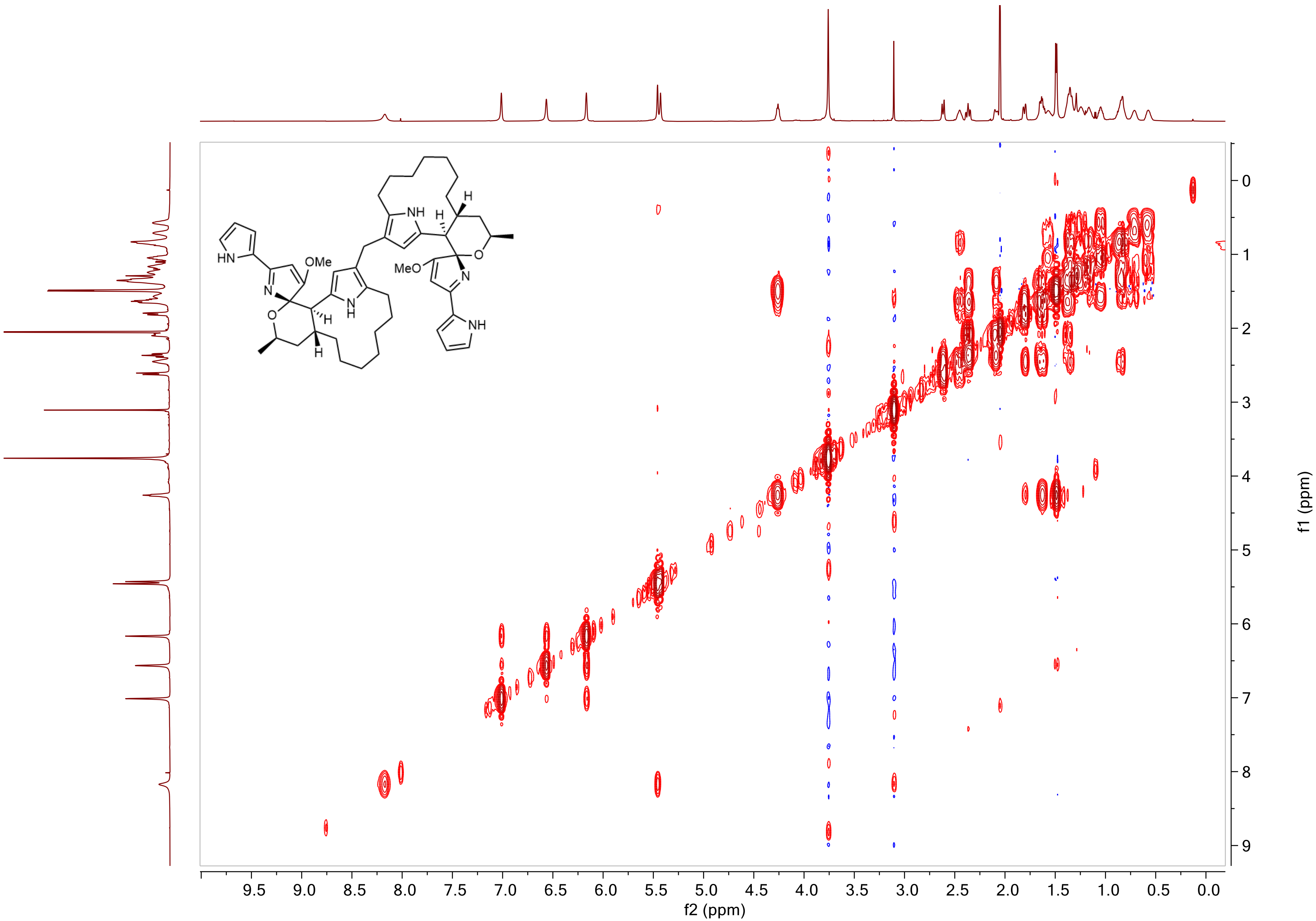

NMR Spectrum 18. ^{1}H-^{1}H COSY NMR Spectrum of methylene-bridged premarineosin A (**5**) in acetone-D6.

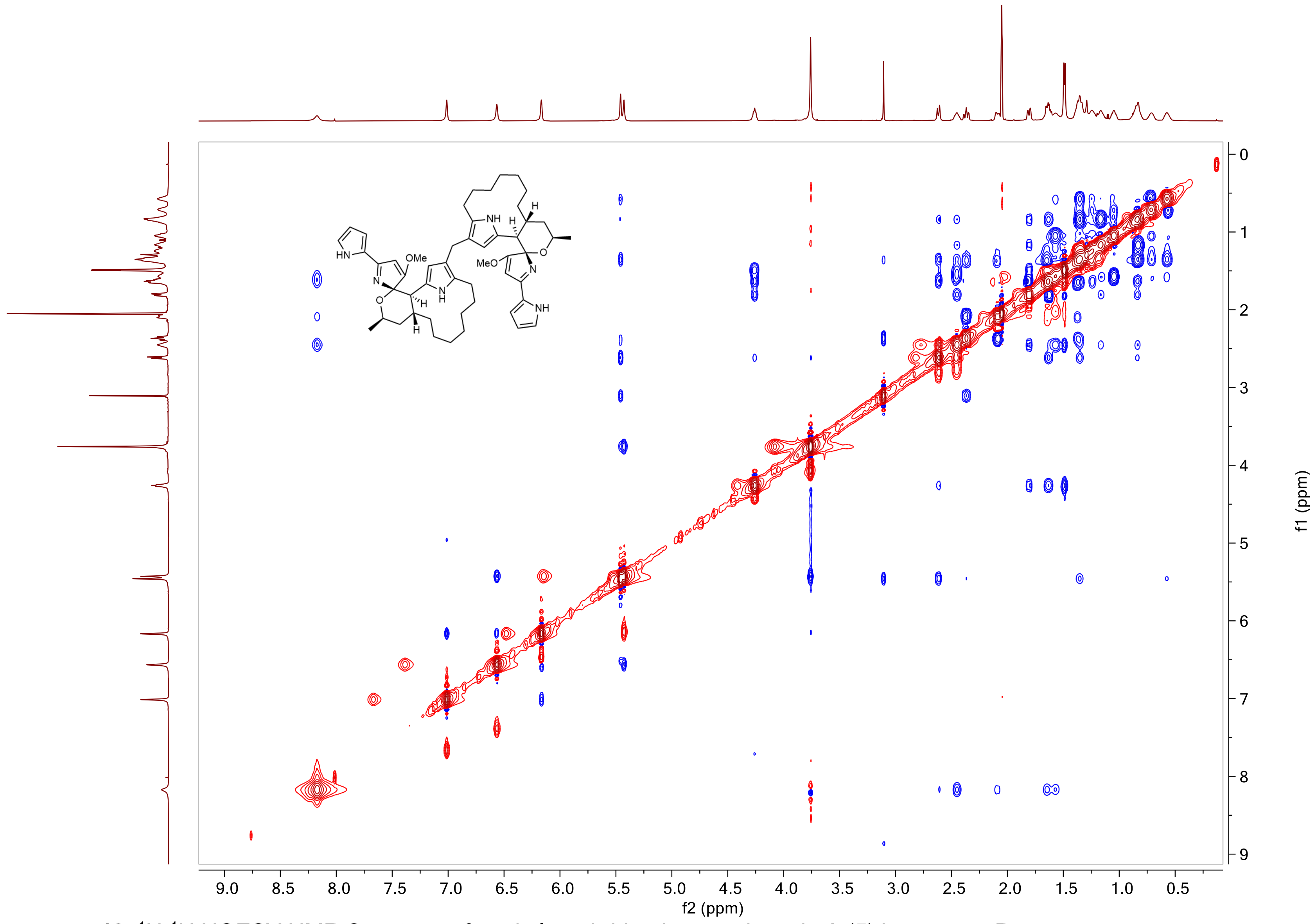

NMR Spectrum 19. ^{1}H-^{1}H NOESY NMR Spectrum of methylene-bridged premarineosin A (**5**) in acetone-D_6.

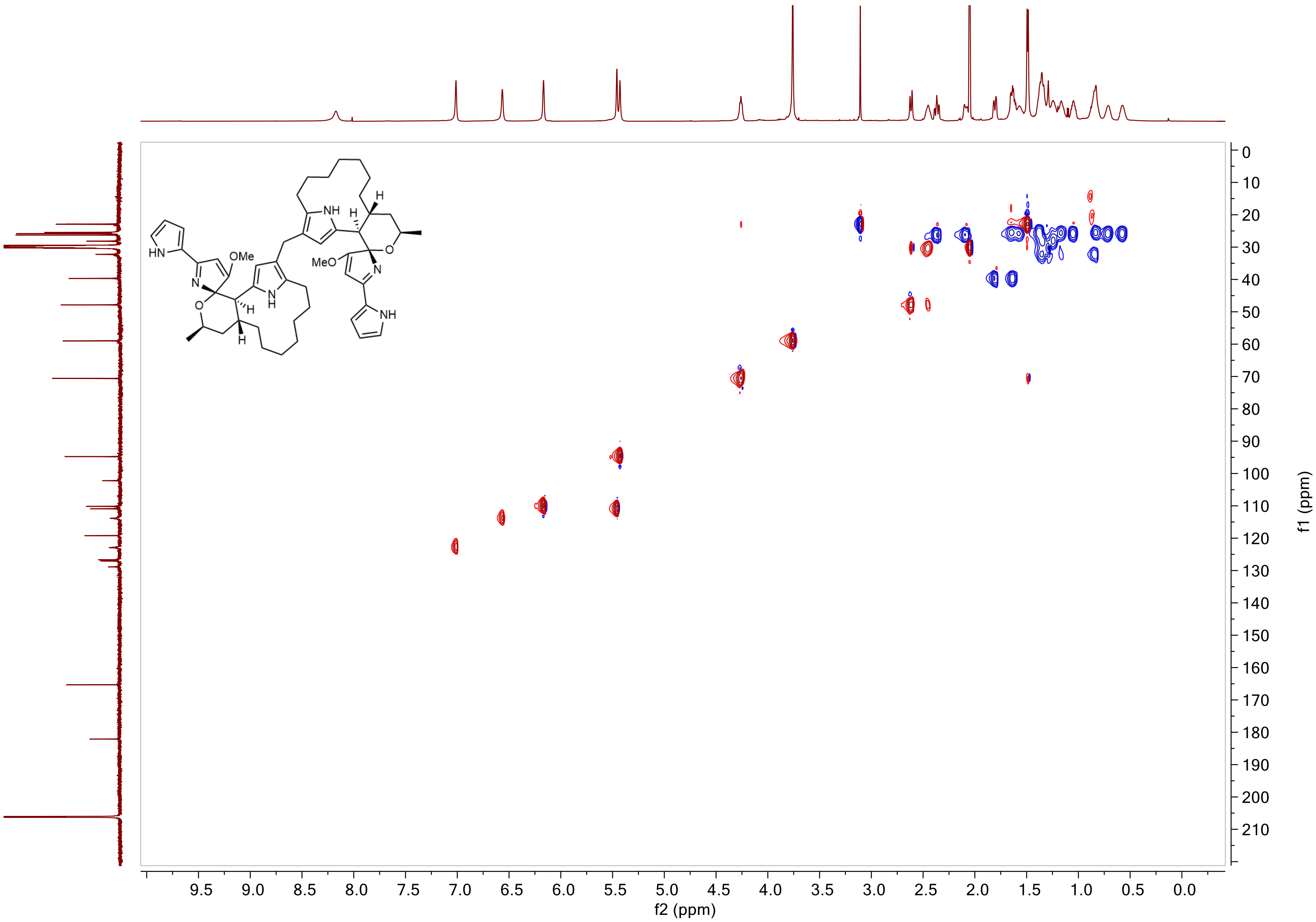

NMR Spectrum 20. ^{1}H-^{13}C HSQC NMR Spectrum of methylene-bridged premarineosin A (**5**) in acetone-D_6.

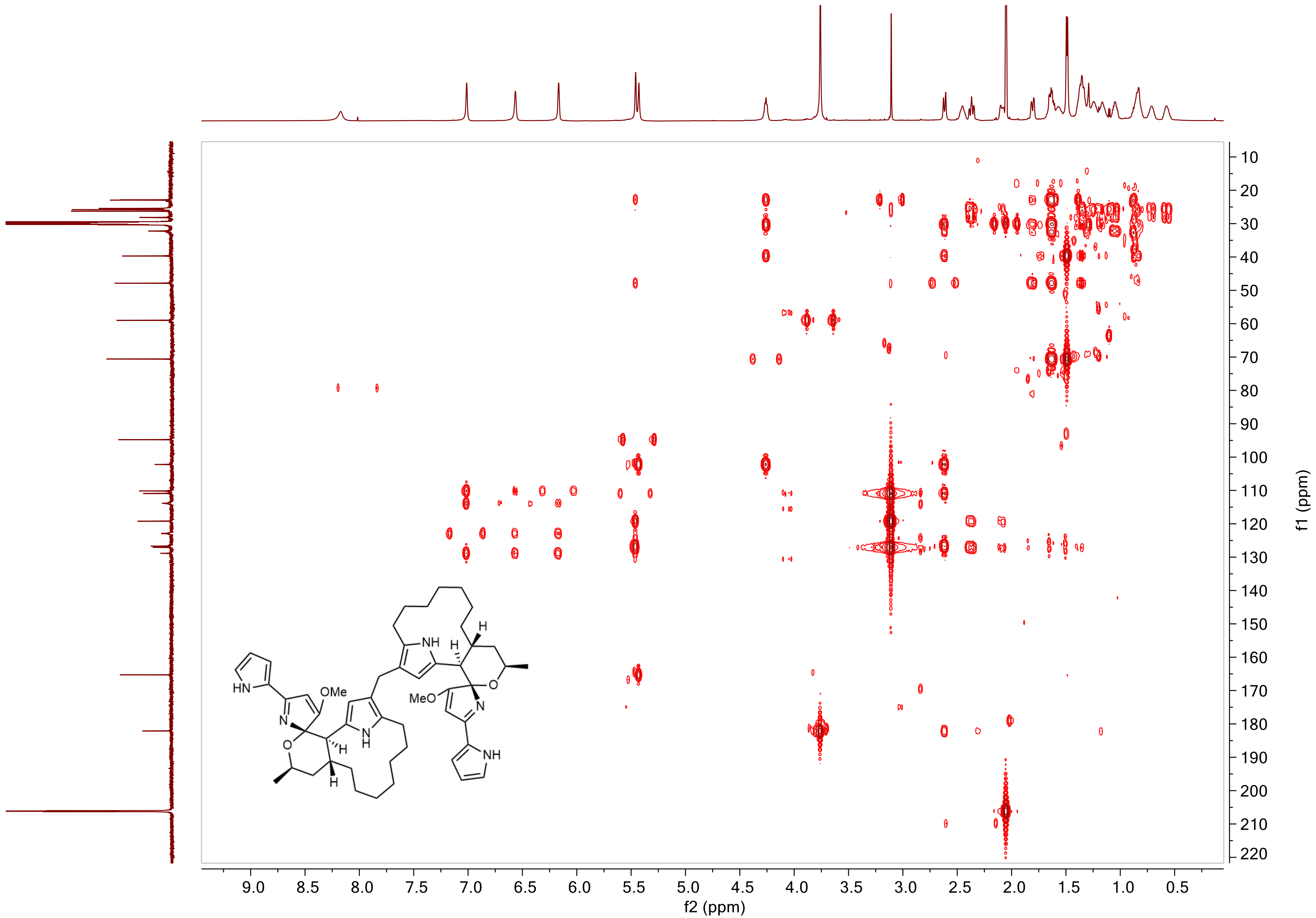

NMR Spectrum 21. ^{1}H-^{13}C HMBC NMR Spectrum of methylene-bridged premarineosin A (**5**) in acetone-D_6.

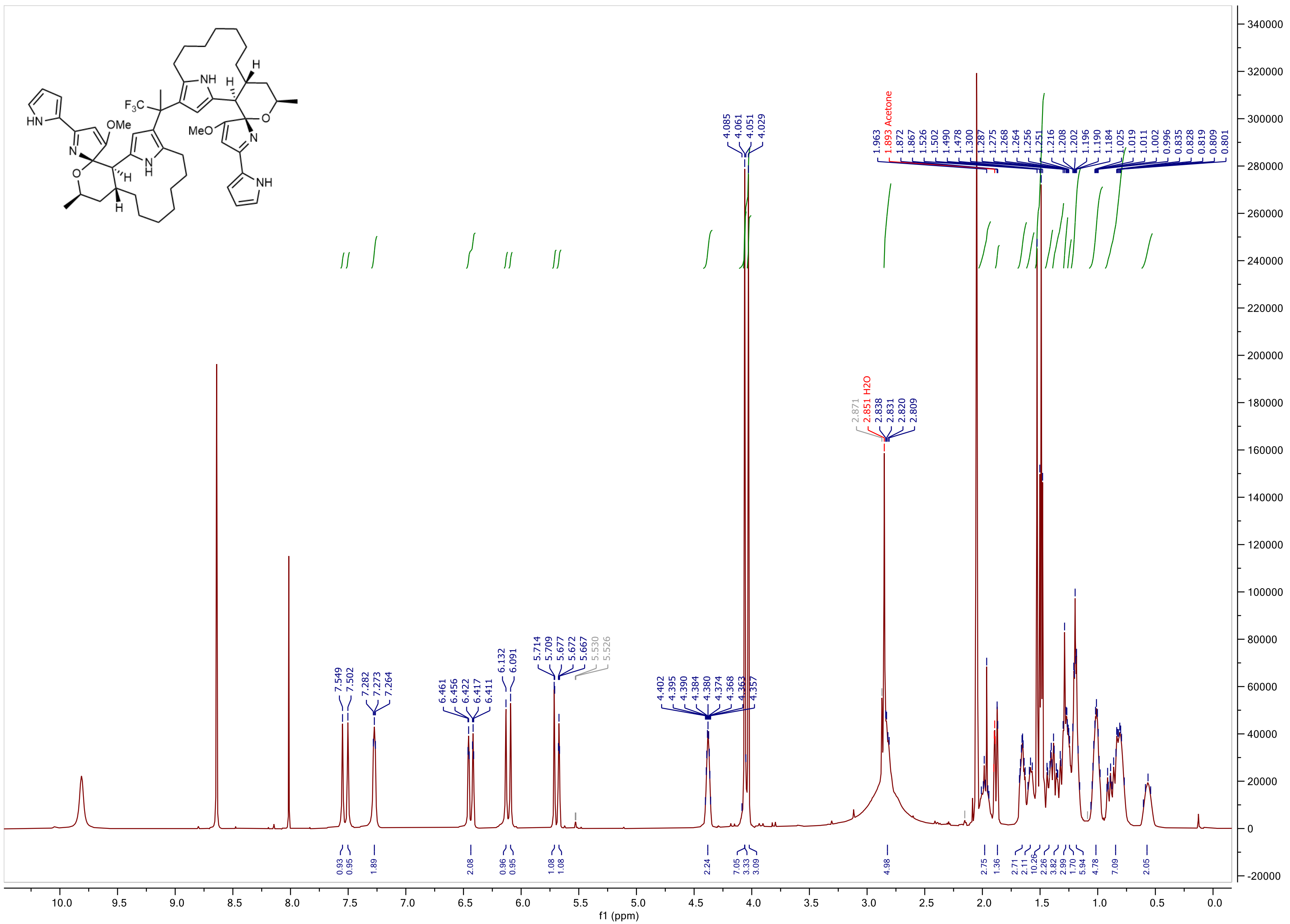

NMR Spectrum 22. ^{1}H NMR Spectrum (600 MHz) of trifluoromethyl-bridged premarineosin A (**6**) in acetone-D$_6$.

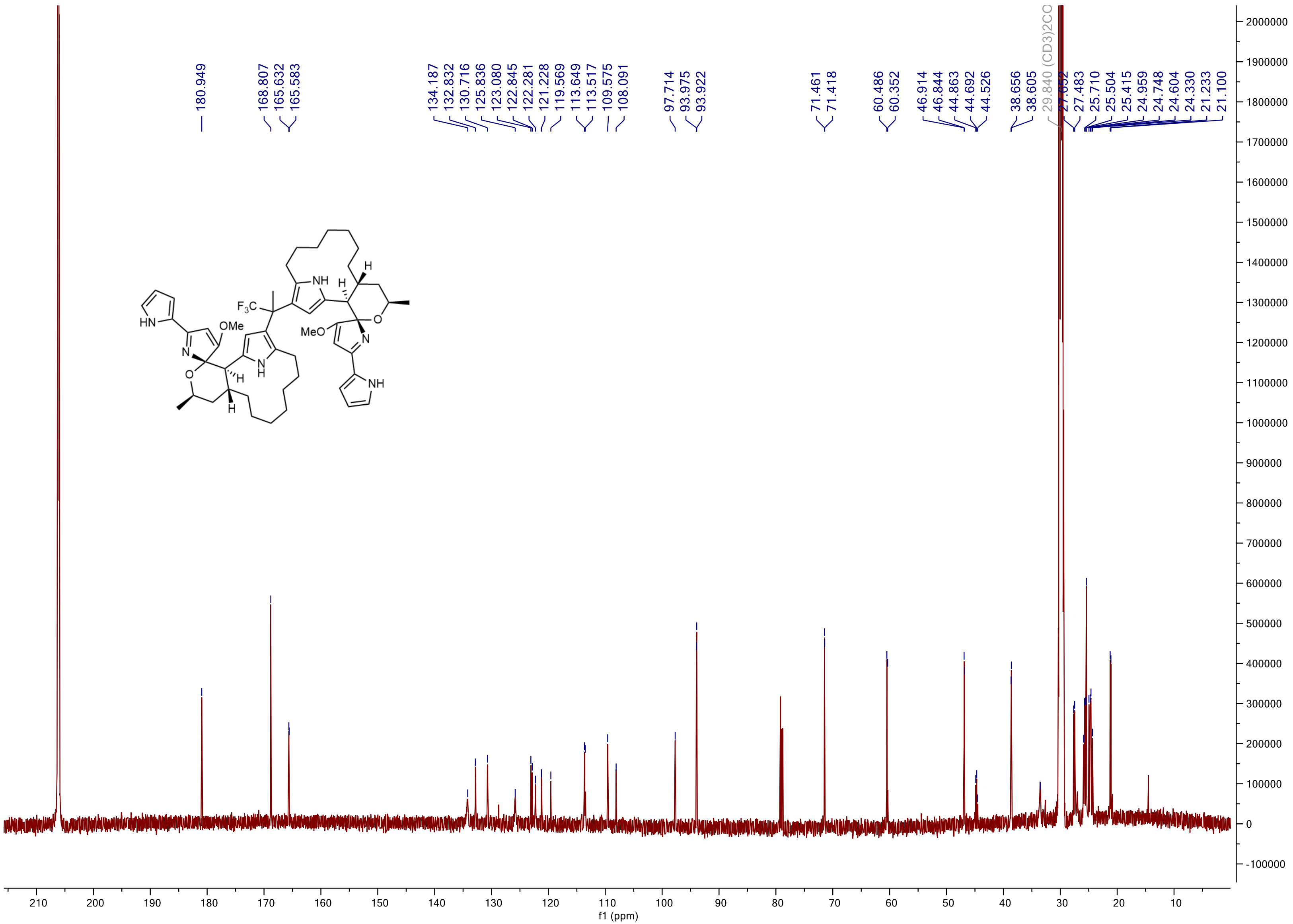

NMR Spectrum 23. ^{13}C NMR Spectrum (151 MHz) of trifluoromethyl-bridged premarineosin A (**6**) in acetone-D_6.

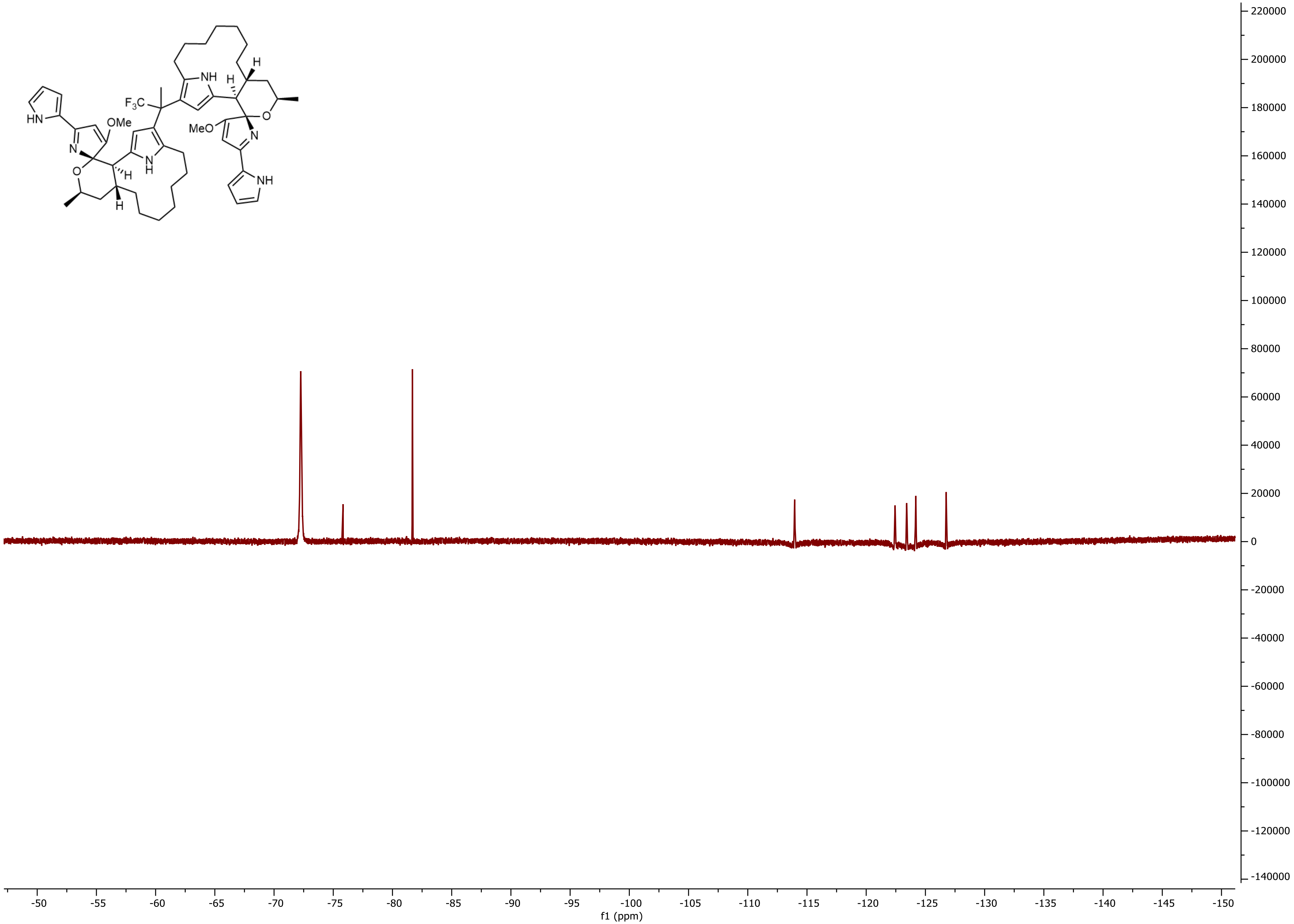

NMR Spectrum 24. ^{19}F NMR Spectrum (563 MHz) of trifluoromethyl-bridged premarineosin A (**6**) in acetone-D_6.

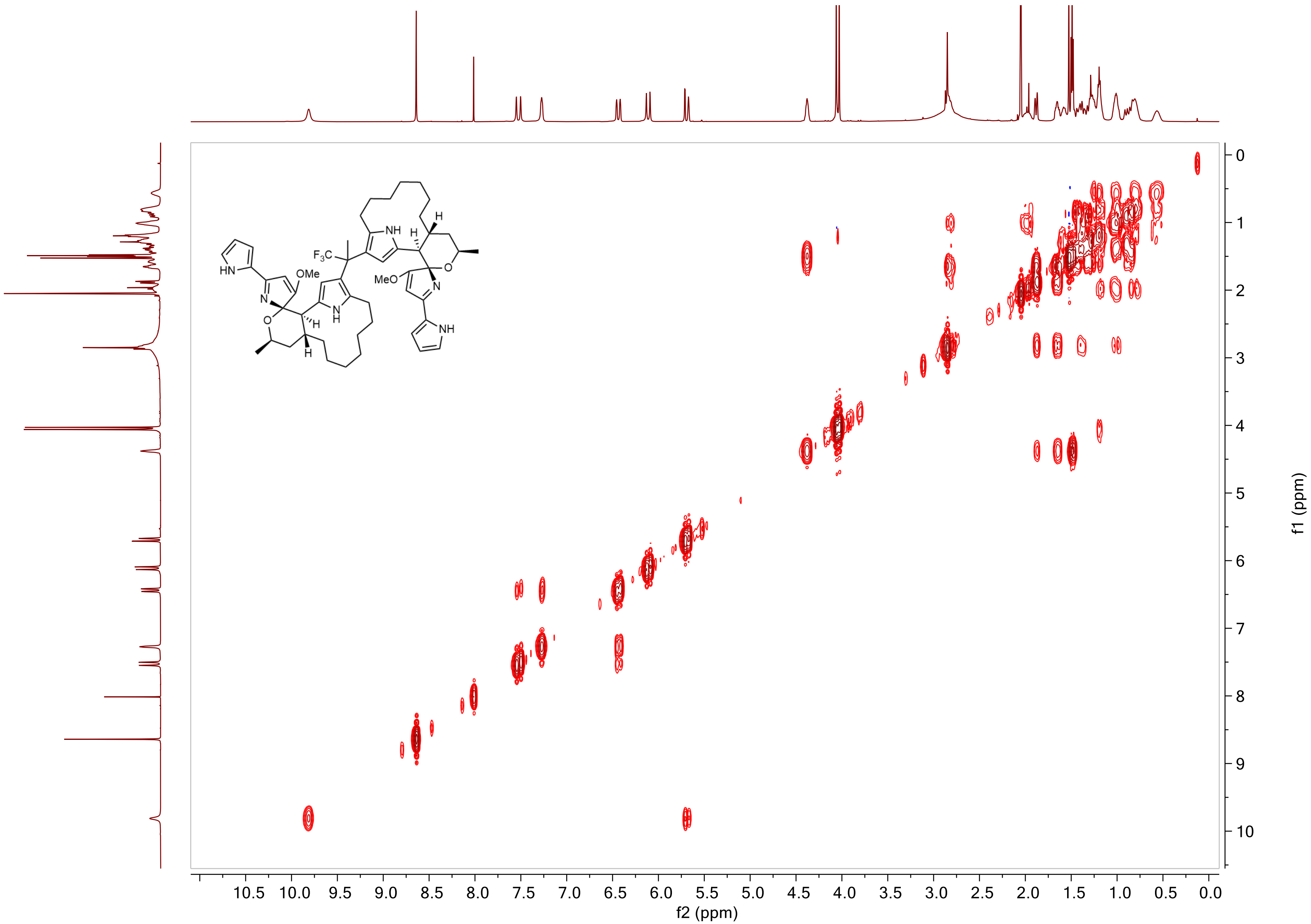

NMR Spectrum 25. ^{1}H-^{1}H COSY NMR Spectrum of trifluoromethyl-bridged premarineosin A (**6**) in acetone-D_6.

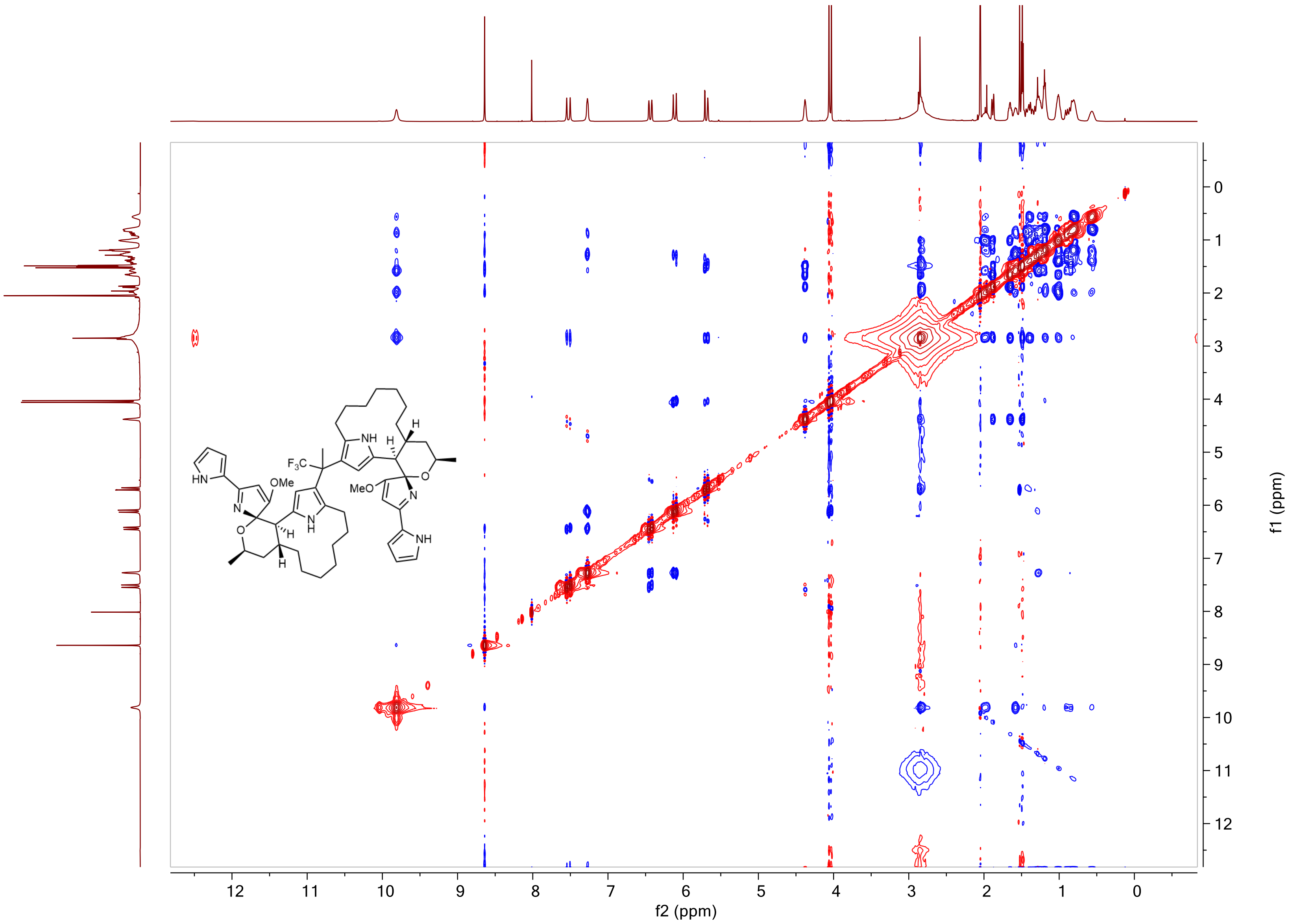

NMR Spectrum 26. ^{1}H-^{1}H NOESY NMR Spectrum of trifluoromethyl-bridged premarineosin A (**6**) in acetone-D$_6$.

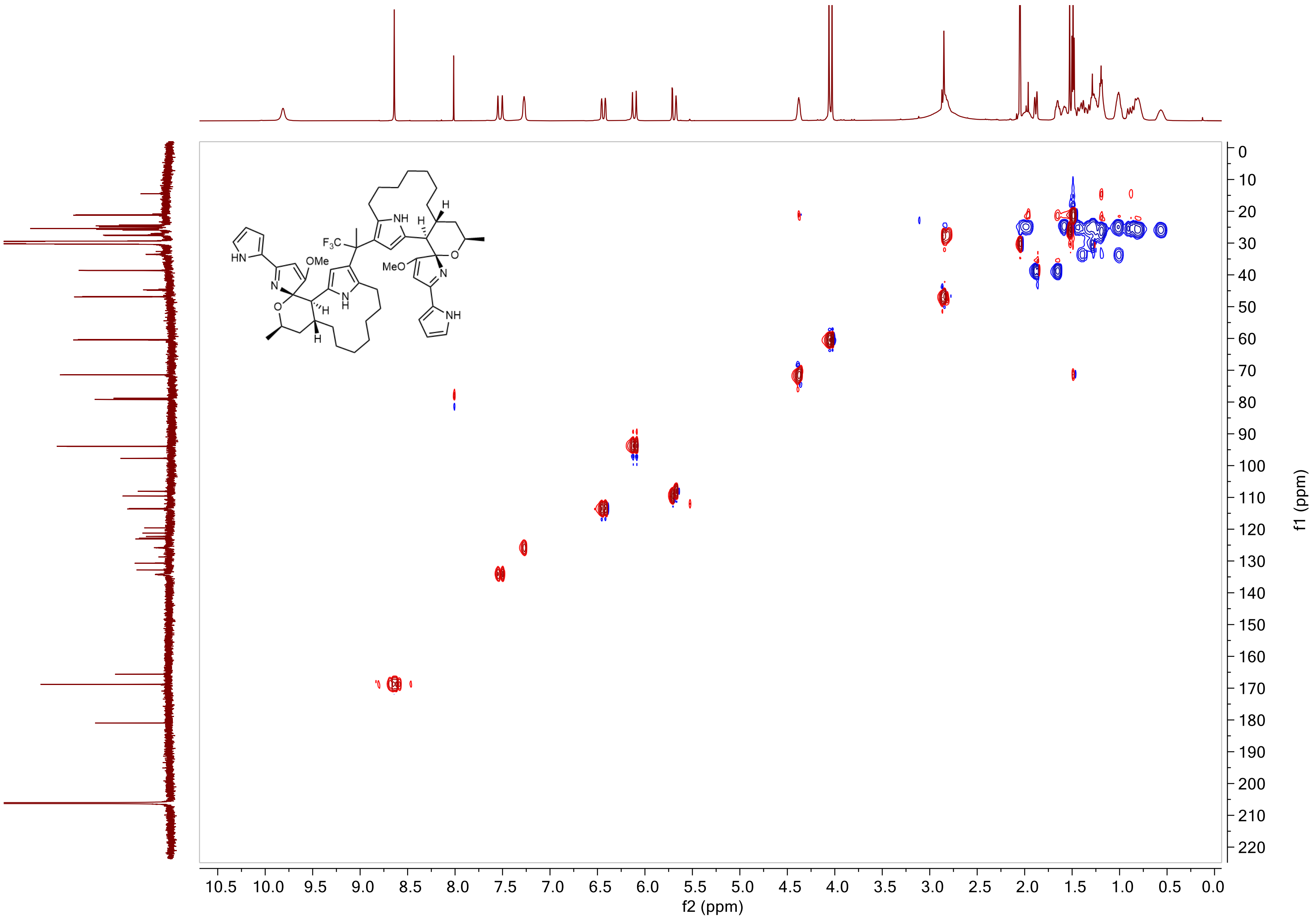

NMR Spectrum 27. ^{1}H-^{13}C HSQC NMR Spectrum of trifluoromethyl-bridged premarineosin A (**6**) in acetone-D$_6$.

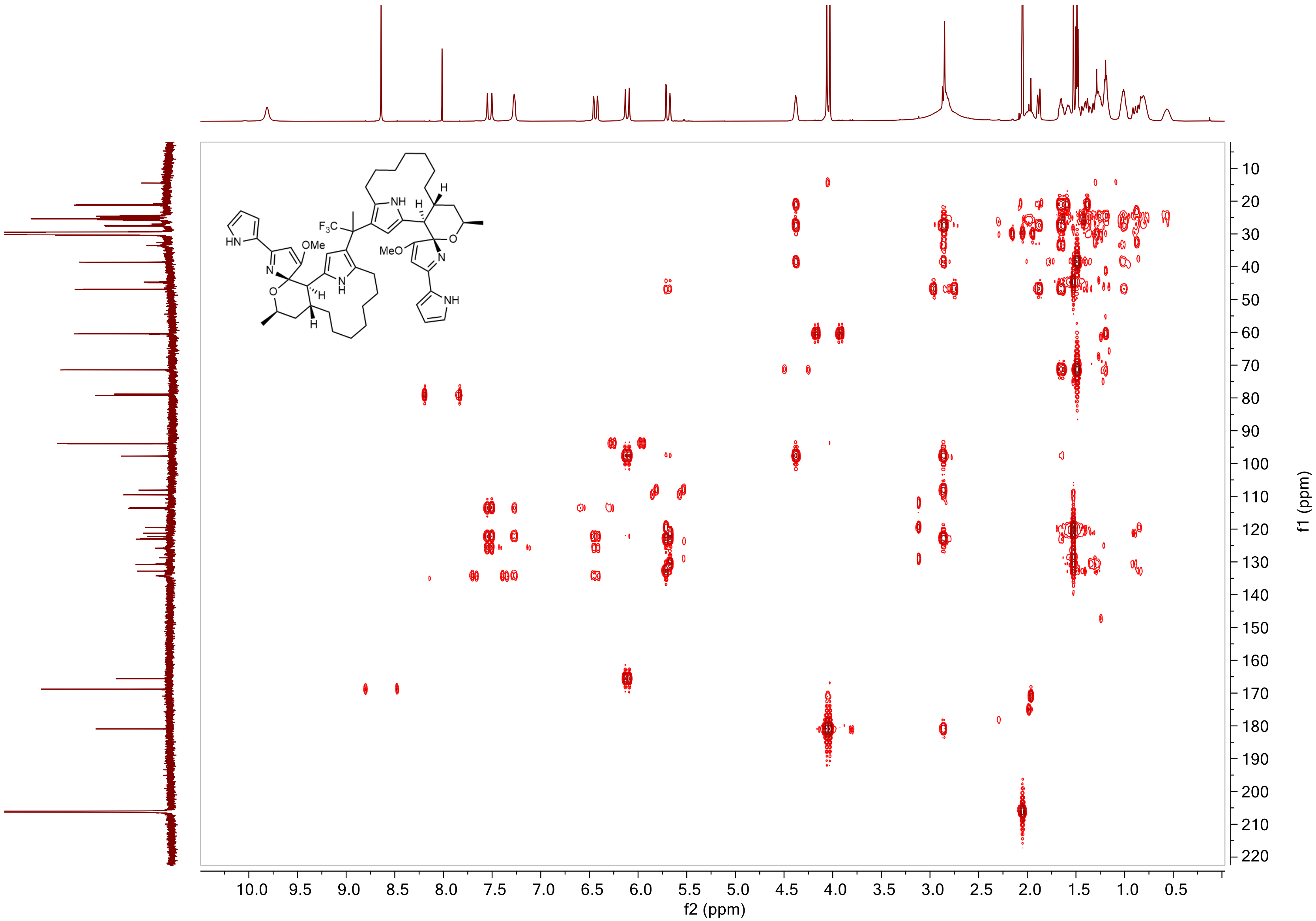

NMR Spectrum 28. ^{1}H-^{13}C HMBC NMR Spectrum of trifluoromethyl-bridged premarineosin A (**6**) in acetone-D_6.

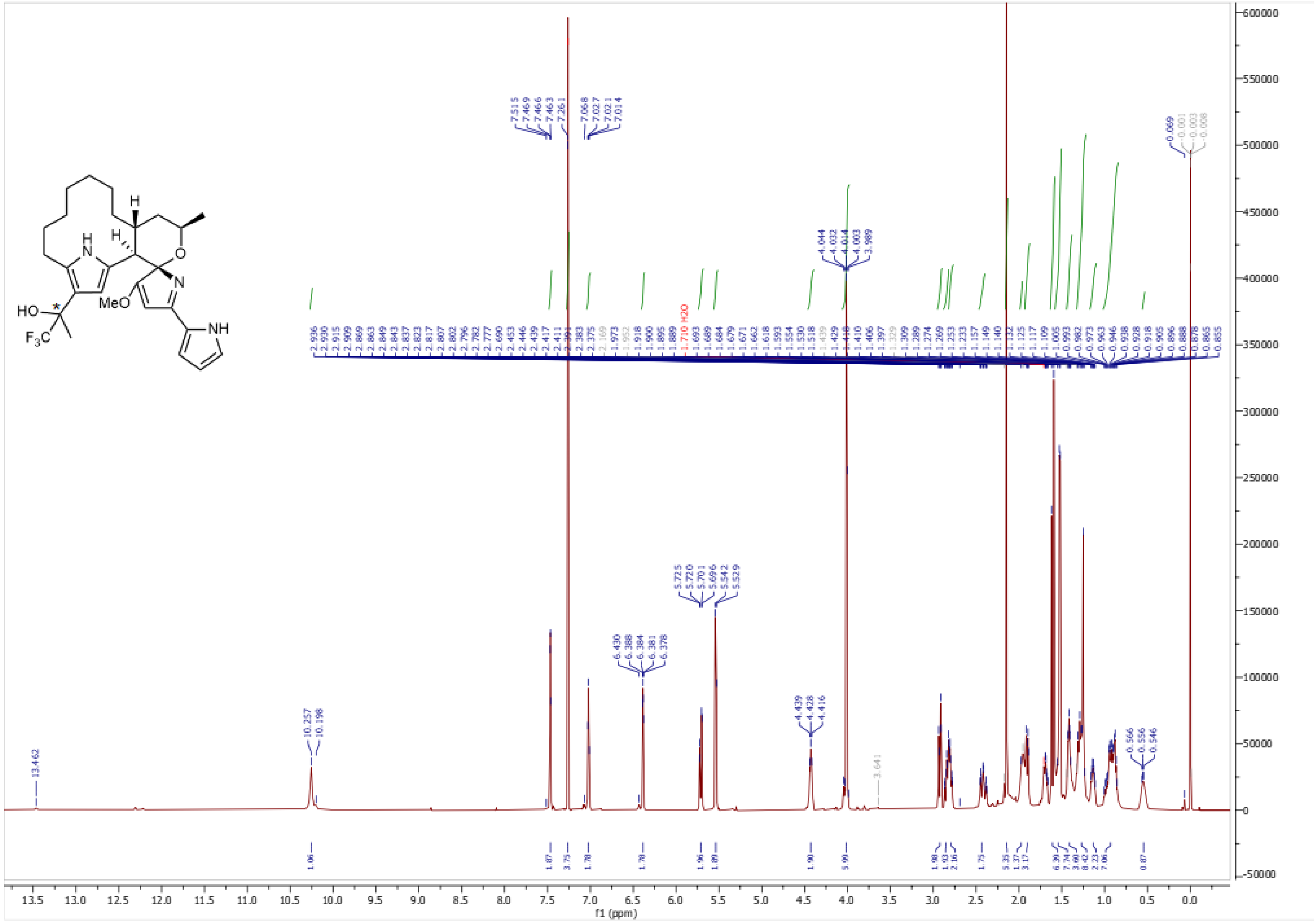

NMR Spectrum 29. ^{1}H NMR Spectrum (600 MHz) of 12-trifluoropropanol premarineosin A (**7**) in chloroform-D.

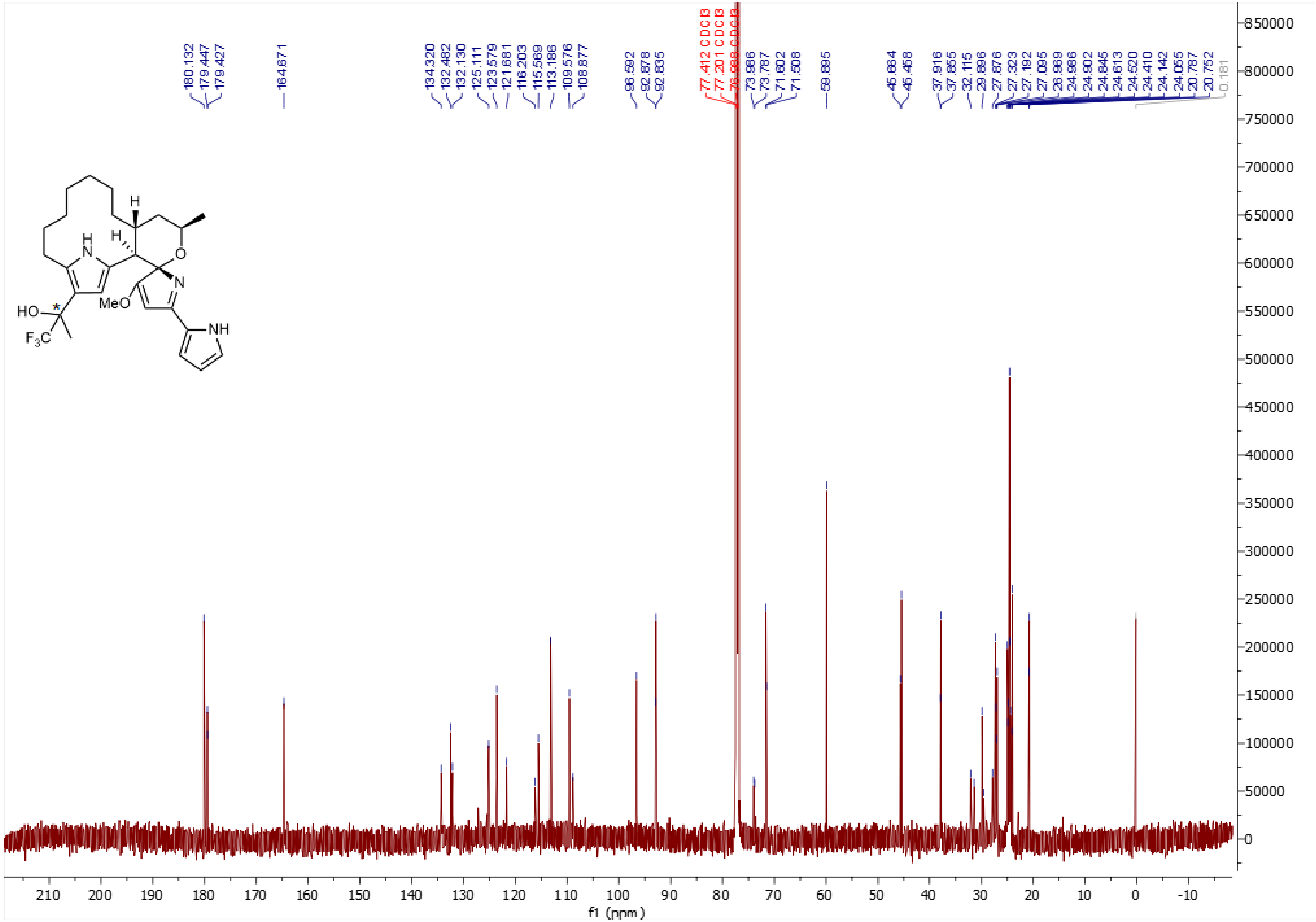

NMR Spectrum 30. ^{13}C NMR (151 MHz) Spectrum of 12-trifluoropropanol premarineosin A (**7**) in chloroform-D.

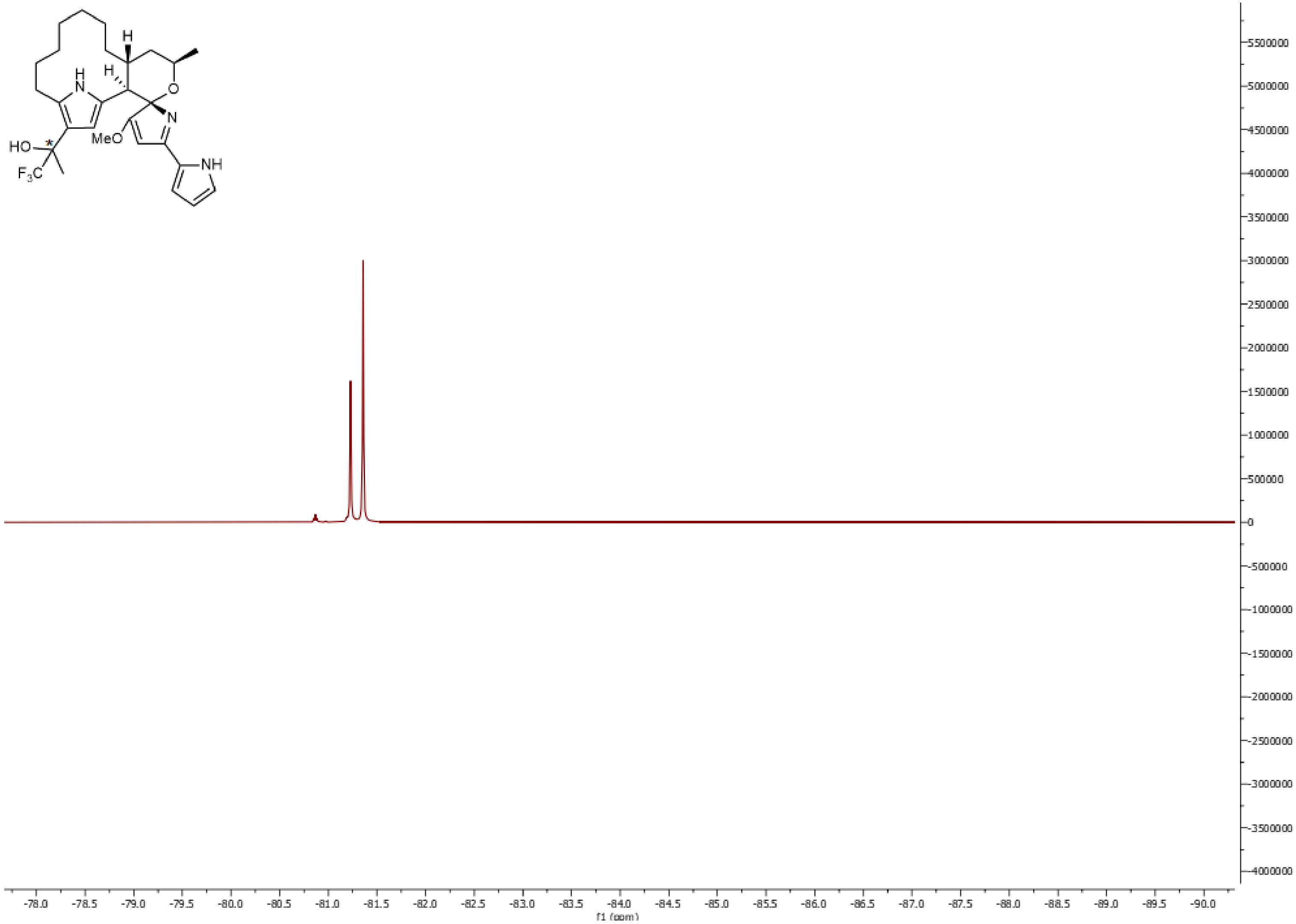

NMR Spectrum 31. ^{19}F NMR Spectrum (563 MHz) of 12-trifluoropropanol premarineosin A (**7**) in acetone-D$_6$.

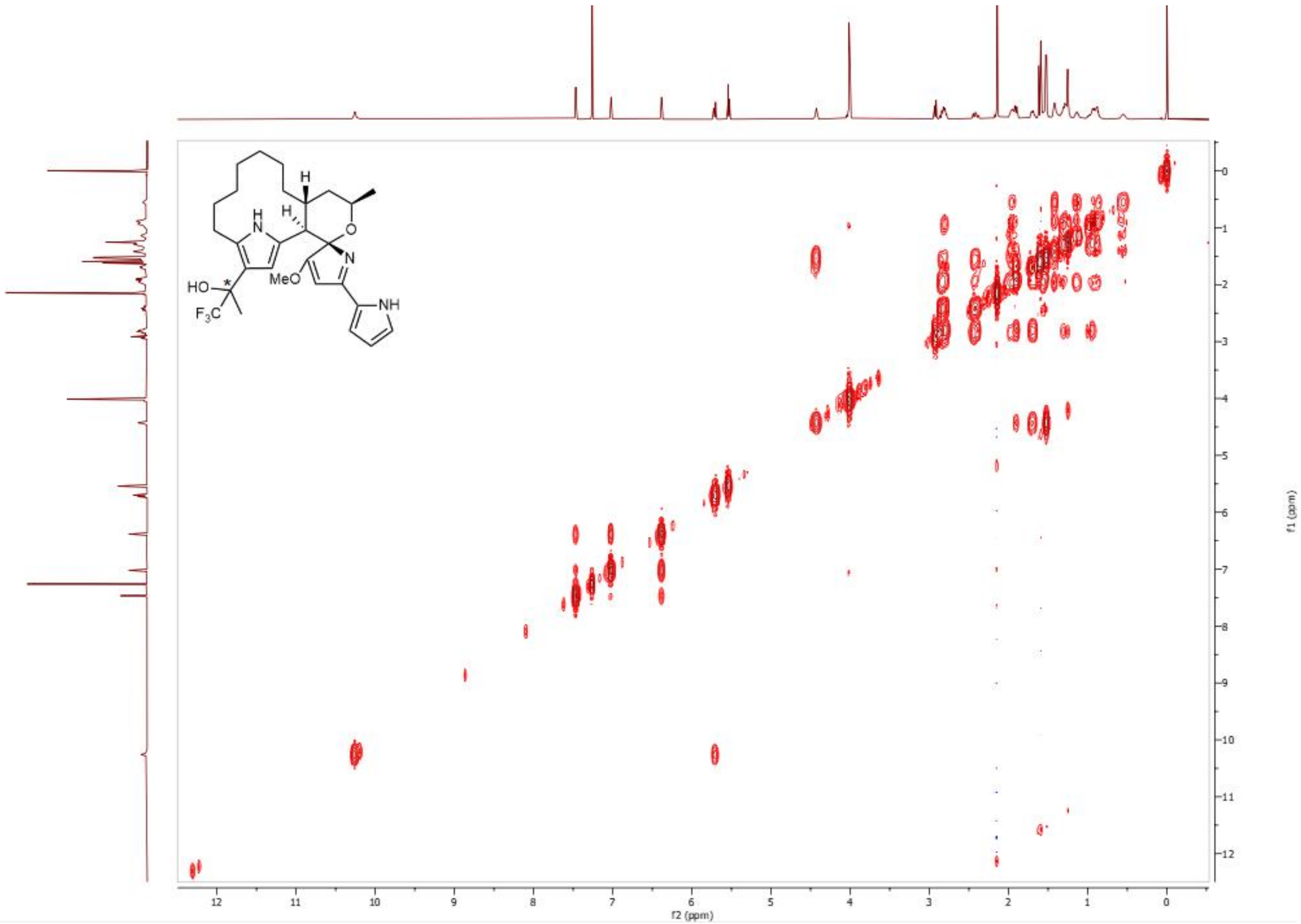

NMR Spectrum 32. ^1H-^1H COSY NMR Spectrum of 12-trifluoropropanol premarineosin A (**7**) in chloroform-D.

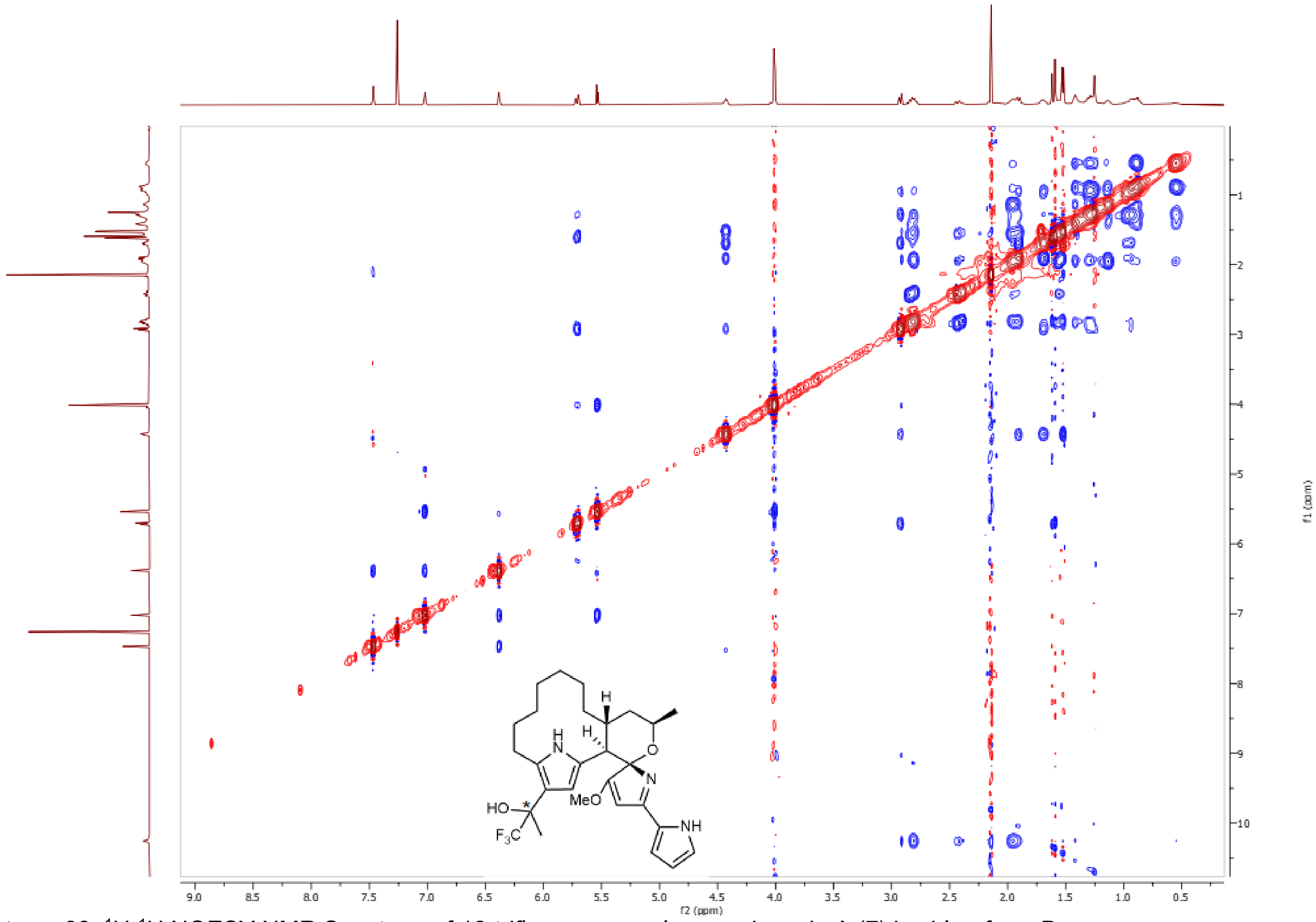

NMR Spectrum 33. ^{1}H-^{1}H NOESY NMR Spectrum of 12-trifluoropropanol premarineosin A (**7**) in chloroform-D.

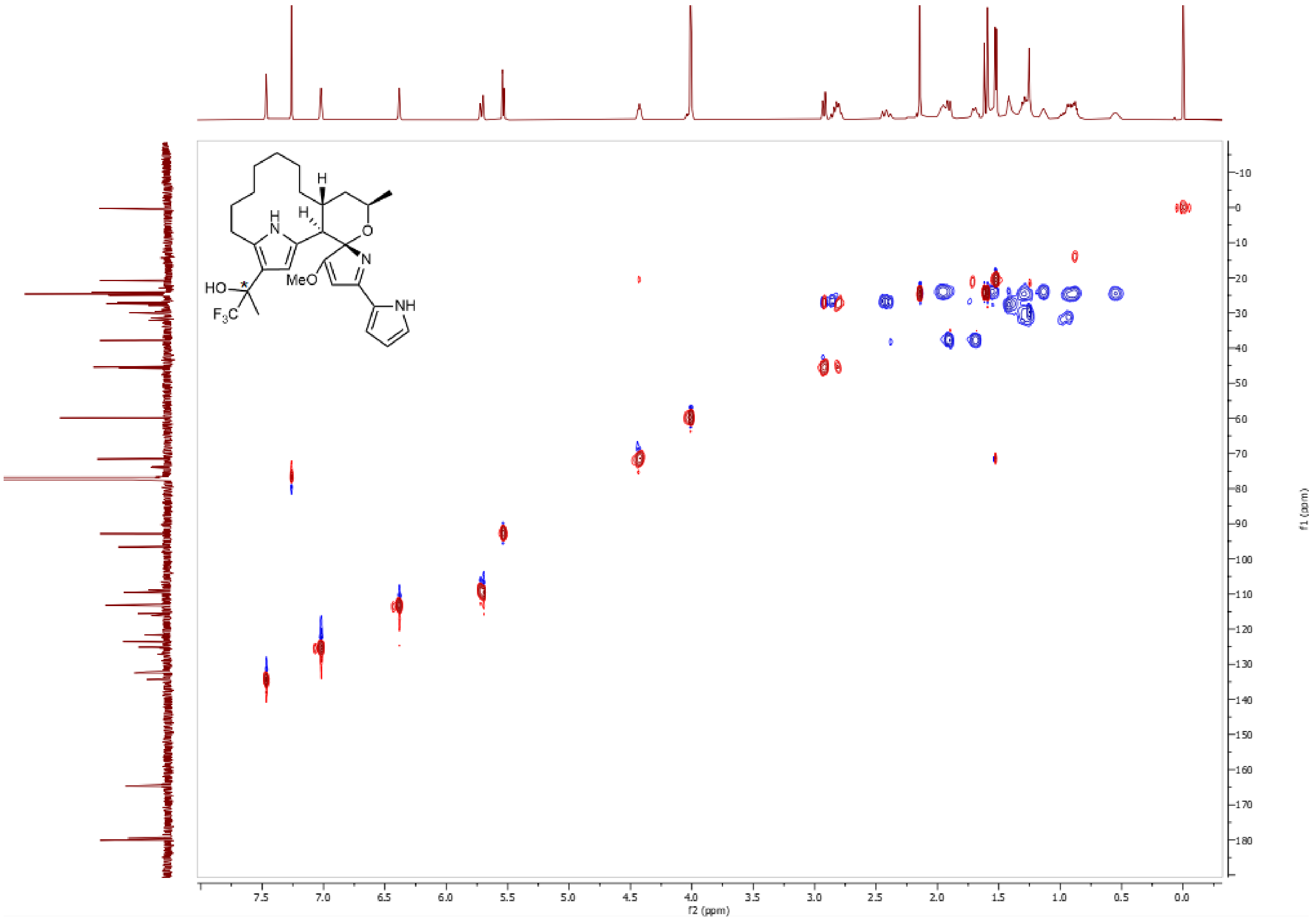

NMR Spectrum 34. ^{1}H-^{13}C HSQC NMR Spectrum of 12-trifluoropropanol premarineosin A (**7**) in chloroform-D.

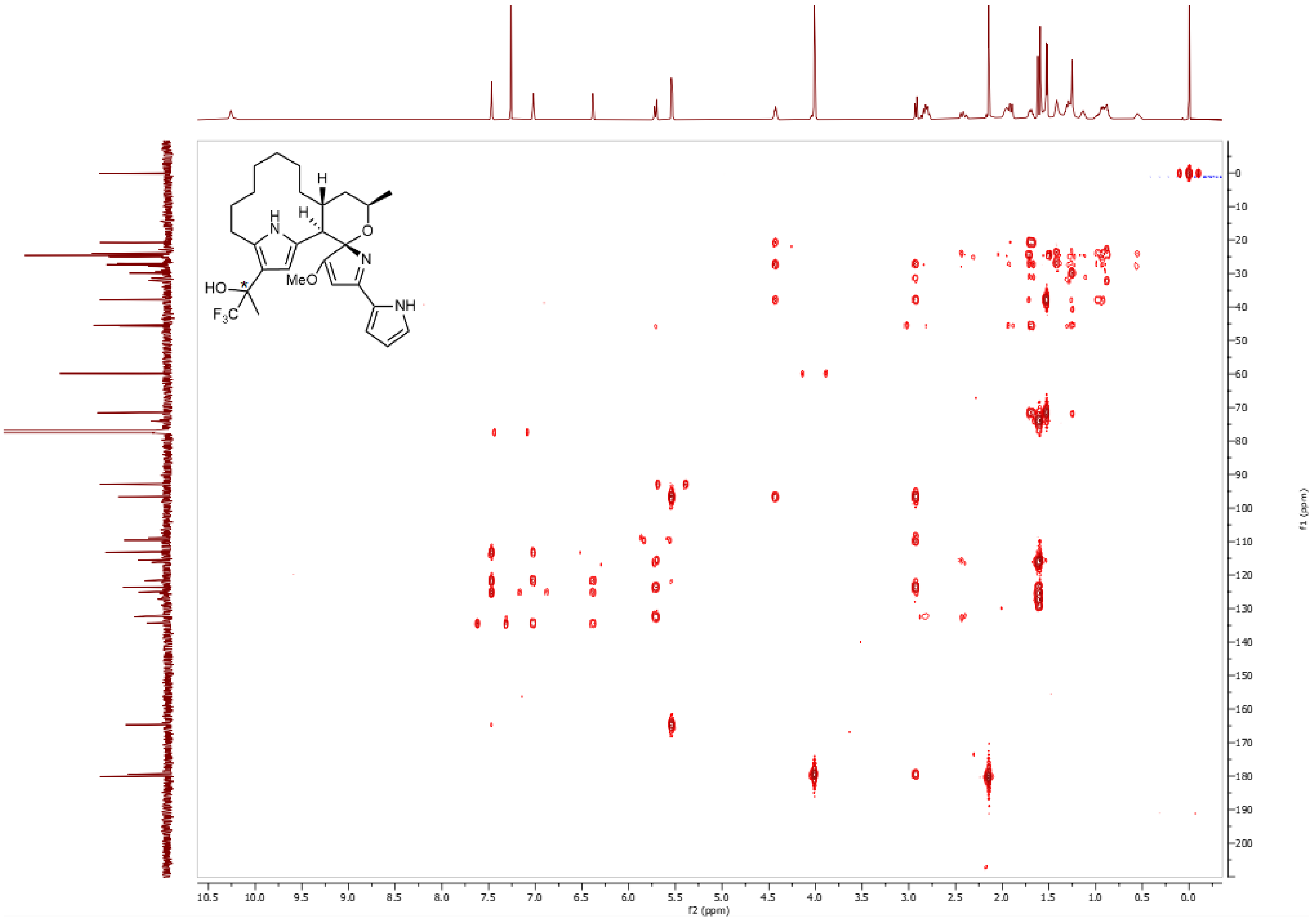

NMR Spectrum 35. ^{1}H-^{13}C HMBC NMR Spectrum of 12-trifluoropropanol premarineosin A (**7**) in chloroform-D.

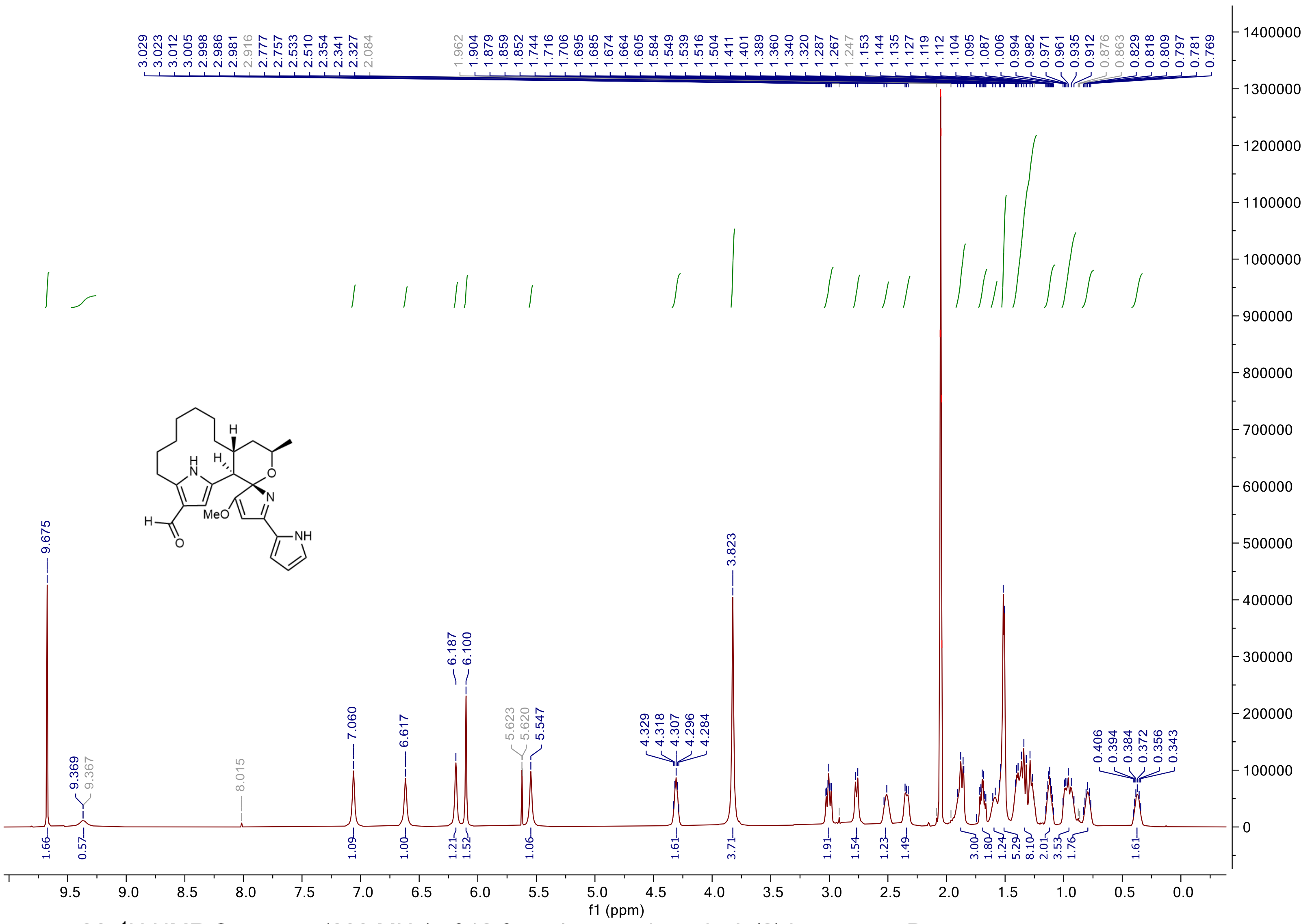

NMR Spectrum 36. ^{1}H NMR Spectrum (600 MHz) of 12-formyl premarineosin A (**8**) in acetone-D$_6$.

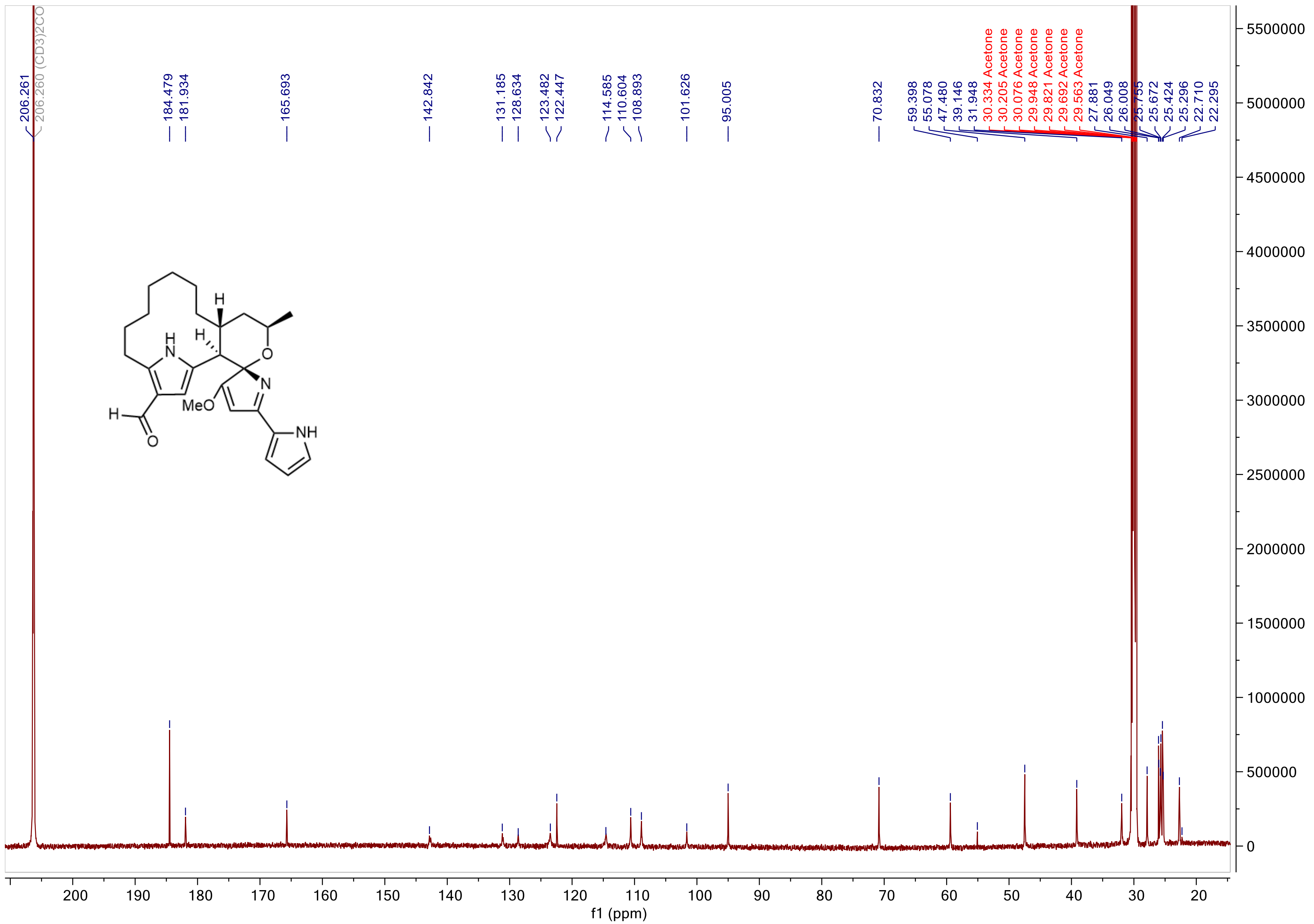

NMR Spectrum 37. ^{13}C NMR Spectrum (151 MHz) of 12-formyl premarineosin A (**8**) in acetone-D_6.

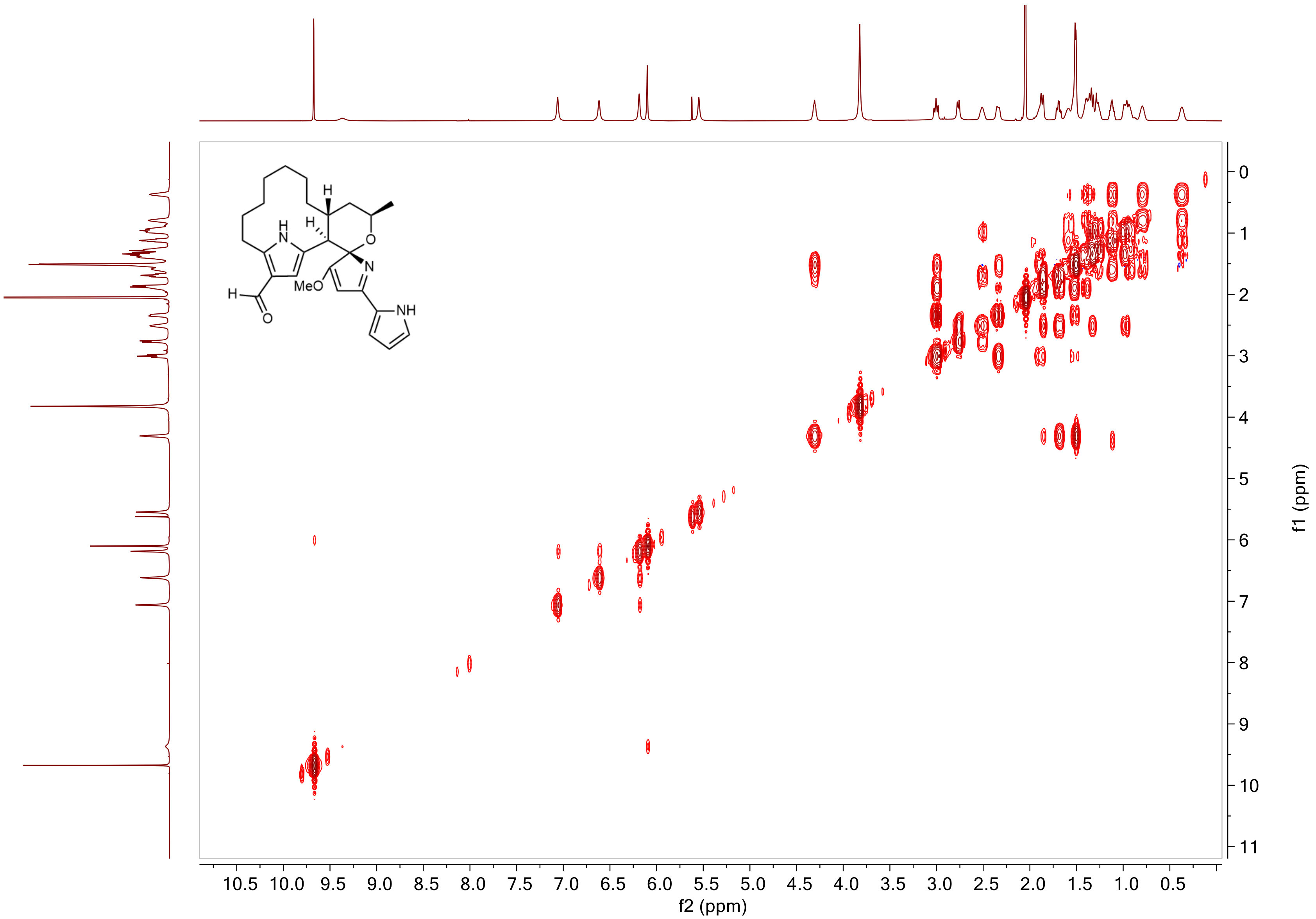

NMR Spectrum 38. ^{1}H-^{1}H COSY NMR Spectrum of 12-formyl premarineosin A (**8**) in acetone-D_6.

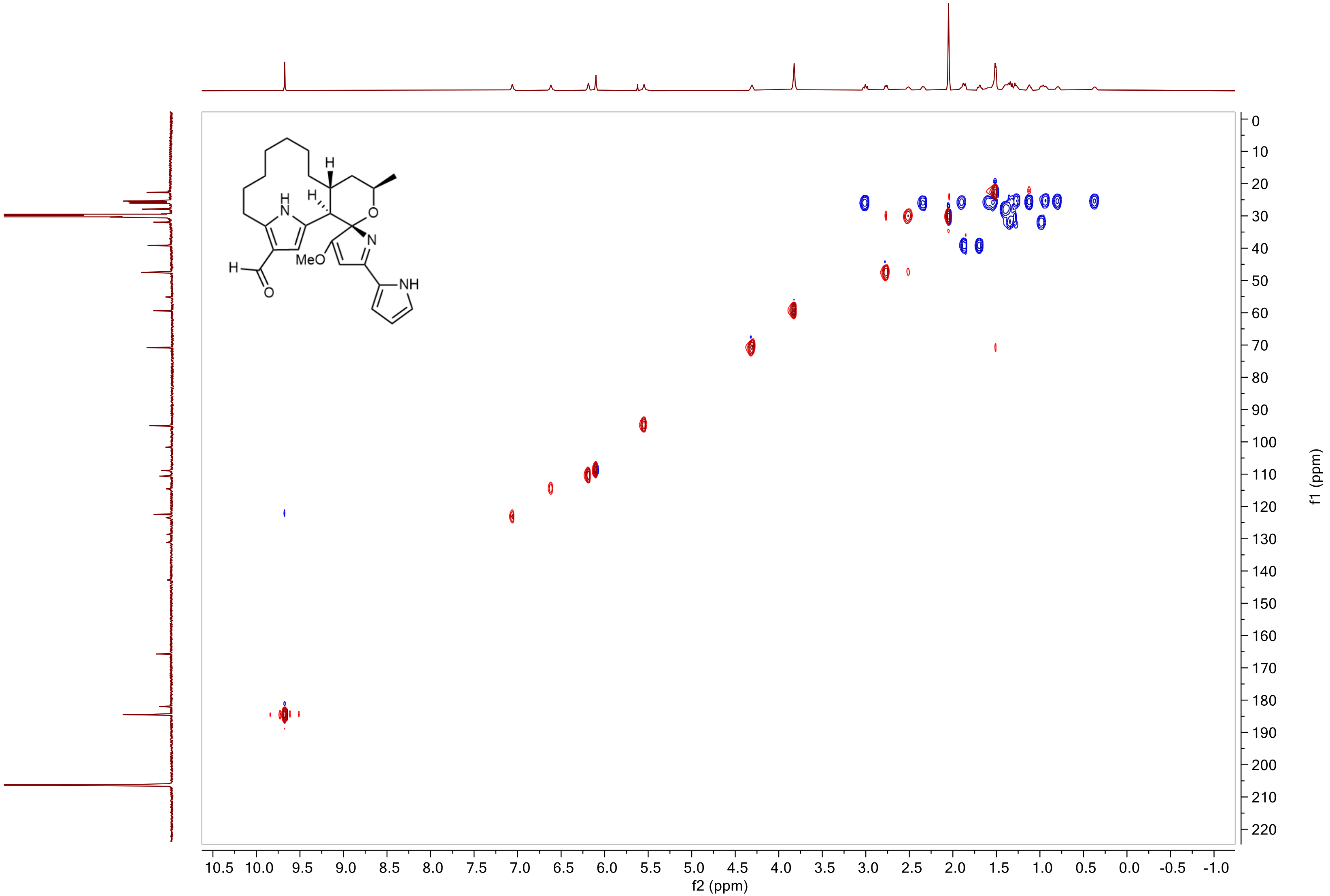

NMR Spectrum 39. ^{1}H-^{13}C HSQC NMR Spectrum of 12-formyl premarineosin A (**8**) in acetone-D_6.

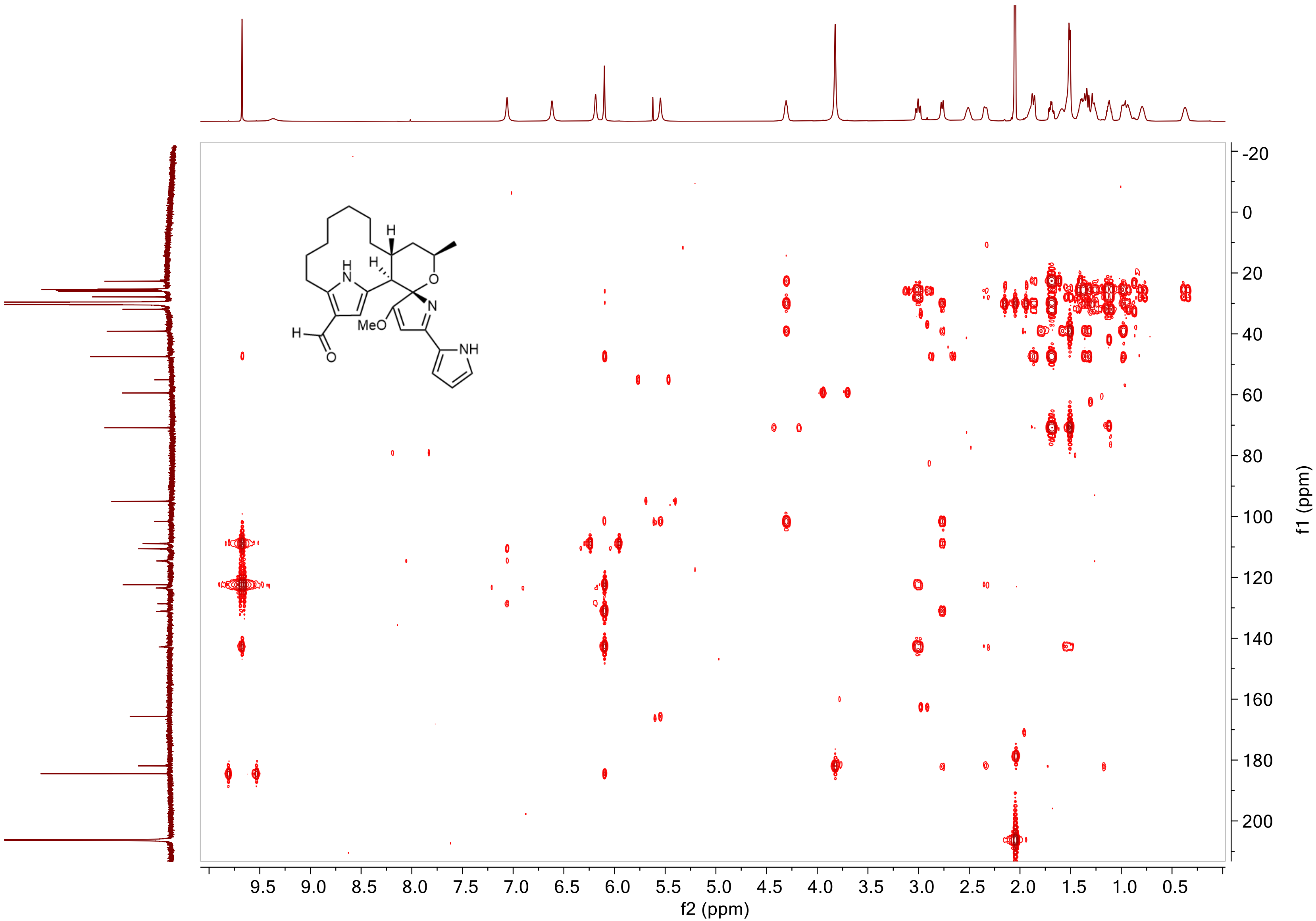

NMR Spectrum 40. ^{1}H-^{13}C HMBC NMR Spectrum of 12-formyl premarineosin A (**8**) in acetone-D_6.

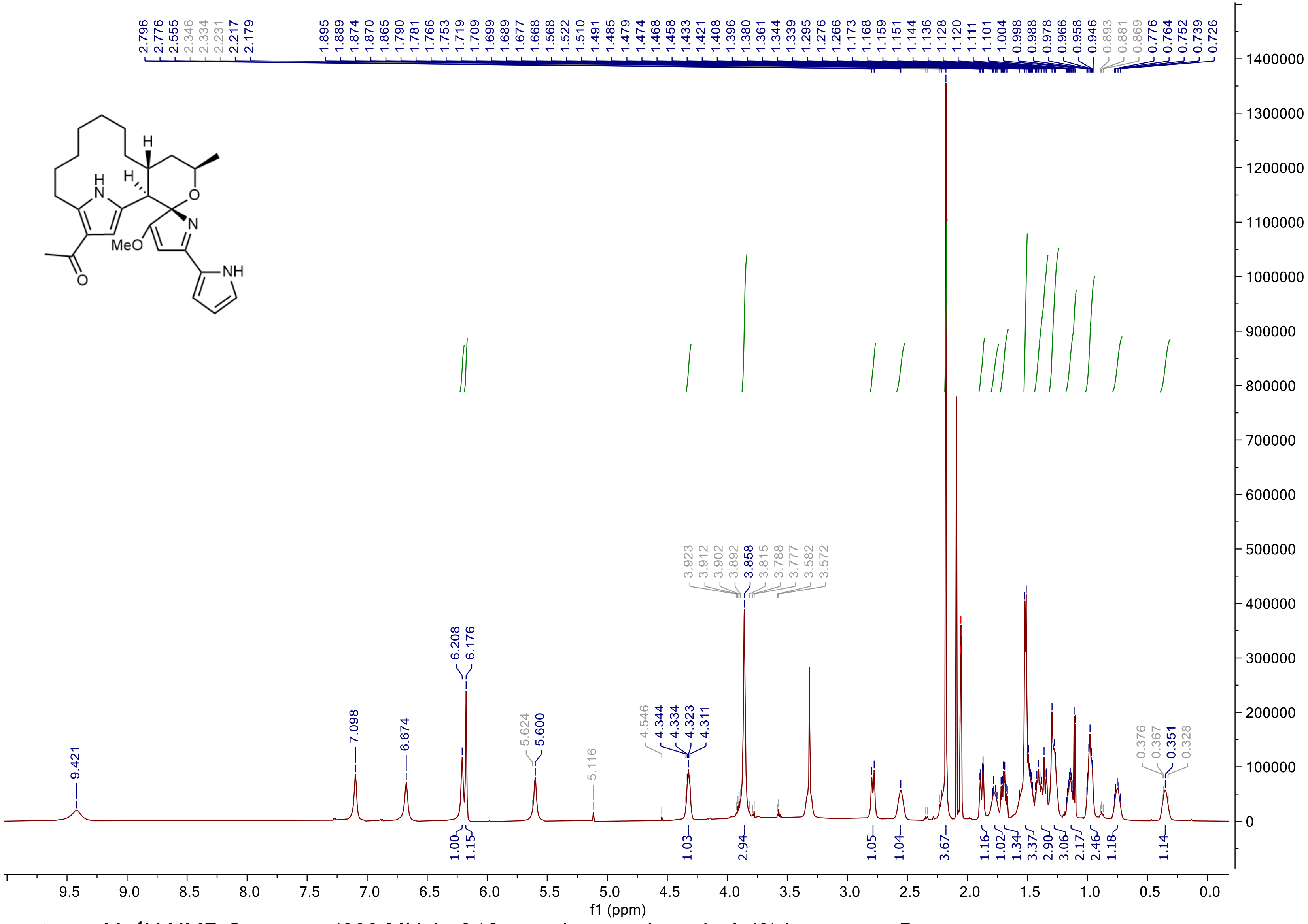

NMR Spectrum 41. ^{1}H NMR Spectrum (600 MHz) of 12-acetyl premarineosin A (**9**) in acetone-D_6.

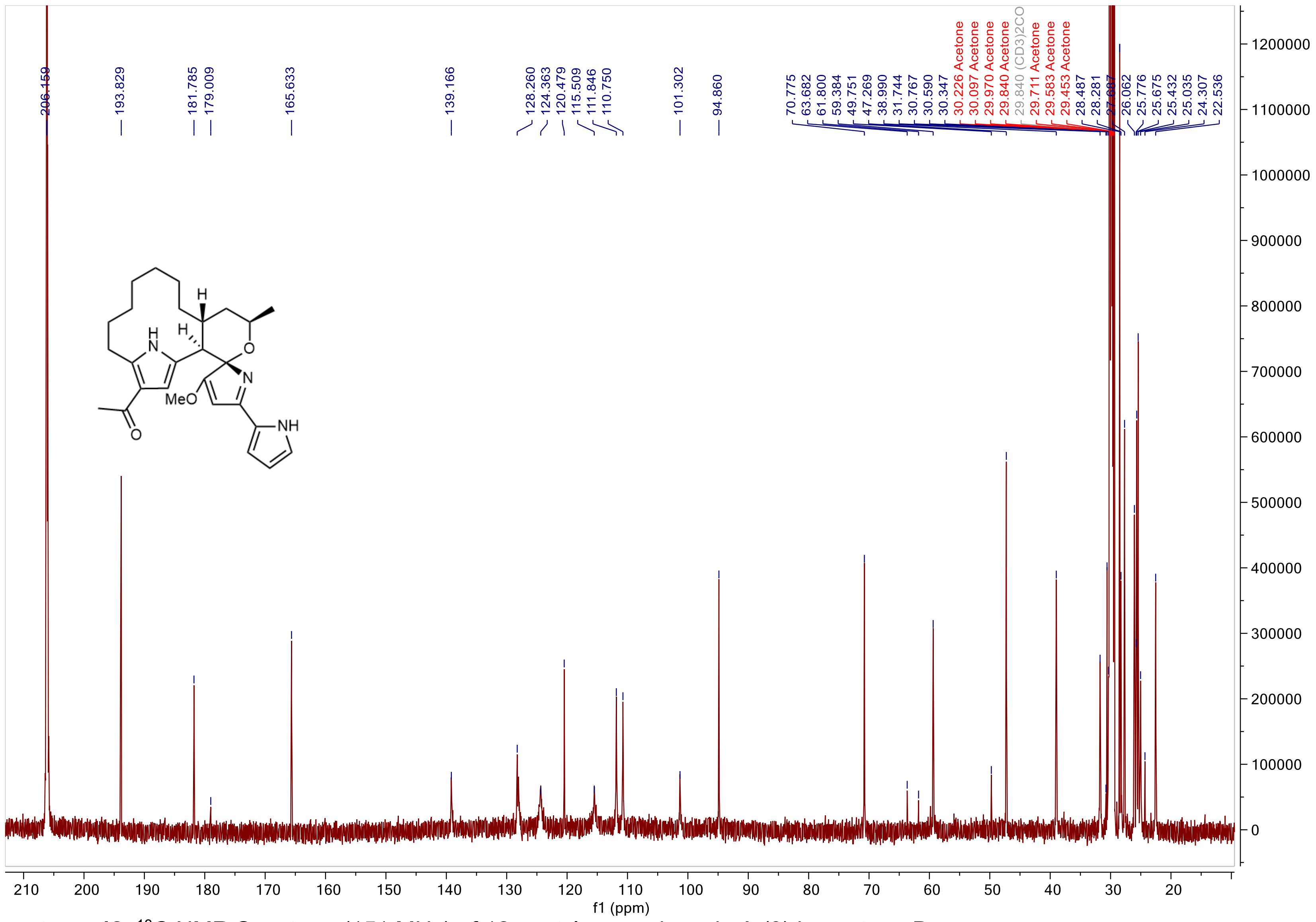

NMR Spectrum 42. ^{13}C NMR Spectrum (151 MHz) of 12-acetyl premarineosin A (**9**) in acetone-D$_6$.

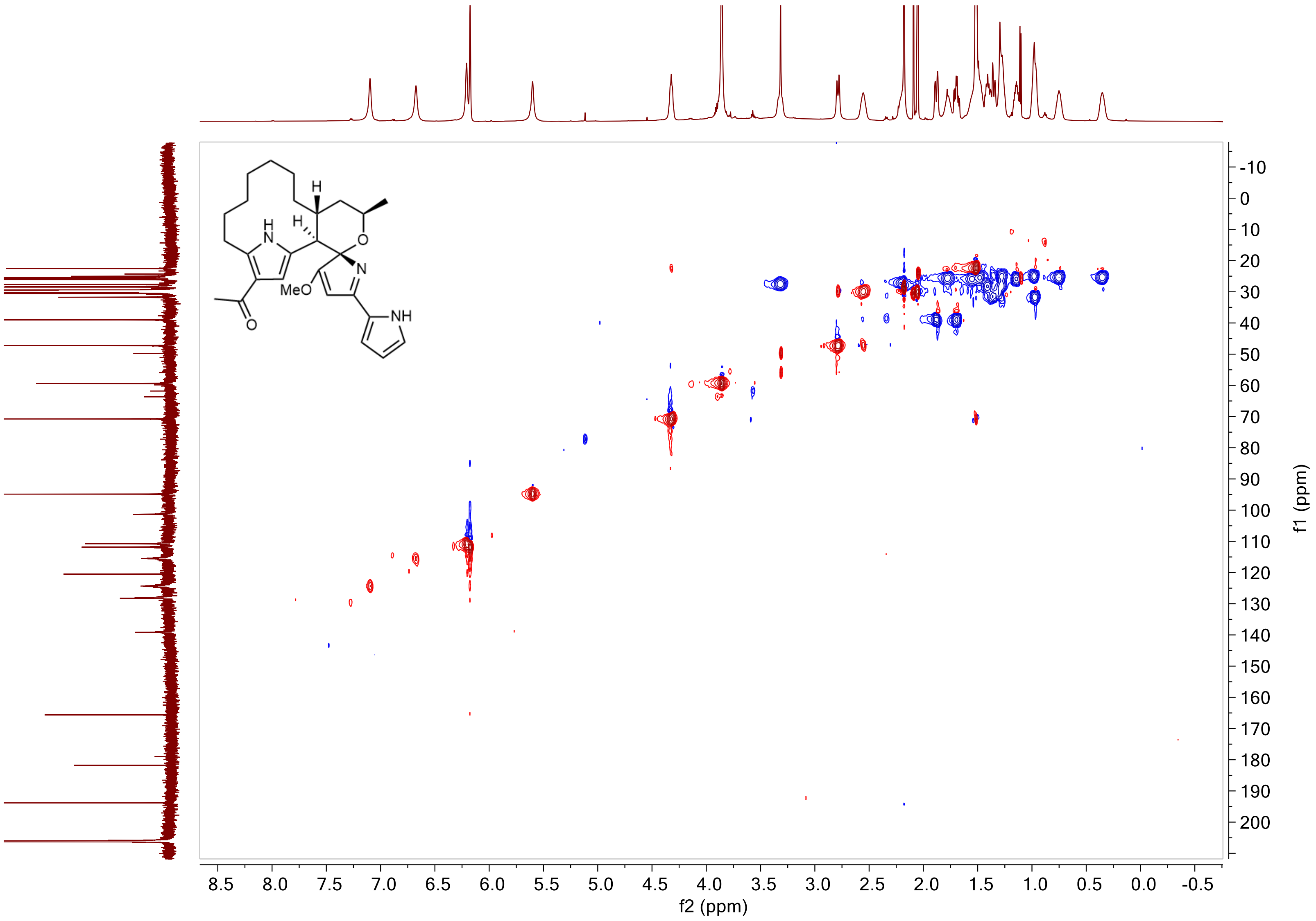

NMR Spectrum 43. ^{1}H-^{13}C HSQC NMR Spectrum of 12-acetyl premarineosin A (**9**) in acetone-D_6.

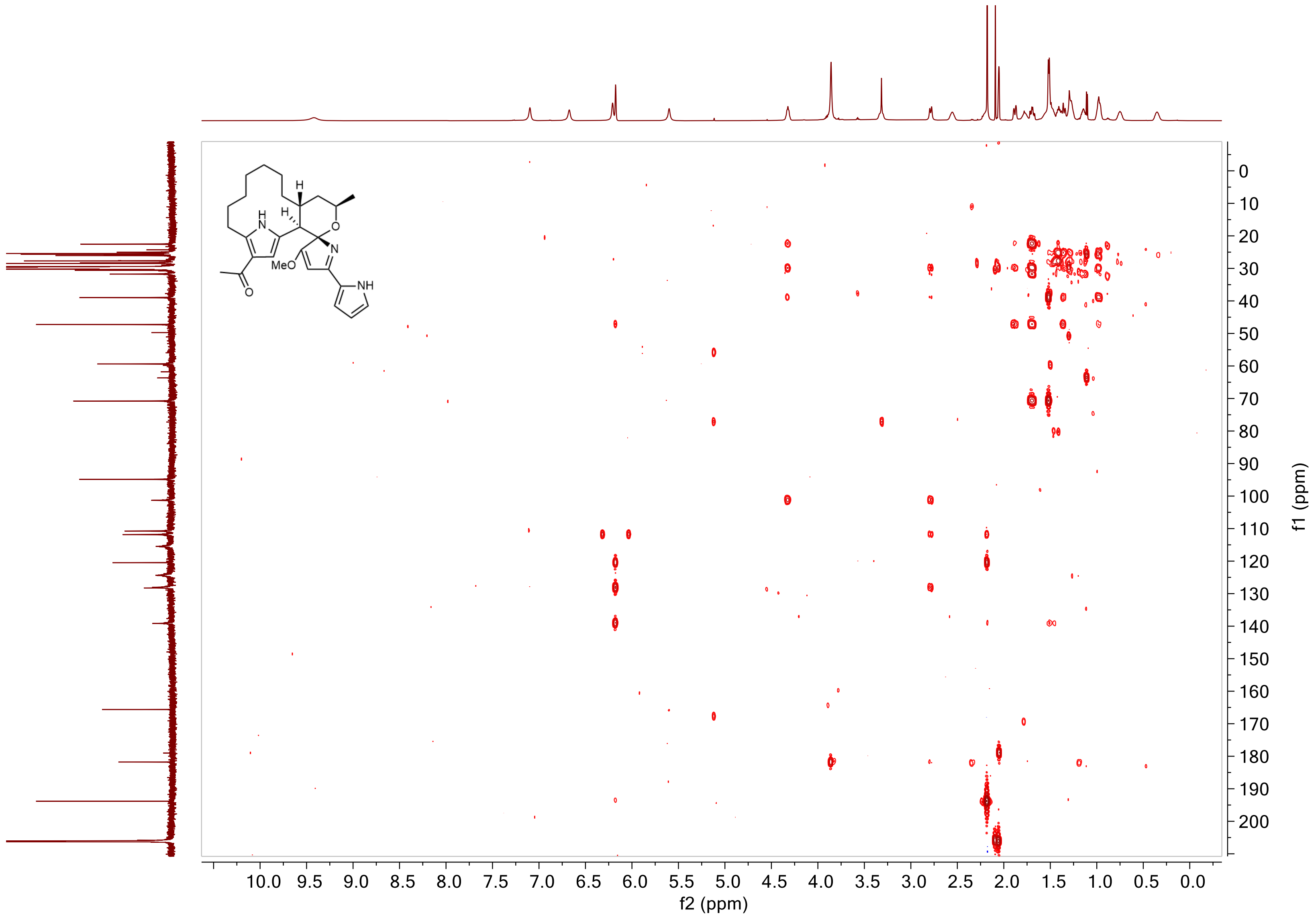

NMR Spectrum 44. ^{1}H-^{13}C HMBC NMR Spectrum of 12-acetyl premarineosin A (**9**) in acetone-D_6.

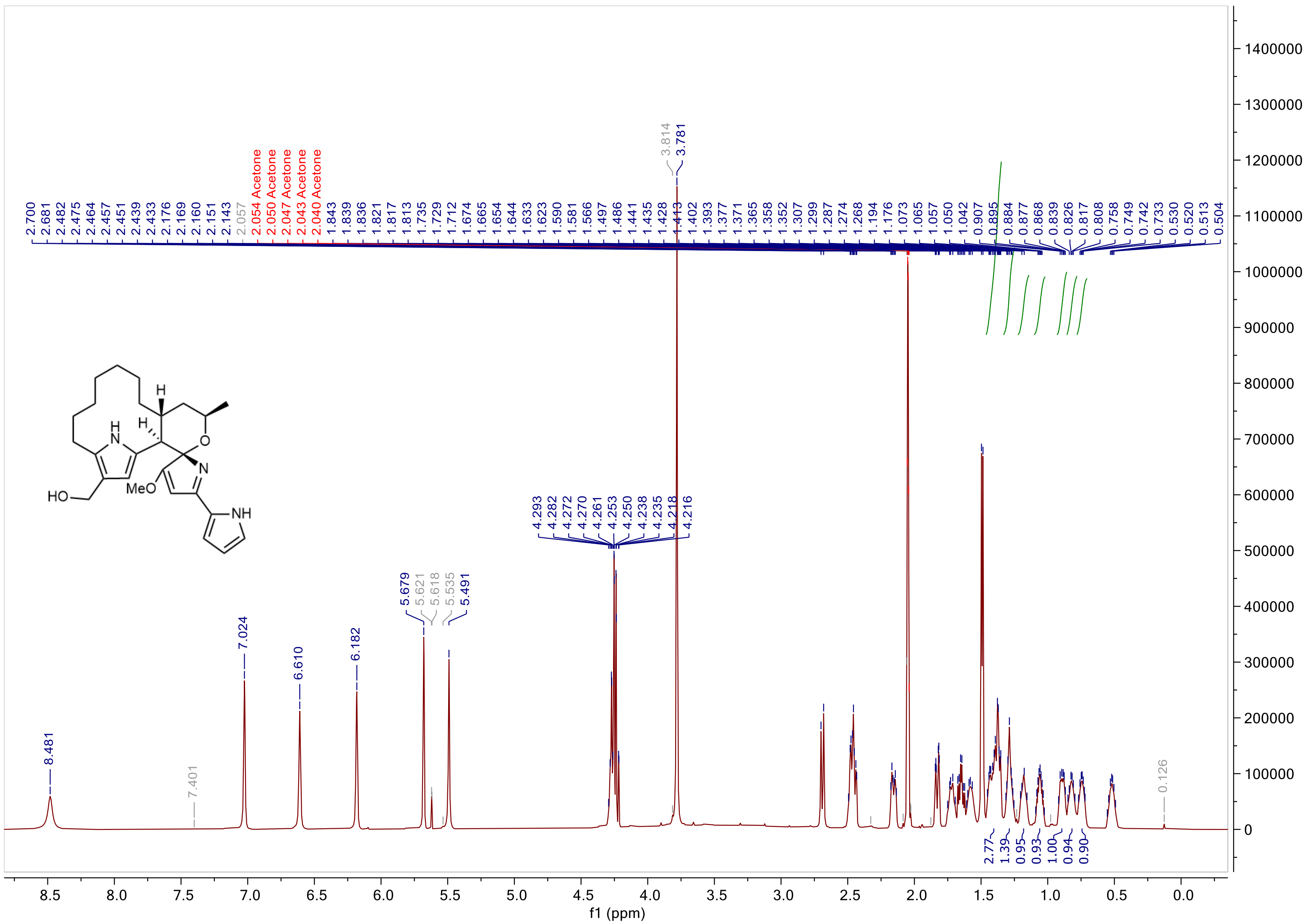

NMR Spectrum 45. ^{1}H NMR Spectrum (600 MHz) of 12-hydroxymethyl premarineosin A (**10**) in acetone-D$_6$.

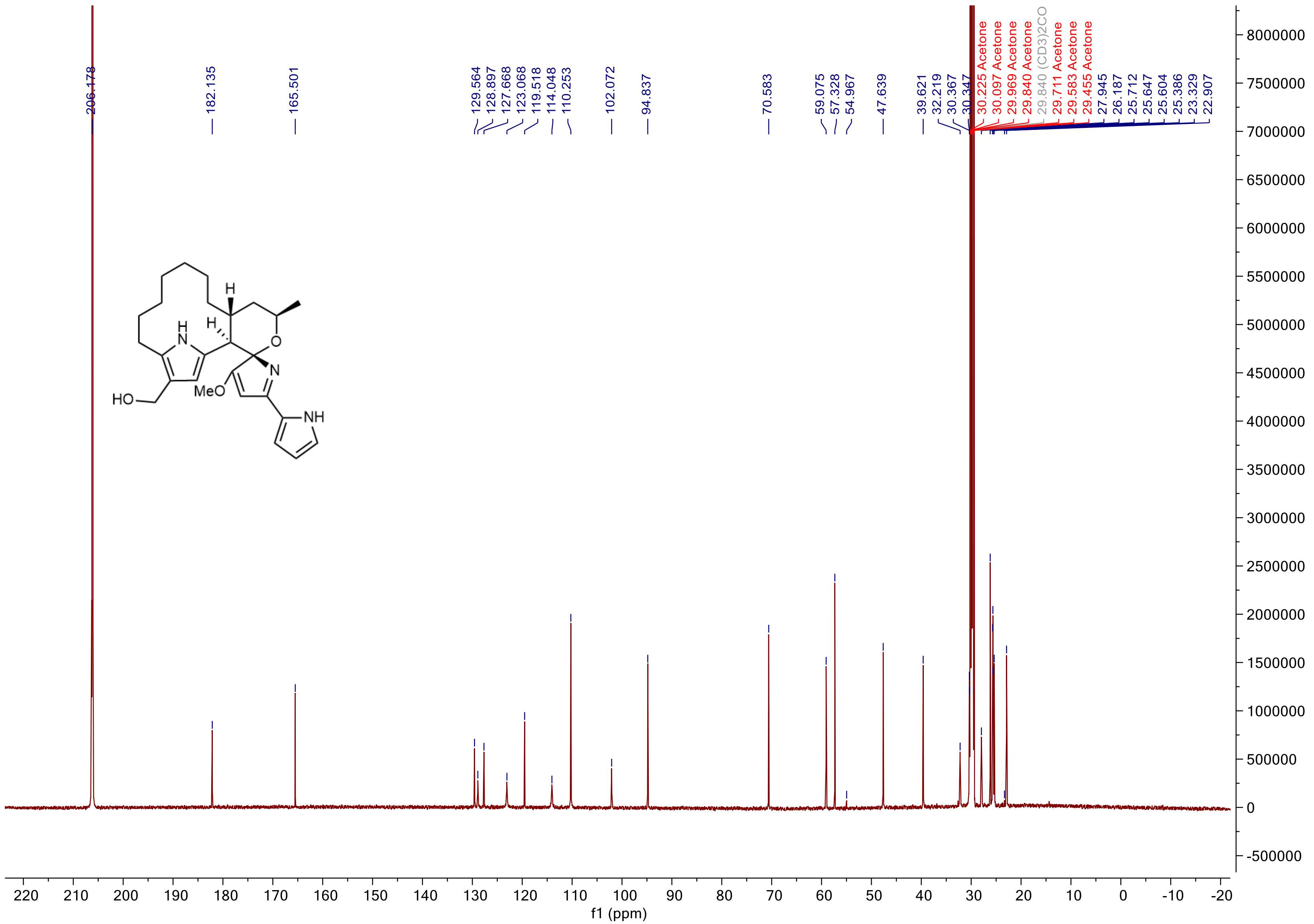

NMR Spectrum 46. ^{13}C NMR Spectrum (151 MHz) of 12-hydroxymethyl premarineosin A (**10**) in acetone-D_6.

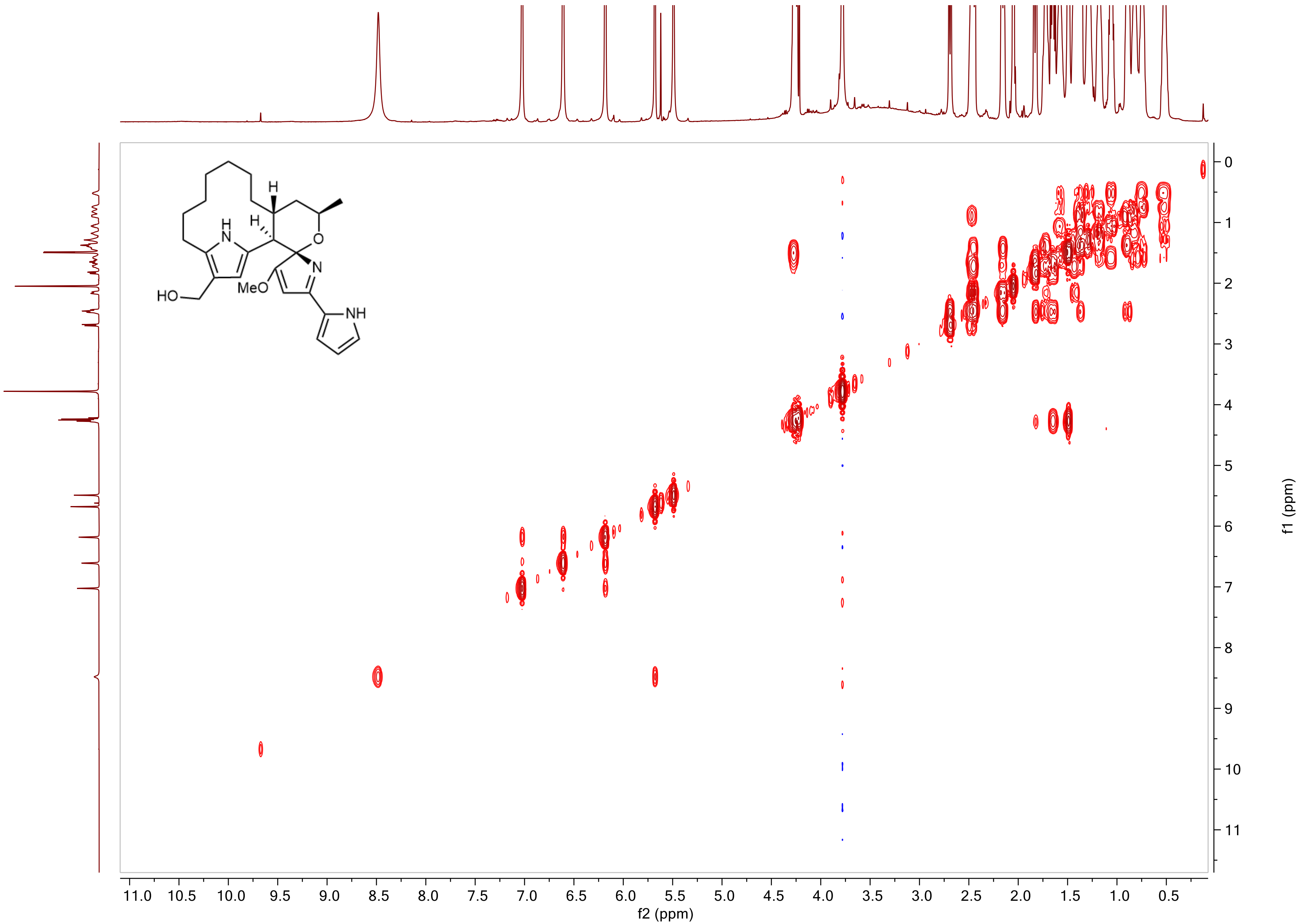

NMR Spectrum 47. ^{1}H-^{1}H COSY NMR Spectrum of 12-hydroxymethyl premarineosin A (**10**) in acetone-D_6.

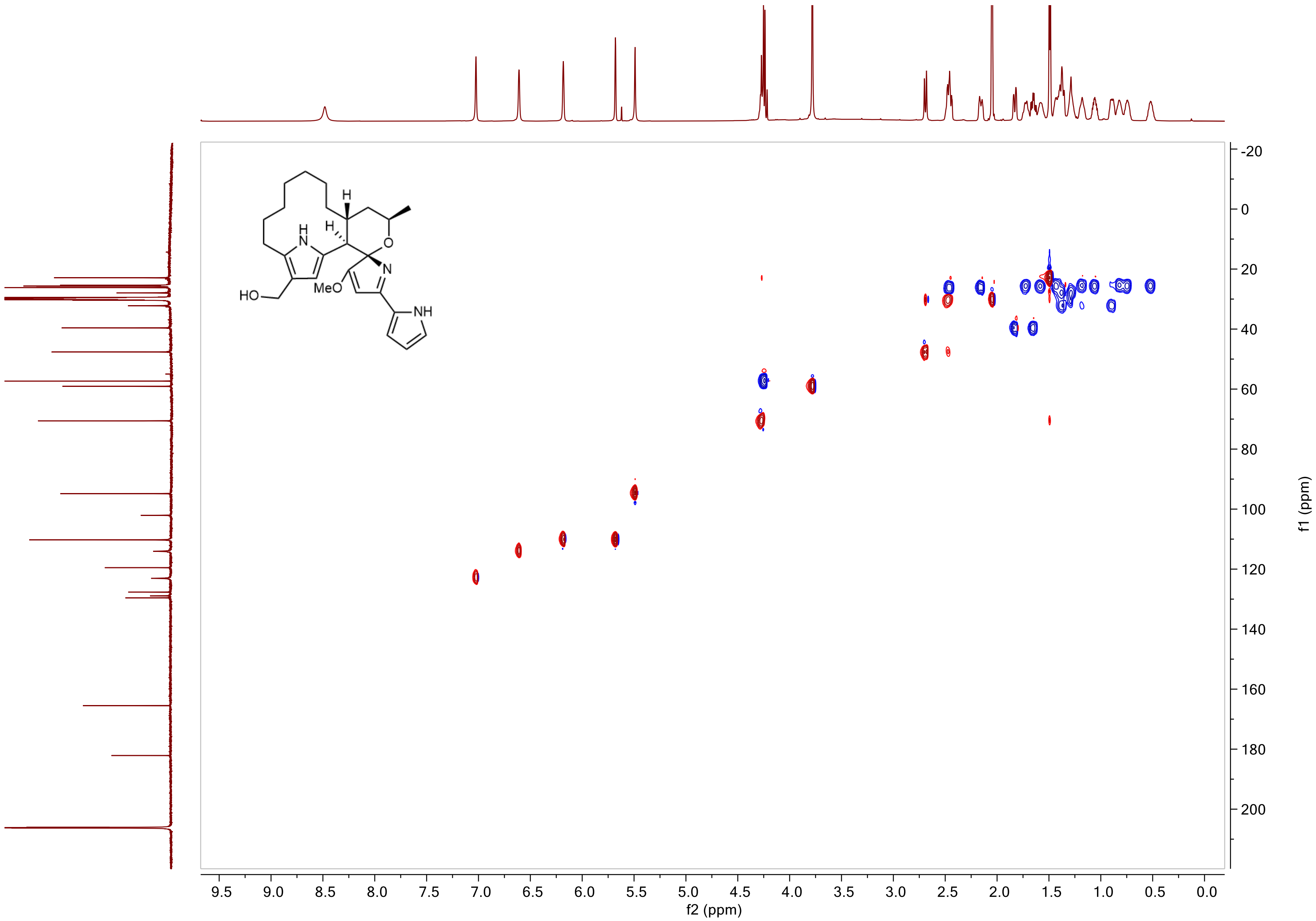

NMR Spectrum 48. ^{1}H-^{13}C HSQC NMR Spectrum of 12-hydroxymethyl premarineosin A (**10**) in acetone-D$_6$.

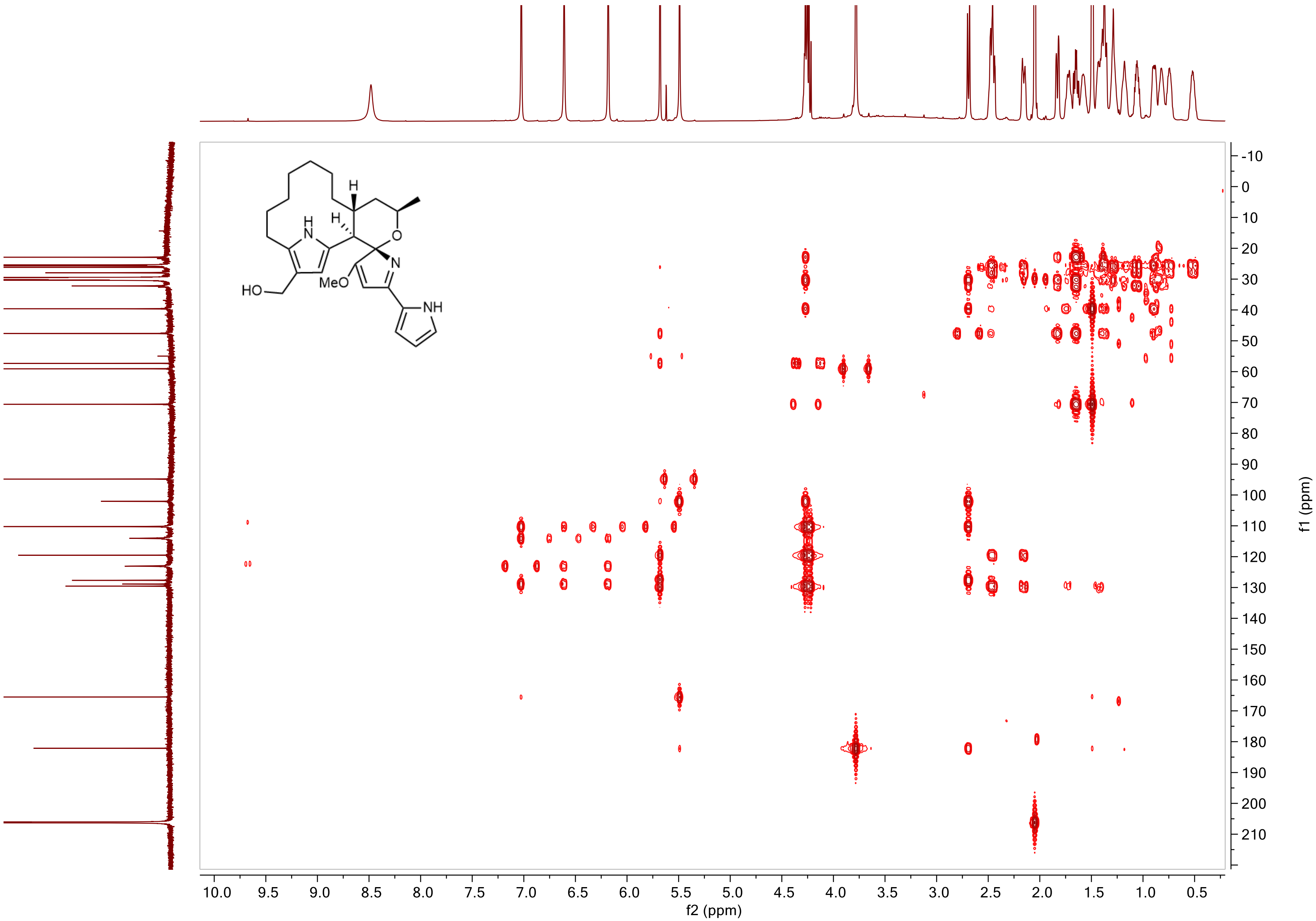

NMR Spectrum 49. ^{1}H-^{13}C HMBC NMR Spectrum of 12-hydroxymethyl premarineosin A (**10**) in acetone-D$_6$.

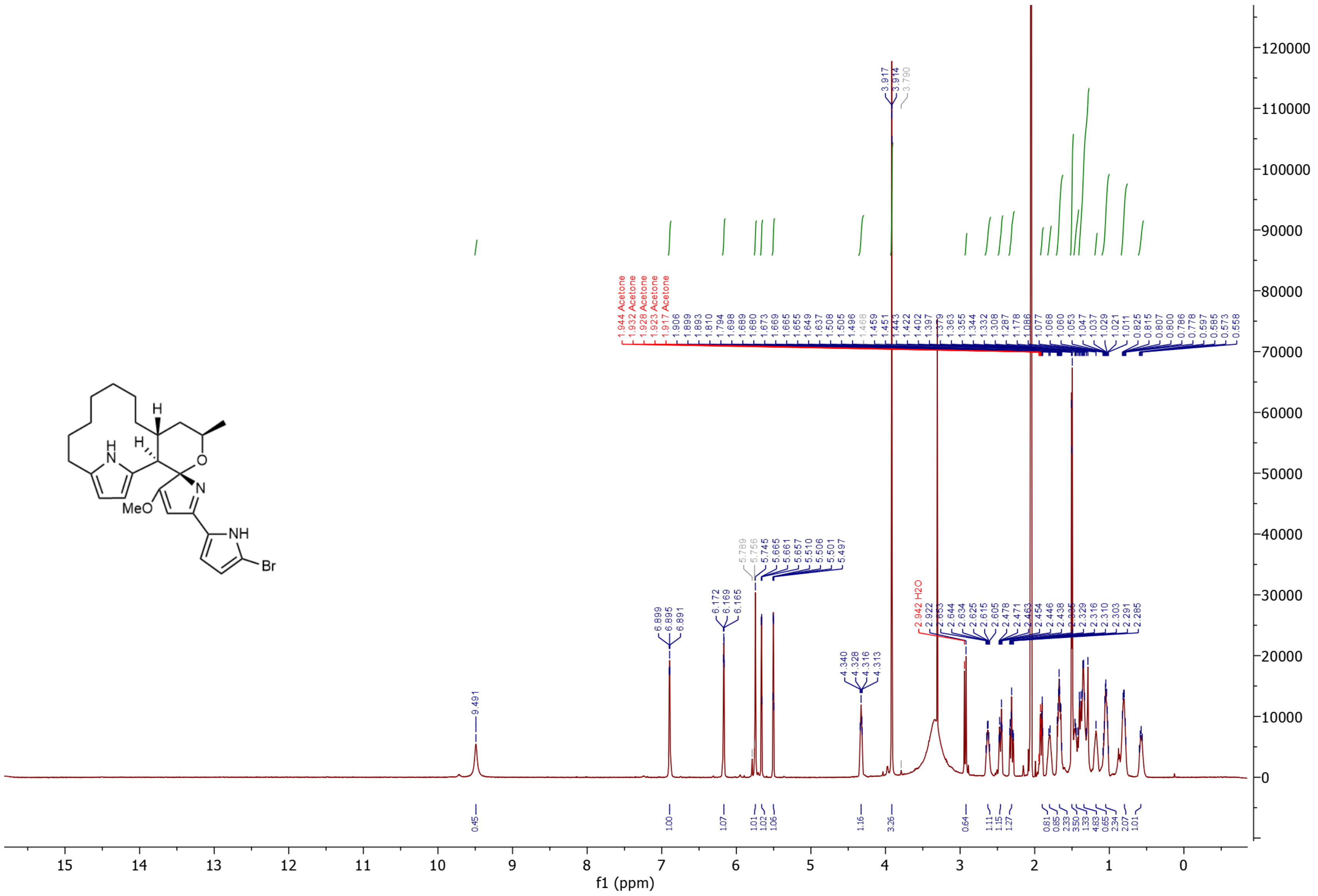

NMR Spectrum 50. ^{1}H NMR Spectrum (600 MHz) of 1-bromo-premarineosin (**11**) in acetone-D_6.

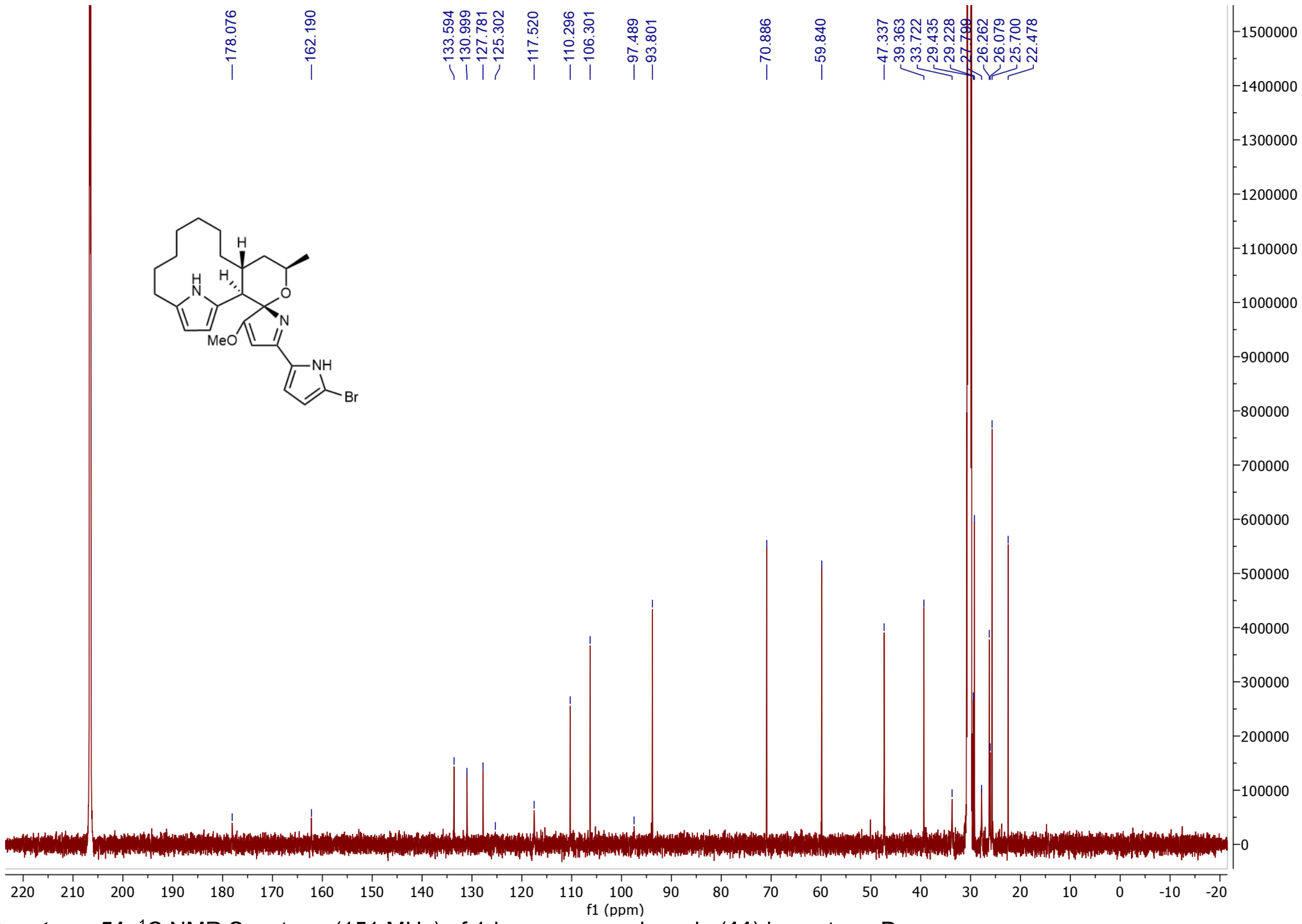

NMR Spectrum 51. ^{1}C NMR Spectrum (151 MHz) of 1-bromo-premarineosin (**11**) in acetone-D$_6$.

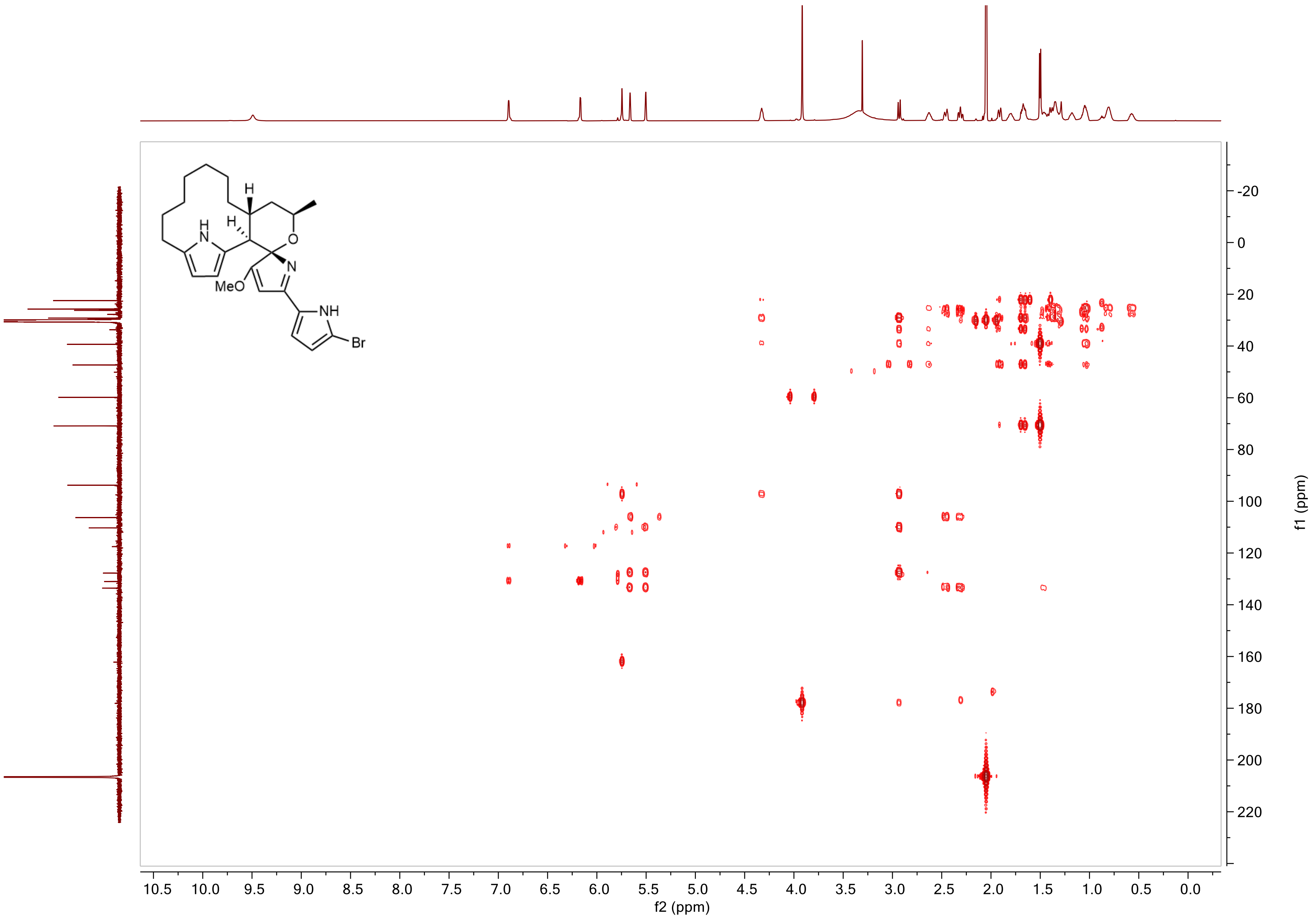

NMR Spectrum 52. ^{1}H-^{13}C HMBC NMR Spectrum of 1-bromo-premarineosin (**11**) in acetone-D$_6$.

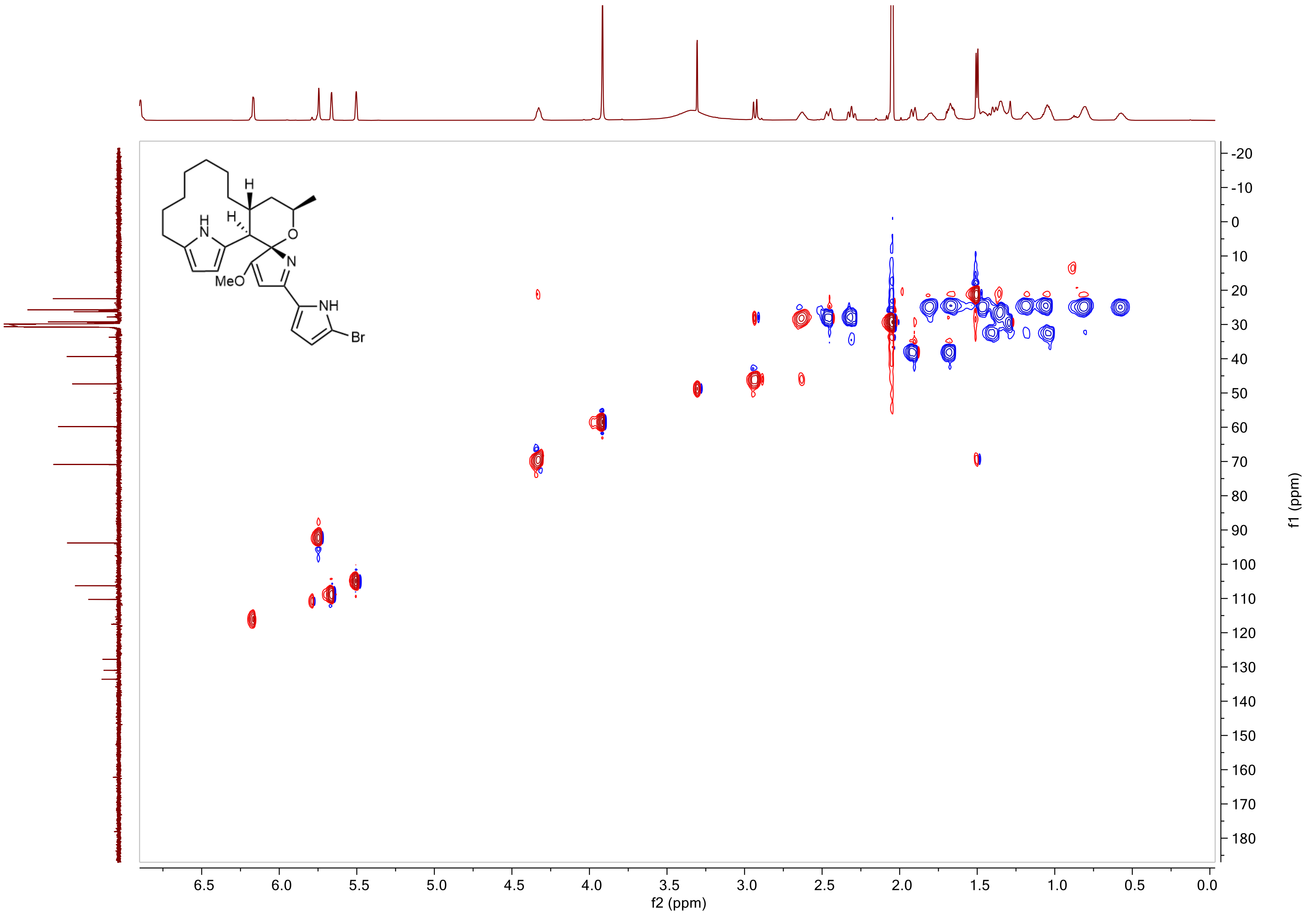

NMR Spectrum 53. ^{1}H-^{13}C HSQC NMR Spectrum of 1-bromo-premarineosin (**11**) in acetone-D_6.

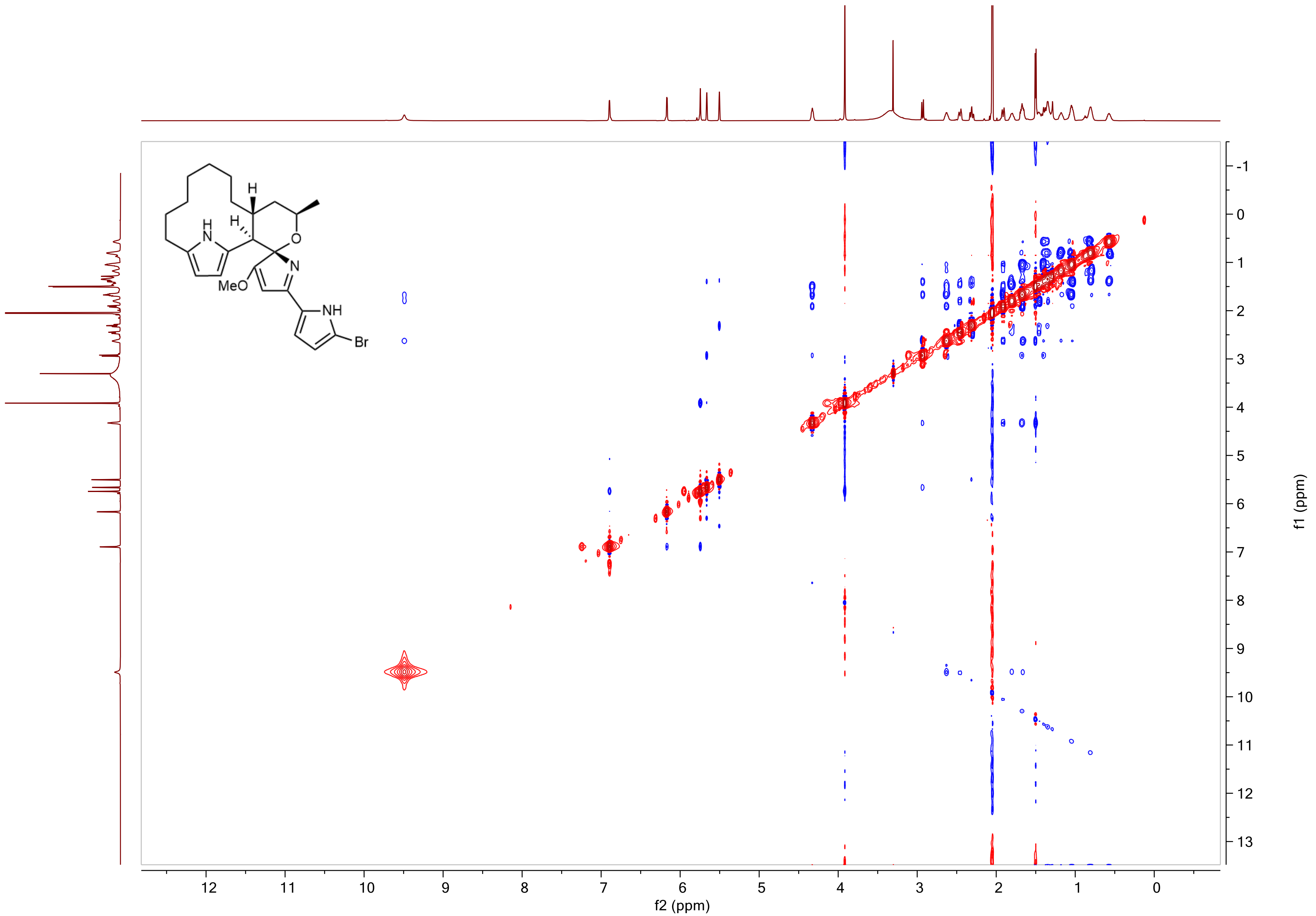

NMR Spectrum 54. ^{1}H-^{13}C NOESY Spectrum of 1-bromo-premarineosin (**11**) in acetone-D$_6$.

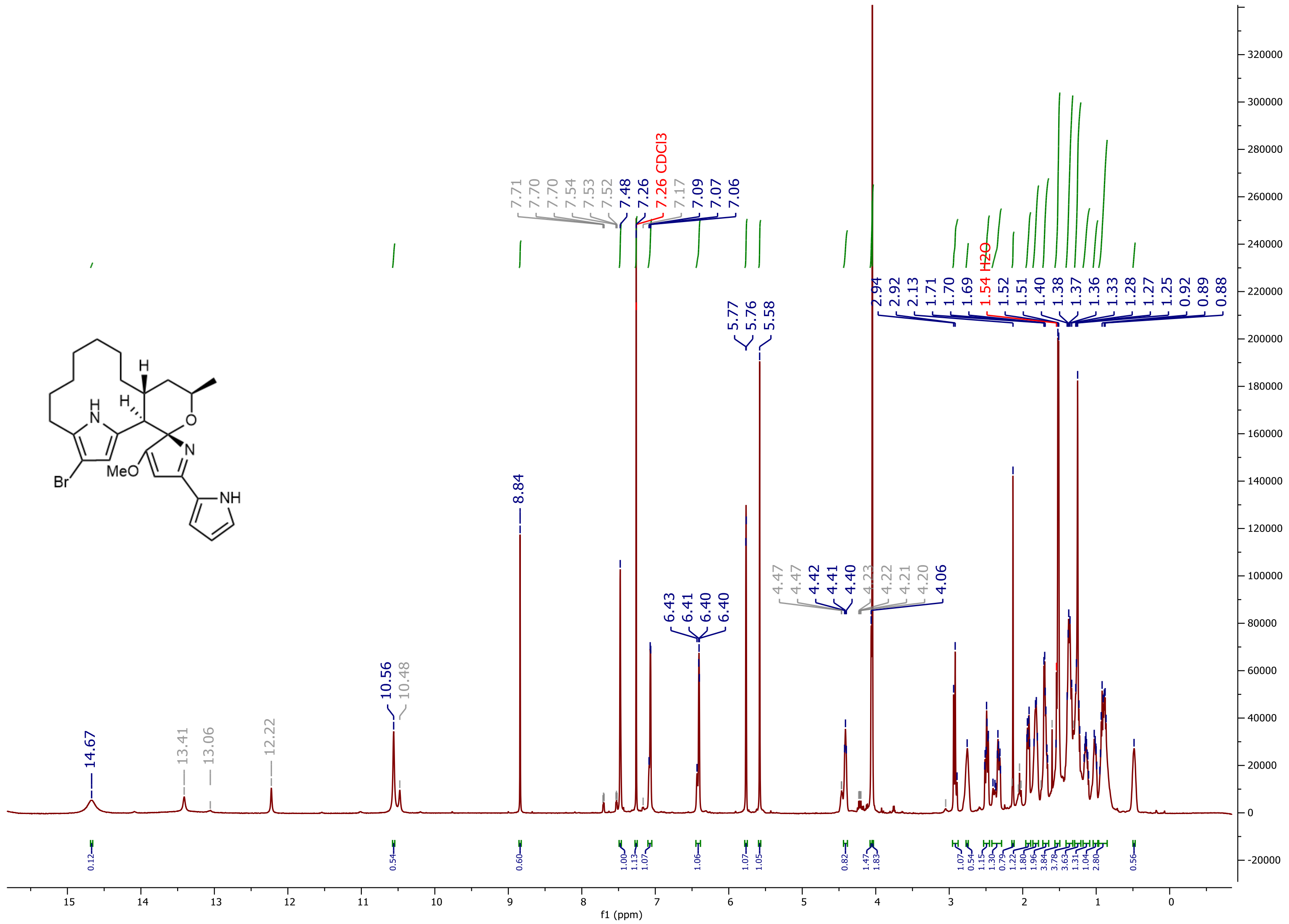

NMR Spectrum 55. ^{1}H NMR Spectrum (600 MHz) of 12-bromo-premarineosin (**12**) in chloroform-D.

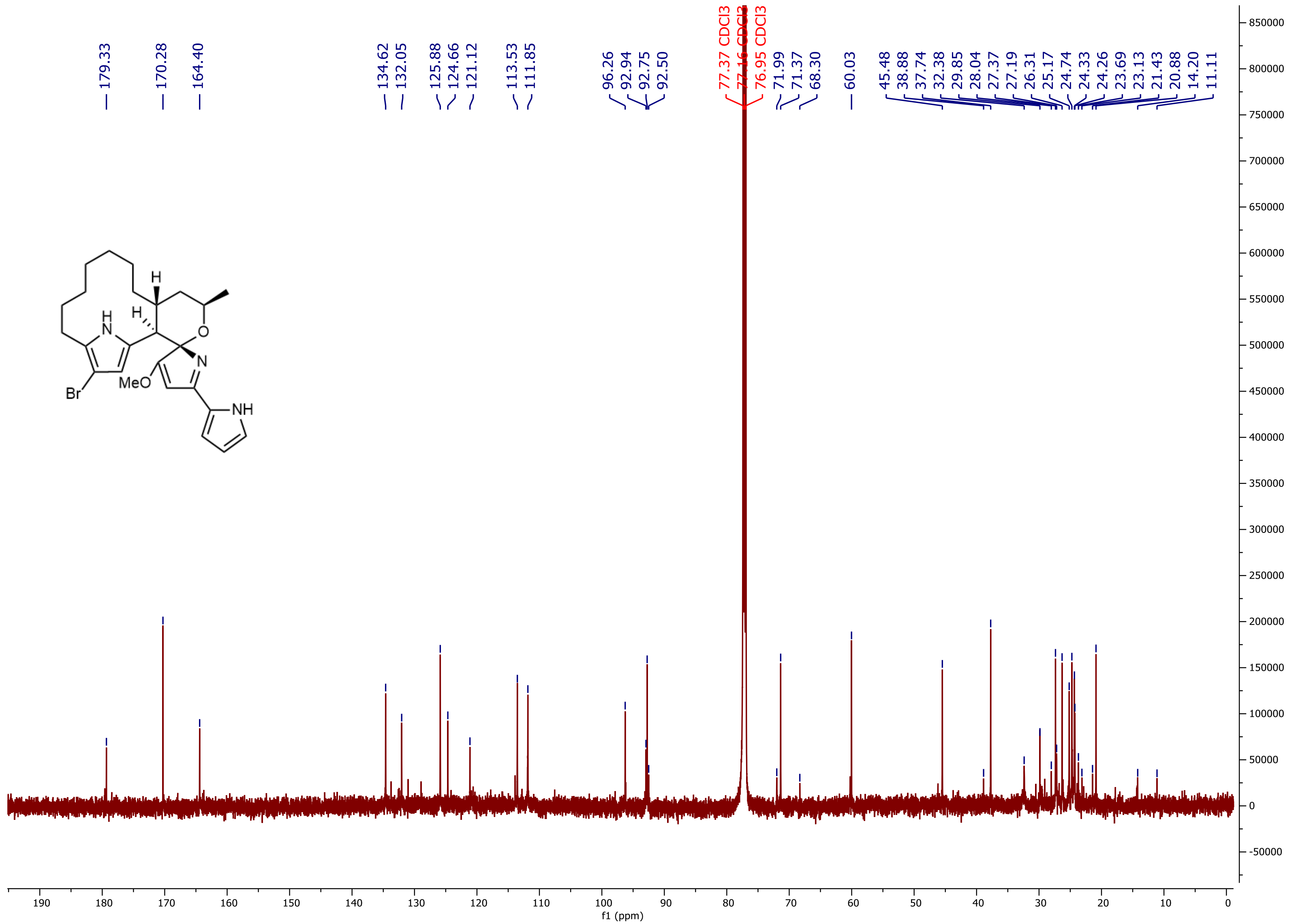

NMR Spectrum 56. ^{1}C NMR Spectrum (151 MHz) of 12-bromo-premarineosin (**12**) in chloroform-D.

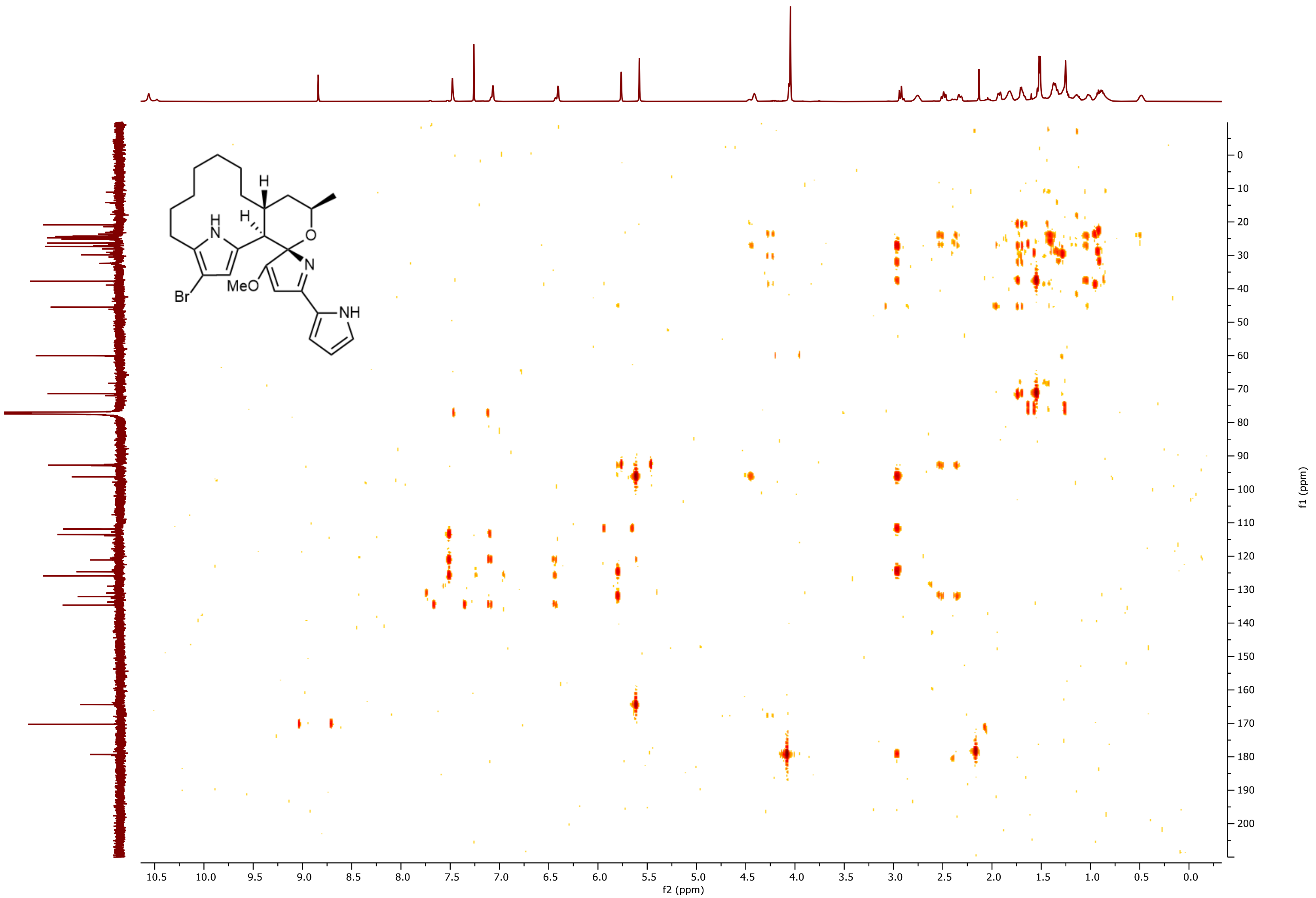

NMR Spectrum 57. ^{1}H-^{13}C HMBC Spectrum of 12-bromo-premarineosin (**12**) in chloroform-D

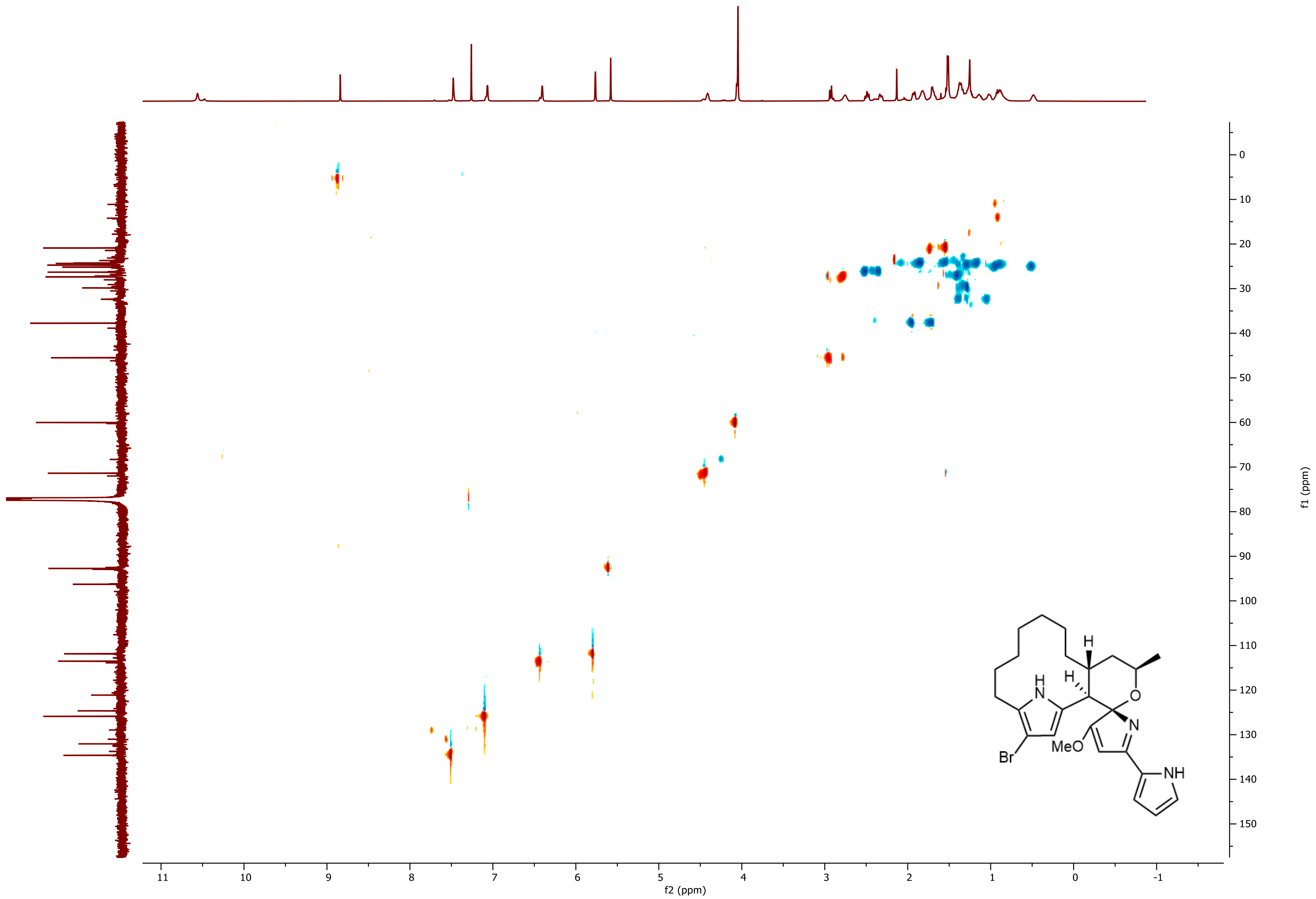

NMR Spectrum 58. ^{1}H-^{13}C HSQC NMR Spectrum of 12-bromo-premarineosin (**12**) in chloroform-D.

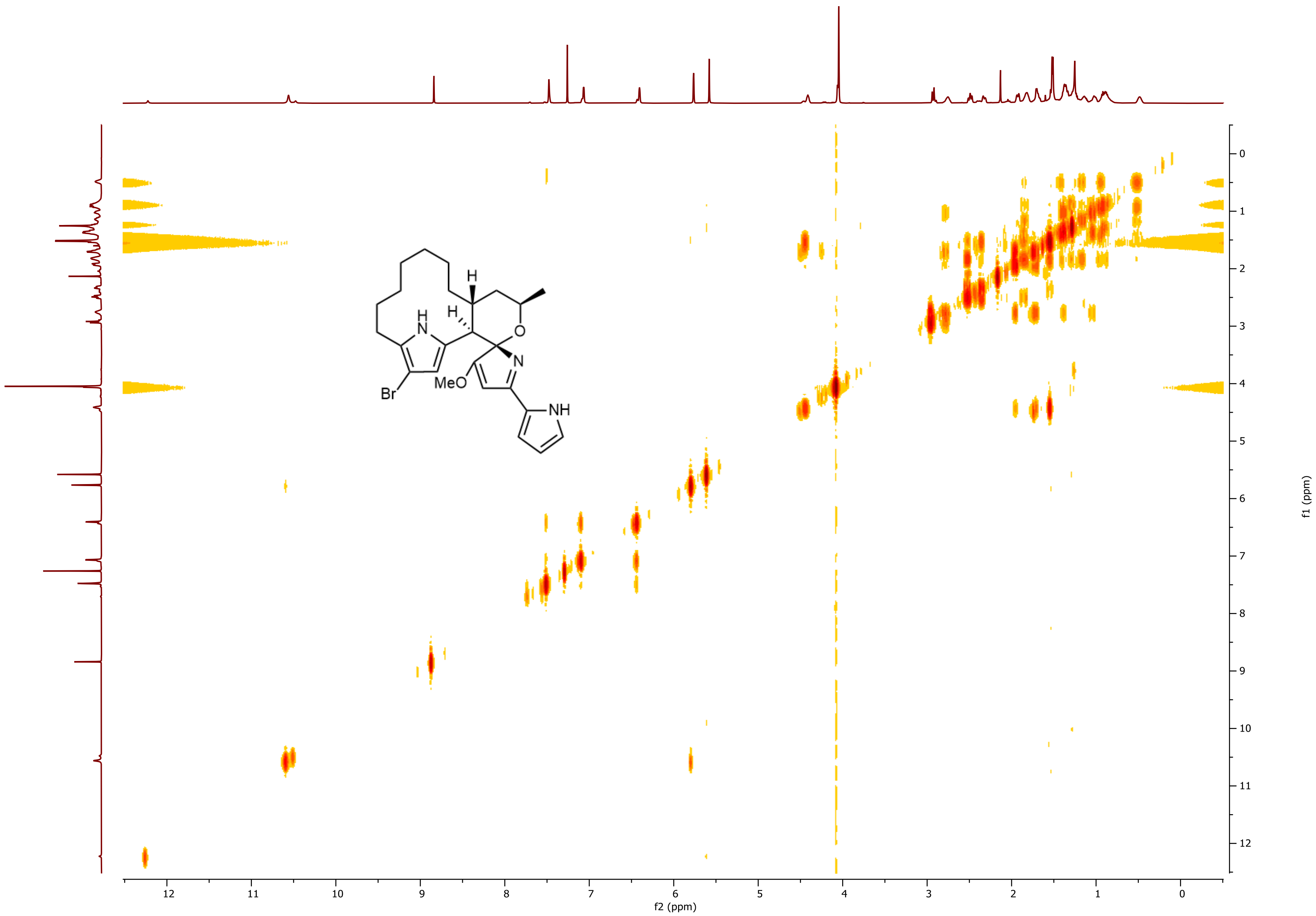

NMR Spectrum 59. ^{1}H-^{1}H COSY NMR Spectrum of 12-bromo-premarineosin (**12**) in chloroform-D.

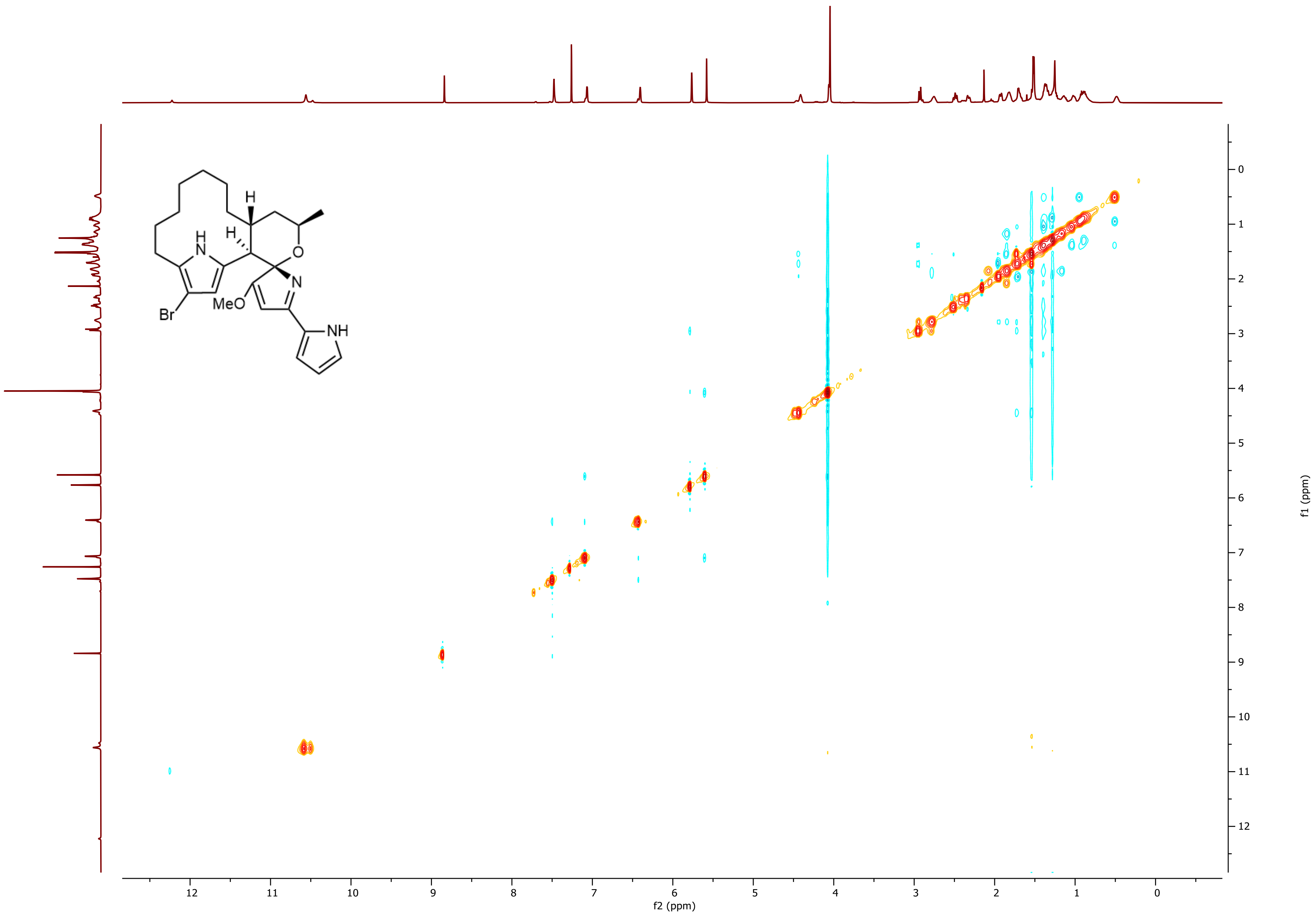

NMR Spectrum 60. 1H-13C NOESY Spectrum of 12-bromo-premarineosin (**12**) in chloroform-D.
