# Supplementary material for: Metabolic engineering and late-stage functionalization expand the chemical space of the antimalarial premarineosin A
Source: Commun Chem. 2025 Dec 5;8:391. doi: 10.1038/s42004-025-01779-6 (PMC12680612; doi:10.1038/s42004-025-01779-6)
Supplement: Supplementary file 7 — Reporting Summary [file 42004_2025_1779_MOESM7_ESM.pdf]

nature portfolio

Corresponding author(s): David H. Sherman, Filipa Pereira, Jared C. Lewis

Last updated by author(s): Oct 22, 2025

Reporting Summary

Nature Portfolio wishes to improve the reproducibility of the work that we publish. This form provides structure for consistency and transparency in reporting. For further information on Nature Portfolio policies, see our Editorial Policies and the Editorial Policy Checklist.

Statistics

For all statistical analyses, confirm that the following items are present in the figure legend, table legend, main text, or Methods section.

n/a	Confirmed	
☐	☒	The exact sample size (*n*) for each experimental group/condition, given as a discrete number and unit of measurement
☐	☒	A statement on whether measurements were taken from distinct samples or whether the same sample was measured repeatedly
☐	☒	The statistical test(s) used AND whether they are one- or two-sided<br>*Only common tests should be described solely by name; describe more complex techniques in the Methods section.*
☒	☐	A description of all covariates tested
☐	☒	A description of any assumptions or corrections, such as tests of normality and adjustment for multiple comparisons
☐	☒	A full description of the statistical parameters including central tendency (e.g. means) or other basic estimates (e.g. regression coefficient) AND variation (e.g. standard deviation) or associated estimates of uncertainty (e.g. confidence intervals)
☐	☒	For null hypothesis testing, the test statistic (e.g. F, t, r) with confidence intervals, effect sizes, degrees of freedom and P value noted<br>*Give P values as exact values whenever suitable.*
☒	☐	For Bayesian analysis, information on the choice of priors and Markov chain Monte Carlo settings
☒	☐	For hierarchical and complex designs, identification of the appropriate level for tests and full reporting of outcomes
☒	☐	Estimates of effect sizes (e.g. Cohen's *d*, Pearson's *r*), indicating how they were calculated

Our web collection on statistics for biologists contains articles on many of the points above.

Software and code

Policy information about availability of computer code

Data collection	Data collection software used in this study included a ViewLux CCD imager (PerkinElmer) for luminescence measurements, an analytical HPLC (Shimadzu) equipped with a PDA detector and analyzed with a Phenyl-Hexyl column for HPLC analysis, a Jasco P2000 polarimeter with a 100 mm cell for optical rotation analysis, a FLUOstar OPTIMA reader (BMG Labtech, Germany) for fluorescence measurements, a Bruker 600 NMR system (600 MHz) for recording NMR spectra, and an ultra-high-performance liquid chromatography coupled with quadrupole time-of-flight mass spectrometry (UHPLC-LCMS) performed with an Agilent 1290 Infinity II UHPLC coupled to an Agilent 6545 ESI-Q-TOF-MS for LC-MS and LC-MS/MS measurements.
Data analysis	Whole genome sequencing data was performed by Plasmidsaurus using long-read Oxford Nanopore Technology and analyzed using the Integrative Genomics Viewer (Windows v. 2.19.4) and clinker (online server). Target compound titer analysis was performed in Excel (Microsoft) and GraphPad Prism (v. 10.4.2). Docking studies were performed using AlphaFold2 (v. 2.2.0), ChemDraw (v. 21.0), Chem3D (v. 21.0), AutoDockVina (v. 1.1.2), AutoDockTools, and PyMOL. Mammalian cell toxicity data normalization and the in vitro phenotypic drug assay of compounds against Pf3D7 and Dd2 were performed in Excel (Microsoft), and concentration response curves were analyzed in Prism 8 (GraphPad software, version 8.1.3).

For manuscripts utilizing custom algorithms or software that are central to the research but not yet described in published literature, software must be made available to editors and reviewers. We strongly encourage code deposition in a community repository (e.g. GitHub). See the Nature Portfolio guidelines for submitting code & software for further information.

Data

Policy information about availability of data

All manuscripts must include a data availability statement. This statement should provide the following information, where applicable:

- Accession codes, unique identifiers, or web links for publicly available datasets
- A description of any restrictions on data availability
- For clinical datasets or third party data, please ensure that the statement adheres to our policy

Crystal structure data has been deposited in the Cambridge Crystallographic Data Centre (CCDC) under deposition numbers CCDC 2455429 for (−)-premarineosin A (3) and CCDC 2455430 for gem-dimethyl-bridged premarineosin A (4). Data generated during this study are included in the published article and in the supplemental information files supplied. All other datasets generated during this study are available from the corresponding authors upon reasonable request.

Research involving human participants, their data, or biological material

Policy information about studies with human participants or human data. See also policy information about sex, gender (identity/presentation), and sexual orientation and race, ethnicity and racism.

Reporting on sex and gender	N/A
Reporting on race, ethnicity, or other socially relevant groupings	N/A
Population characteristics	N/A
Recruitment	N/A
Ethics oversight	N/A

Note that full information on the approval of the study protocol must also be provided in the manuscript.

Field-specific reporting

Please select the one below that is the best fit for your research. If you are not sure, read the appropriate sections before making your selection.

☒ Life sciences ☐ Behavioural & social sciences ☐ Ecological, evolutionary & environmental sciences

For a reference copy of the document with all sections, see nature.com/documents/nr-reporting-summary-flat.pdf

Life sciences study design

All studies must disclose on these points even when the disclosure is negative.

Sample size	Experimental sample sizes are described in respective figure legends and methods.
Data exclusions	No data points were excluded.
Replication	Target compound titers were calculated from three biological replicates. The mammalian cellular toxicity experiments were conducted as four technical replicates. The in vitro phenotypic drug assay of compounds against Pf3D7 and Dd2 was performed in two independent experiments in triplicate, and data was reproducible. All attempts at replication were successful.
Randomization	Randomization in titrated compound responses is generally not required as independent variables are controlled for in the experimental design, for example, by including DMSO vehicle wells to allow for correction and normalization of plate field variations that could arise from, for example plate edge effects. The use of full compound titrations at all stages of this work further reduces potential for random variation affecting the objective outcomes of these data.
Blinding	Compound identity was blinded as coded ids to scientists conducting in vitro experiments.

Reporting for specific materials, systems and methods

We require information from authors about some types of materials, experimental systems and methods used in many studies. Here, indicate whether each material, system or method listed is relevant to your study. If you are not sure if a list item applies to your research, read the appropriate section before selecting a response.

Materials & experimental systems

n/a	Involved in the study
☒	☐ Antibodies
☐	☒ Eukaryotic cell lines
☒	☐ Palaeontology and archaeology
☒	☐ Animals and other organisms
☒	☐ Clinical data
☒	☐ Dual use research of concern
☒	☐ Plants

Methods

n/a	Involved in the study
☒	☐ ChIP-seq
☒	☐ Flow cytometry
☒	☐ MRI-based neuroimaging

Eukaryotic cell lines

Policy information about cell lines and Sex and Gender in Research

Cell line source(s)	HEK293 and MOLT4 cell lines purchased from ATCC [HEK293 (ATCC, cat # CRL-1573), MOLT-4 (ATCC, cat # CRL-1582].
Authentication	By vendor.
Mycoplasma contamination	Cell cultures were routinely tested for Mycoplasma contamination using the MycoAlert PLUS Mycoplasma Detection Kit (Lonza Bioscience, cat # LT07) according to manufacturer protocol. Cells in this study tested negative for Mycoplasma.
Commonly misidentified lines (See ICLAC register)	There were no commonly misidentified lines used in this study.

Plants

Seed stocks	*Report on the source of all seed stocks or other plant material used. If applicable, state the seed stock centre and catalogue number. If plant specimens were collected from the field, describe the collection location, date and sampling procedures.*
Novel plant genotypes	*Describe the methods by which all novel plant genotypes were produced. This includes those generated by transgenic approaches, gene editing, chemical/radiation-based mutagenesis and hybridization. For transgenic lines, describe the transformation method, the number of independent lines analyzed and the generation upon which experiments were performed. For gene-edited lines, describe the editor used, the endogenous sequence targeted for editing, the targeting guide RNA sequence (if applicable) and how the editor was applied.*
Authentication	*Describe any authentication procedures for each seed stock used or novel genotype generated. Describe any experiments used to assess the effect of a mutation and, where applicable, how potential secondary effects (e.g. second site T-DNA insertions, mosiacism, off-target gene editing) were examined.*
